# Antimicrobial removal on piglets promotes health and higher bacterial diversity in the nasal microbiota

**DOI:** 10.1038/s41598-019-43022-y

**Published:** 2019-04-25

**Authors:** Florencia Correa-Fiz, José Maurício Gonçalves dos Santos, Francesc Illas, Virginia Aragon

**Affiliations:** 1 IRTA, Centre de Recerca en Sanitat Animal (CReSA, IRTA-UAB), Campus de la Universitat Autònoma de Barcelona, 08193 Bellaterra, Spain; 2 Unicesumar-Centro Universitário de Maringá, Maringá, Paraná, Brazil; 3 Selección Batallé, Avinguda dels segadors, 17421 Riudarenes, Spain

**Keywords:** Microbial communities, Classification and taxonomy

## Abstract

The view on antimicrobials has dramatically changed due to the increased knowledge on the importance of microbiota composition in different body parts. Antimicrobials can no longer be considered only beneficial, but also potentially deleterious for favourable bacterial populations. Still, the use of metaphylactic antimicrobial treatment at early stages of life is a practice in use in porcine production. Many reports have shown that antibiotics can critically affect the gut microbiota, however the effect of perinatal antimicrobial treatment on the nasal microbiota has not been explored yet. To gain insights on the potential changes in nasal microbial composition due to antimicrobial treatments, piglets from two different farms were sampled at weaning. The nasal microbiota was analysed when antimicrobial treatment was used early in life, and later, when no antimicrobial treatment was used during the lactation period. Removal of perinatal antimicrobials resulted in an increased bacterial diversity in nasal microbiota at weaning. Concurrently, elimination of antimicrobials produced an increase in the relative abundance of *Prevotella* and *Lactobacillus*, and a decrease in *Moraxella* and *Bergeyella*. These changes in microbiota composition were accompanied by an improvement of the piglets’ health and a higher productivity in the nursery phase.

## Introduction

The use of antimicrobials to control bacterial diseases is a common practice in porcine production. Despite the undeniable fact that usage of antimicrobials plays a vital role in the production of food-animals and in protection of public health^1^, they have proved to negatively affect the beneficial microbiota^2^. The microbiota is a key factor for the well function and homeostasis of different body systems in animals. Key functions of the microbiota include the correct development of the immune system and resistance against pathogens colonization or pathogenicity reduction^3–5^. The interest on animal microbiota has increased in recent years due to its impact in health. Knowledge on microbiota composition can result in the identification of potential bacterial groups associated with health^5–7^, although many factors can interfere in the establishment of a so-called adequate microbiota, such as environment, pig production system, pig genetics and antimicrobial treatments^8^. Microbial dysbiosis, which may appear secondarily to antimicrobial use, can facilitate pathogen infections and enhance the tissue damage inflicted by pathogenic bacteria^9^. The use of metaphylactic antimicrobials, especially early in life, can have a deleterious impact in animal health through the alteration of the gastrointestinal tract (GIT) microbiota composition^10^. Many efforts have been made to elucidate the GIT microbiota composition and the relationship of dysbiosis with many diseases, however, there is scarce knowledge on the nasal microbiota composition in animals. In pigs, it has been demonstrated that the microbial communities inhabiting the nasal cavities at weaning may influence the development of Glässer’s disease later in life^6^ and perinatal antimicrobials are sometimes used to reduce the risk of this disease. However, whether the antibiotic treatment early in life has an effect on the bacterial communities from the piglet’s nose, has not been explored yet. Here, we analysed the nasal microbiota composition of 3–4 week old piglets at weaning regarding antimicrobial administration in the lactation phase, with the aim of detecting the effect on the microbiota compositions and the association with health status later in life.

## Results

### Diversity and species richness from the nasal microbiota

**Figure 1:**
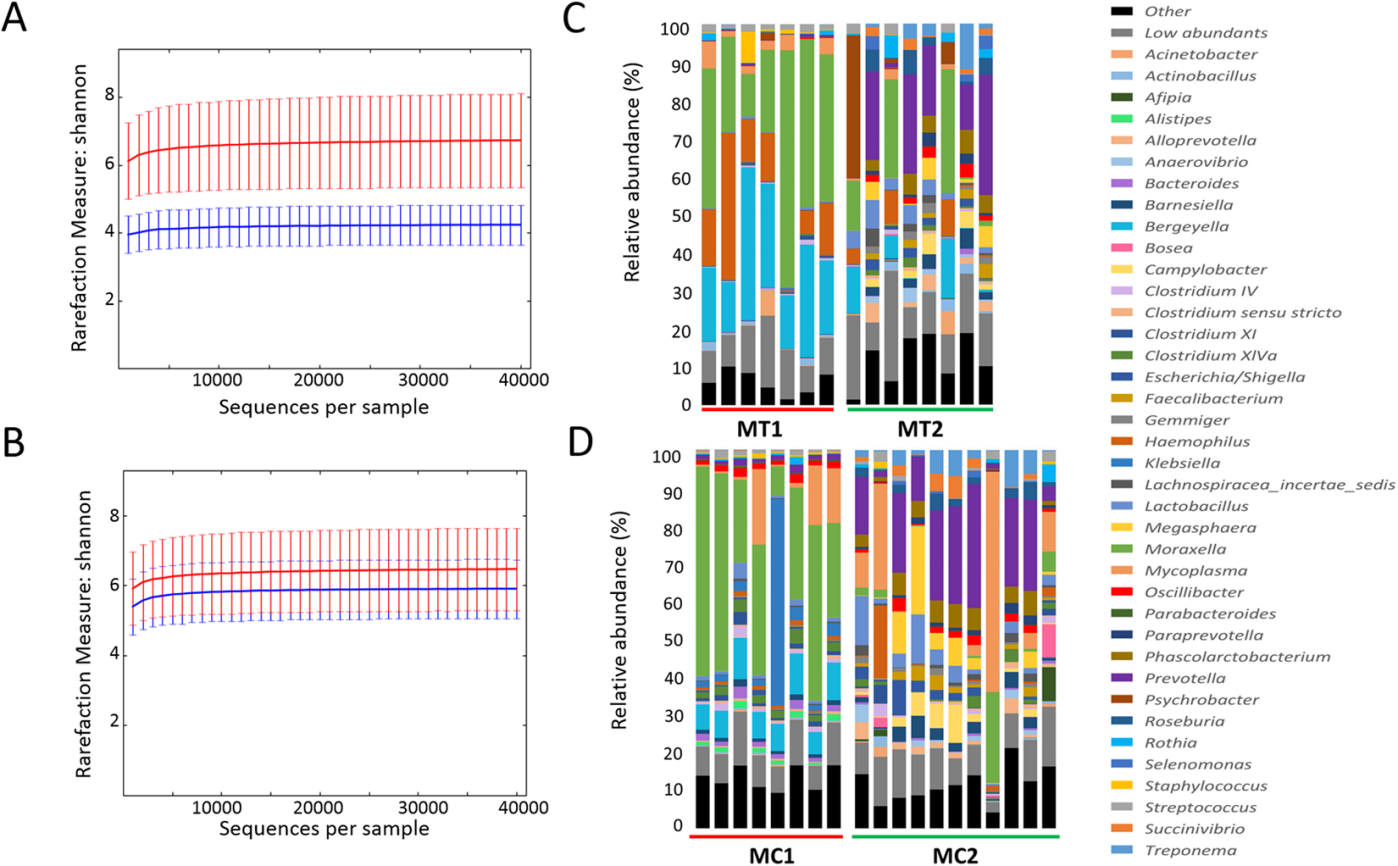
Nasal microbiota of piglets from farms MT and MC before (1) and after (2) antimicrobial treatment removal. Microbiota rarefaction curve generated using Shannon diversity estimator with samples from farms MT (**A**) and MC (**B**). Samples have been rarefied at an even depth of 40,000 sequences per sample. Error bars indicate the 95% confidence intervals. The mean relative abundance (%) of OTUs found in nasal swabs of piglets from farms MT (**C**) and MC (**D**) is presented at genera level.

With the aim of analysing the effect of perinatal antibiotic treatment on the nasal microbiota composition, 3–4 week old piglets from two farms (MT and MC farms) from Spain were sampled. Nasal swabs were taken when both farms were using perinatal antimicrobial treatments (MC1 and MT1), and total DNA was extracted and subjected to individual massive sequencing. The same sampling was repeated one productive cycle after elimination of perinatal antibiotics in both farms (MC2 and MT2). We obtained a total of 8,883,783 joint reads after filtering for both farms (MC1: 876,010; MC2: 3,178,022; MT1: 2,533,426; MT2: 2,296,325). The OTUs were identified in the samples that passed the quality-based filters (MT1, n = 7; MT2, n = 8; MC1, n = 8; MC2, n = 11) through clustering sequences at 97% sequence homology. At phylum level, 98.2% of the sequences were assigned to 7 phyla, while 93% of the sequences were assigned at family level, to 41 families. At genus level, 75.5% of the reads were assigned to 68 genera. The relative abundance of the OTUs found at genus level in the nasal microbiota is represented in Fig. 1 (for the full list of OTUs assigned at genera level, please refer to Additional File 1; see also Additional File 2 for other taxonomical levels).

One cycle after elimination of perinatal antimicrobials, nasal microbiota of all the weaning piglets analysed showed a significant increase in bacterial diversity, as indicated by the analysis of alpha diversity through Shannon diversity index (*P* = 0.002). When farms MC and MT were analysed individually (Fig. 1), this increase in alpha diversity was also observed, showing to be statistically significant through non-parametric two-sample t-test (999 permutations, P ≤ 0.05) only in farm MT (Fig. 1A,B). The mean of observed OTUs richness was also measured in rarefied samples at maximum depth (40,000) at the two sampling times in both farms, with means values of 1,465 (MC1) and 2,237 (MC2) OTUs for MC farm, and 1,221 (MT1) and 4,619 (MT2) OTUs for MT farm. The most predominant bacterial genera across all the samples at first sampling time was *Moraxella* for both farms (34.22 ± 6.30% for MT1 and 34.02 ± 5.79% for MC1, Fig. 1C,D) followed by *Bergeyella* (23.27 ± 3.6% for MT1 and 8.21 ± 0.7% for MC1, Fig. 1C,D), while the most abundant genera after antimicrobial elimination in both farms changed to *Prevotella* (14.06 ± 4.46% for MT2 and 16.81 ± 3.26% for MC2).

**Figure 2:**
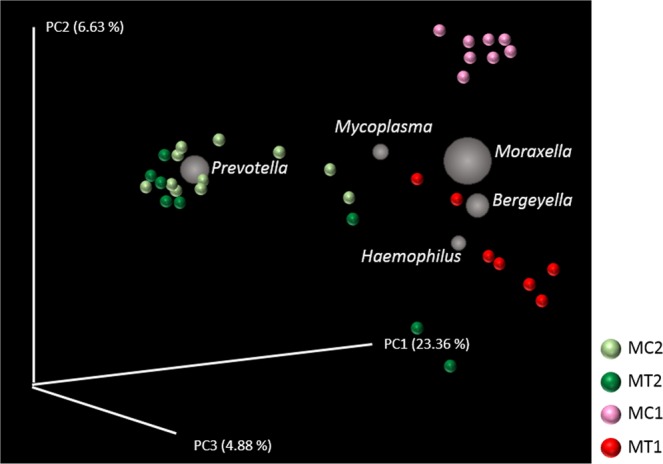
Principal Component Plots (jackknifed) representing beta diversity on rarefied samples. Beta diversity of nasal samples of piglets was computed through unweighted UniFrac analysis for both farms, MT and MC, in two different sampling times, before (1) and after (2) elimination of perinatal antibiotic treatment. The biplot shows grey spheres corresponding to the five most abundant genera visualized in the PCA space. The size of the spheres is proportional to their relative abundance across all the samples.

Analysis of the beta diversity both before and after elimination of antimicrobials, showed remarkable differences in the bacterial population of the nasal microbiota (Fig. 2). Antimicrobial treatment explained 22.63% of the differences observed (*P* = 0.001). The five most abundant genera explaining the divergence between the nasal microbiota before and after elimination of perinatal antimicrobials were: *Prevotella*, *Moraxella*, *Bergeyella*, *Haemophilus* and *Mycoplasma* (Fig. 2). When the beta diversity was analysed in each farm individually, significant differences were observed after elimination of the antimicrobial treatment in both farms (adonis, R^2^ = 0.2186, *P* = 0.001 for MC and R^2^ = 0.1935, *P* = 0.005 for MT). Elimination of antimicrobials caused significant changes in the relative abundance of several genera. Among others, *Lactobacillus, Phascolarctobacterium, Megasphaera, Roseburia* and *Campylobacter* were increased in both farms. The mean of the relative abundance of the *Prevotella* genus increased also after elimination of the antimicrobials, but this increase was only statistically significant for MC farm (Table 1). On the contrary, the relative abundance of *Moraxella* and *Bergeyella* significantly decreased, while *Haemophilus* and *Mycoplasma* showed a decreased tendency when antimicrobials were removed in farm MT, but increased in farm MC (Table 1). Finally, the relative abundance of *Neisseria* decreased after removal of antimicrobials, showing to be significant in MC farm (Table 1).

**Table 1:** Relative abundance of OTUs before and after elimination of antibiotic treatment in two different farms (>1% relative abundance).

OTU	Relative abundance (%)					
	MT1	MT2	*P value*	MC1	MC2	*P value*
*Acinetobacter*	1.090	0.835	0.132	<1	<1	NA
*Actinobacillus*	0.837	1.123	0.728	<1	<1	NA
*Afipia*	<1	<1	NA	0.000	1.089	0.001*
*Alistipes*	<1	<1	NA	1.242	0.006	<0.001*
*Alloprevotella*	0.048	1.902	0.064	0.444	1.635	0.021*
*Anaerovibrio*	0.011	1.009	0.037*	0.048	1.094	0.010*
*Bacteroides*	<1	<1	NA	1.694	0.366	<0.001*
*Barnesiella*	0.104	2.001	0.049*	1.172	2.562	0.160
*Bergeyella*	23.275	4.370	0.003*	8.215	0.009	<0.001*
*Bosea*	<1	<1	NA	0	1.153	<0.001*
*Campylobacter*	0.007	1.883	0.021*	0.030	2.882	0.001*
*Clostridium sensu stricto*	<1	<1	NA	1.207	0.736	0.039*
*Clostridium IV*	<1	<1	NA	2.473	1.283	0.017*
*Clostridium XI*	<1	<1	NA	1.341	0.723	0.017*
*Clostridium XlVa*	<1	<1	NA	2.416	1.195	0.017*
*Escherichia/Shigella*	0.100	1.174	0.418	0.528	2.212	0.058*
*Faecalibacterium*	0.026	1.411	0.011*	0.098	1.633	0.001*
*Gemmiger*	0.019	1.186	0.015*	<1	<1	NA
*Haemophilus*	13.896	2.927	0.015*	0.905	2.195	0.026*
*Klebsiella*	<1	<1	NA	8.810	0.011	<0.001*
*Lachnospiracea_incerta_sedis*	0.068	1.233	0.132	0.328	1.089	0.117
*Lactobacillus*	0.328	3.453	0.004*	1.400	4.406	0.032*
*Megasphaera*	0.010	2.164	0.008*	0.033	4.971	0.001*
*Moraxella*	34.220	9.185	0.015*	34.024	3.189	0.001*
*Mycoplasma*	3.051	0.577	0.011*	6.718	10.324	0.509
*Neisseria*	5.09	4.26	0.780	1.00	0.02	0.003*
*Oscillibacter*	0.119	1.426	0.165	1.586	1.483	0.680
*Paraprevotella*	0.010	1.067	0.028*	0.039	1.131	<0.001*
*Phascolarctobacterium*	0.043	3.013	0.004*	0.106	4.072	<0.001*
*Prevotella*	0.364	14.066	0.083	1.319	16.805	0.002*
*Psychrobacter*	0.276	5.518	0.563	<1	<1	NA
*Roseburia*	0.020	2.466	0.005*	0.080	1.972	0.001*
*Rothia*	0.558	1.406	0.817	<1	<1	NA
*Selenomonas*	0.007	1.113	0.028*	<1	<1	NA
*Staphylococcus*	1.493	0.029	0.002*	<1	<1	NA
*Streptococcus*	1.584	1.028	0.418	1.002	0.982	0.457
*Succinivibrio*	0.012	1.058	0.037*	0.083	1.706	0.137
*Treponema*	0.047	2.666	0.105	0.083	3.554	<0.001*

*Significantly different.

NA, not available. When the relative abundance was <1% in both sampling times, the *P value* was not estimated. The OTU was included for comparison with the other farm that showed higher abundance (>1%).

### Stability of microbiota composition after antimicrobial elimination

**Figure 3:**
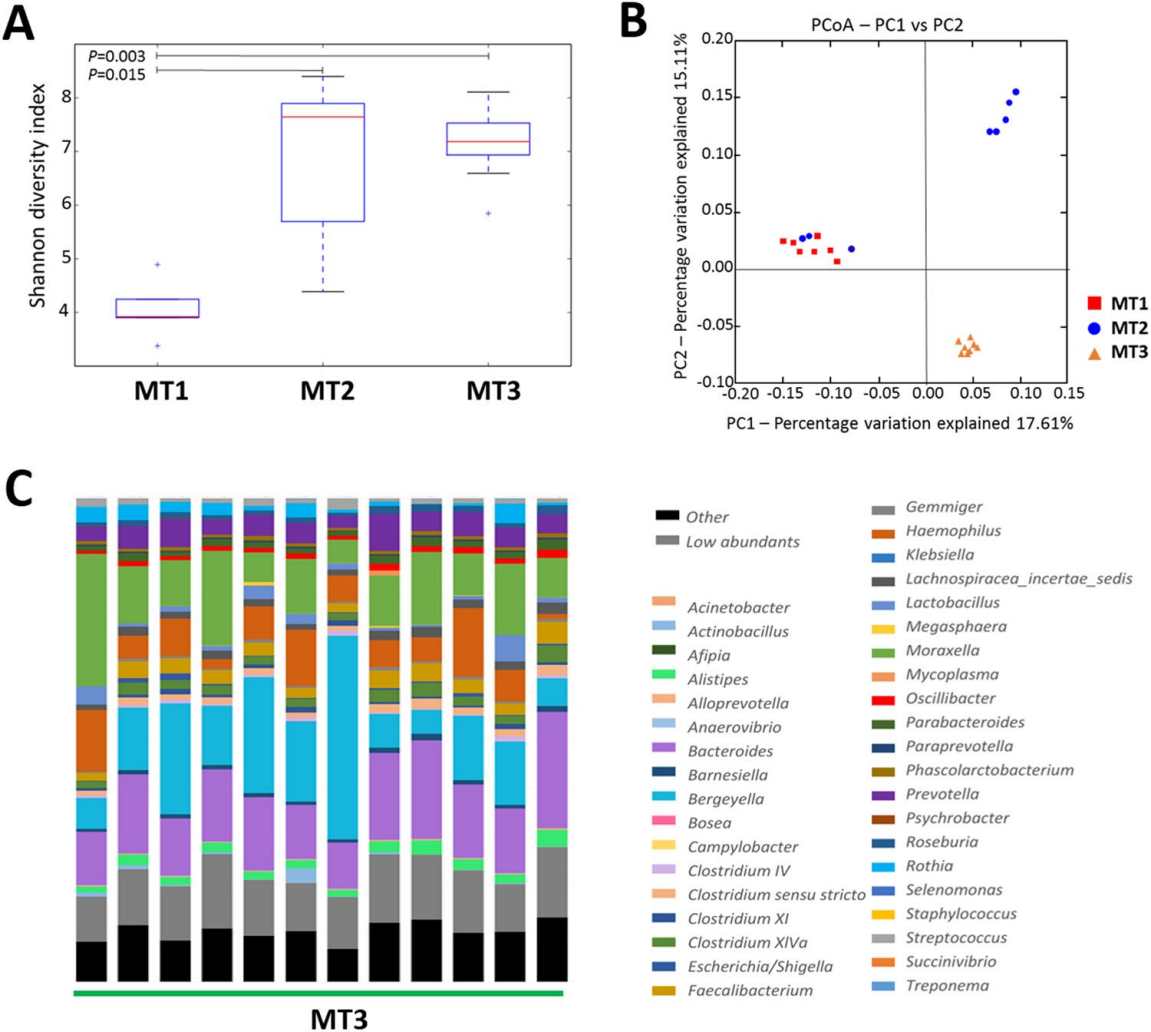
Stability of the nasal microbiota of piglets from farm MT through three different sampling time-points. (**A**) Alpha diversity using Shannon diversity index over rarefied samples is compared before perinatal antibiotic elimination (MT1) and after 1 (MT2) or 5 productive cycles (MT3). (**B**) Beta diversity analysis (jackknified principal component analysis) of the nasal microbiota composition among samples collected before (MT1) and after 1 (MT2) or 5 productive cycles (MT3) antimicrobial treatment removal. The two principal axis are shown with the percentage of variation explained between brackets. (**C**) The mean relative abundance (%) at genera level for farm MT after 5 productive cycles without using perinatal antibiotic treatment. Color-coding for each representative OTU are the same that in Fig. 1.

In order to evaluate the stability of these changes in the nasal microbiota composition, a third sampling was performed in farm MT (MT3). In this run, we obtained a total of 4,003,077 joint reads after filtering (MT3, n = 12). Analysis of alpha diversity showed no further increase in diversity after additional time without perinatal antimicrobials in the farm (Fig. 3A, for a full list of OTUs assigned at genera level, please see Additional File 3). However, the samples after 5 cycles without antimicrobials showed a more similar composition demonstrated with the lower *P* value obtained through the comparison (Fig. 3A; Shannon index, *P* = 0.003). Besides, the beta diversity analysis, showed a distinct clustering at the three sampling times tested (Fig. 3B, adonis R^2^ = 0.15643, *P* < 0.001). Sixteen genera were involved in the long-term changes observed after elimination of the antimicrobials (Table 2 and Fig. 3C). Nine of these genera were increased at the third sampling point after 5 productive cycles without antimicrobials, such as *Faecaliabacterium* and *Bacteroides*.

**Table 2:** Relative abundance of OTUs at third sampling time in MT farm compared with the previous samplings.

OTU	Relative abundance MT3 (%)	*MT1 vs MT3 P value*	*MT2 vs MT3 P value*
*Acinetobacter*	0.013^#^	0.002*	NA
*Actinobacillus*	0.521^#^	NA	0.482
*Alistipes*	2.043	0.002*	<0.001*
*Alloprevotella*	0.222^#^	NA	0.354
*Anaerovibrio*	0.044^#^	NA	0.165
*Bacteroides*	15.104	0.002*	<0.001*
*Barnesiella*	0.895^#^	NA	0.354
*Bergeyella*	15.109	0.09	0.011*
*Campylobacter*	0.023^#^	NA	0.105
*Clostridium sensu stricto*	1.452	0.527	0.217
*Clostridium XlVa*	2.064	0.002*	0.007*
*Escherichia/Shigella*	0.431^#^	NA	0.938
*Faecalibacterium*	2.903	0.002*	0.017*
*Gemmiger*	0.479^#^	NA	0.354
*Haemophilus*	6.954	0.343	0.031*
*Lachnospiracea_incertae_sedis*	1.665	0.002*	0.217
*Lactobacillus*	1.751	0.008*	0.076
*Megasphaera*	0.130^#^	NA	0.076
*Moraxella*	12.308	0.015*	0.189
*Mycoplasma*	0.086^#^	0.002*	NA
*Oscillibacter*	1.067	0.002*	0.487
*Parabacteroides*	1.386	0.002*	<0.001*
*Paraprevotella*	0.301^#^	NA	0.354
*Phascolarctobacterium*	0.733^#^	NA	0.354
*Prevotella*	4.347	0.002*	0.354
*Psychrobacter*	0.027^#^	NA	0.537
*Roseburia*	1.181	0.002*	0.938
*Rothia*	1.838	0.002*	0.105
*Selenomonas*	0.009^#^	NA	0.013*
*Staphylococcus*	0.037^#^	0.002*	NA
*Streptococcus*	1.023	0.035*	0.354
*Succinivibrio*	0.029^#^	NA	0.105
*Treponema*	0.074^#^	NA	0.142

*Significantly different.

^#^The OTU is included in the list although having relative abundance less than 1% for comparison with previous samplings.

NA, not available. When OTUs appeared at less than 1% of relative abundance in both sampling times, the *P value* was not estimated.

### Health and production parameters in the nursery phase

Concomitantly to the increase in microbiota diversity, the MT farm experienced an improvement of health and productivity. Analysis of production parameters in the nursery phase from 2015 to 2017, indicated that the elimination of antimicrobial treatments resulted in a significant reduction in medication cost by pig and mortality rate, while the reduction observed in feed conversion ratio (FCR) was not statistically significant (*P* = 0.110) (Table 3). In farm MC, the FCR (*P* = 0.261) did not show a clear improvement, but the mortality rate (*P* = 0.130) and especially the cost in medication per pig (*P* = 0.05) decreased during the study period.

**Table 3:** Production data collected from the nursery phases of MT and MC farms in three consecutive years.

	MT			*P* value*	MC			*P* value*
	2015	2016	2017		2015	2016	2017	
Medication cost (€/pig)	0.870	0.543	0.515	0.037	0.843	0.478	0.390	0.050
Mortality rate (%)	5.46	3.24	3.19	0.050	2.83	3.05	2.58	0.130
Feed conversion ratio	1.659	1.487	1.477	0.110	1.675	1.471	1.559	0.261

*P values were estimated through Krukal-Wallis non-parametric test.

## Discussion

The effect of perinatal antimicrobial treatments in the gut microbiota of piglets has been already documented^11,12^, but less information is available on the effect of these treatments on the respiratory tract microbiota. In our study, elimination of perinatal antimicrobials resulted in an increase in nasal microbiota diversity, which was accompanied by a general health improvement in the piglets. This observation is in agreement with previous reports showing an association between health and microbiota diversity^10,13^. Although not addressed here, another additional benefit expected after elimination of metaphylactic antimicrobial treatments, would be the reduction in the abundance of resistance genes, as reported in the pig GIT microbiome^10^. Worth to mention is that a more similar microbiota composition among the individuals was found in the piglets after 5 cycles without using antimicrobials, indicating a developed stability in the microbiota composition. On the contrary, dissimilarity in microbiota composition within groups has been considered a measurement of gut dysbiosis associated with poor health status^14^. Importantly, a lower relative abundance of several potentially pathogenic bacteria in the nasal microbiota was also demonstrated, while beneficial bacteria were more abundant in both farms when no perinatal antimicrobial treatment was applied. Our results agree with those reported by Janczyk *et al*. (2007) for the impact of amoxicillin on the intestinal microbiota of piglets^12^. Higher relative abundance of potential pathogens and a reduction of beneficial bacteria, such as *Lactobacillus*, was observed when long-lasting amoxicillin was administered to piglets, although no direct connection with health status was reported^12^. Recently, the effect of antimicrobial administration was evaluated in older pigs (8 week old) under experimental conditions, where no differences were found on bacterial diversity^15^. Apparent incongruences with that study could be explained considering the age of the animals, as it was reported that the nasal microbiota reaches stability 2–3 weeks after weaning^16^. Thus, the effect of antimicrobial treatment is not expected to be the same in adult animals with an established nasal microbiota, than in new-born piglets where the microbiota composition is still developing and can be more easily influenced.

Under farm conditions, the use of perinatal antimicrobials has been reported to be detrimental to the health of piglets, which may be linked to the higher abundance of potential pathogens^17^. Although we cannot rule out that a reduced diversity caused a poorer development of the immune system^11^, the healthier condition observed in the present study can be associated to the decreased colonization of the upper respiratory tract by potential pathogens, which may be farm specific in some cases (e.g., *Mycoplasma* or *Haemophilus*). Nasal colonization of piglets by *Mycoplasma hyopneumoniae* at weaning has been associated to a higher risk of disease development in growing pigs^18^. Additionally, *M. hyopneumoniae* plays a role in the porcine respiratory disease complex and has been associated to increase disease severity^19^. Some potential pathogens, such as *Haemophilus parasuis*, are highly heterogeneous, and while the presence of virulent strains in the nasal cavity of piglets can be considered a risk factor for disease development, the presence of non-virulent strains could confer protection against disease^20^. In other cases, the role of a genus in disease is less clear. The genus *Moraxella* is highly abundant in the nasal microbiota of healthy pigs^6^, but some *Moraxella* species have been isolated from systemic lesions, indicating that they have pathogenic potential^21–23^. Although there is no available confirmation that *Bergeyella* can be pathogenic to pigs, some species has been associated to human septicaemia and endocarditis^24^. Experimental support to the putative pathogenicity of *Bergeyella* in pigs has been obtained in *in vitro* studies with nasal isolates from this bacterial genus^25^. The reduction in the abundance of pathogens reported may be the result of direct competition of the microbiota against pathogen colonization or indirectly by an improved maturation of the immune system^11^. Either way, the resulting reduction in pathogen load would most probably result in an improved-health status. On the other hand, the relative abundance of some potential pathogens was increased, as observed for *Campylobacter* and *Treponema*. However, the role of these genera in disease needs deeper analysis at species and strain level to proper assess their virulence potential. For instance, *Campylobacter* comprises a high diversity of strains, which are becoming apparent by studying *Campylobacter* populations in different animal species^26^.

The balance in the composition of the microbiota community needs to be also considered. Thus, while the abundance of some pathogenic bacterial genera decreased, the opposite was observed for some of the genera linked to beneficial effects on animal health. Besides the well-known probiotic genus *Lactobacillus*, other potential beneficial genera were detected in this study, such as *Prevotella* and *Bacteroides*. *Prevotella* has been reported previously as a beneficial and/or protective bacteria or at least an important component of healthy microbiota of animals and humans^27,28^. Although some *Bacteroides* have been considered opportunist pathogens^29–31^, at least one strain within the species *Bacteroides fragilis* has been identified as a potential probiotic for human health^32^. In general, an in-depth analysis of the specific species and strains is needed to define the specific role of each taxon within each genus and its potential role in animal health.

The present study confirms the benefits of the elimination of perinatal antimicrobial usage in the health of piglets, through modulation of the nasal microbiota composition. Based on the long-term results presented herein, antimicrobial treatments should be carefully applied especially at early stages in life, when the cross-talk with commensal microorganisms will determine the microbiota establishment throughout life.

## Material and Methods

### Farms and sampling

Nasal swabs from 3–4 week old piglets were collected at different time points, from two different farms (MT and MC) located in the area of Catalonia, Spain. First sampling was done on May-June 2015 when both farms were using perinatal antimicrobial treatments, labelled as MT1 and MC1, for MT and MC farms respectively. MT farm used penicillin and streptomycin at 3 days after birth and tulatromycin one week later; while MC farm used ceftiofur at 3 days and tulatromycin one week later. A second sampling was done after one farrowing interval without usage of any perinatal antimicrobial treatment, in October-November 2015, labelled as MT2 and MC2. In MT farm, a third sampling was performed in March 2017 (labelled as MT3). The MT farm was a 3300-sows multi-site pig production system and MC farm was a 480-sows farrow-to-finish production system. MC farm was negative to porcine reproductive and respiratory syndrome virus (PRRSV) while MT was PRRS-positive, although no circulation of the virus was diagnosed throughout the study. All the animals were vaccinated against porcine circovirus type 2 (PCV2) and *Mycoplasma hyopneumoniae*. During the study period no other management practices were modified in any farm, except for the antibiotic treatment elimination. Production data were collected during the period of the study. Collection of nasal swabs (sterile with aluminium handle) was done one day prior weaning from both nostrils (5–6 cm depth) using the same swab. The swabs were kept refrigerated until arrival within 24 hours to the laboratory, where they were stored in PBS (0.5 mL) at −20 °C.

Sampling of piglets was done under institutional authorization and followed good veterinary practices. According to European (Directive 2010/63/EU of the European Parliament and of the Council of 22 September 2010 on the protection of animals used for scientific purposes) and Spanish (Real Decreto 53/2013) normative, this procedure did not require specific approval by an Ethical Committee. Nasal sampling was performed only once to each piglet and is not likely to cause pain, suffering, distress or lasting harm equivalent to, or higher than, that caused by the introduction of a needle in accordance with good veterinary practice (Chapter I, Article 1, 5 (f) of 2010/63/EU).

### DNA extraction and sequencing

Total genomic DNA was extracted using Machinery Nigel Kit (GmbH & Co, Düren; Germany), following manufacter’s instructions and resuspended in 50 µl of elution buffer. Quality and quantity was evaluated on a BioDrop DUO (BioDrop Ltd, Cambridge. UK). The library preparation for sequencing was performed within 24 h after the DNA extraction at *Servei de Genòmica, Universitat Autònoma de Barcelona*. Sequencing of the V3-V4 region of 16S rDNA gene was done with Illumina MiSeq pair-end 2 × 250 bp technology following the manufacturer instructions (MS-102–2003 MiSeq® Reagent Kit v2, 500 cycle). The region targeted to perform the 16S amplification was the one spanning the V3 and V4 region of 16S rRNA gene selected from Klindworth *et al*.^33^. Interest-specific primers targeting this region were the ones recommended by Illumina with overhang adapters attached:

16S Forward Primer 5′ TCGTCGGCAGCGTCAGATGTGTATAAGAGACAGCCTACGGGNGGCWGCAG

16S Reverse Primer 5′ GTCTCGTGGGCTCGGAGATGTGTATAAGAGACAGGACTACHVGGGTATCTAATCC.

Cycling conditions were the same reported previously^6^. The PCR products were purified and checked to verify its size on a Bioanalyzer DNA 1000 chip (Agilent).

### Read filtering and data analysis

For the taxonomic analysis, reads were quality-filtered (Q > 25) before paired-end joining by QIIME v1.9 software^34^, using fastq join^35,36^ under default values. Sequences were clustered into OTUs at 97% similarity using UCLUST algorithm^37^ and the Greengenes database^38^. Chimeric detection and removal was done with USEARCH 6.1^37,39^ against the ChimeraSlayer reference database^40^. To minimize the inflation of rare OTUs in the community analysis, we also exclude singletons for further processing. Taxonomic assignment was done Naïve Bayes classification against RDP database^41^. For each taxon, the Kruskal Wallis test was perform to compare OTU frequencies in sample groups and to ascertain whether or not there are statistically significant differences between the OTU abundance in the different sample groups, *P* values were FDR-corrected for multiple hypotheses testing. Single rarefaction, based on the sample with the lowest number of reads, was used for alpha-diversity analysis. Diversity indexes were calculated on rarefied 16S rRNA gene sequence data for all samples at 97% similarity. Alpha diversity between groups was compared through two-sample non-parametric t-tests (Monte Carlo method) at maximum depth in rarefied samples (with 999 permutations). In addition, equal number of samples was subsampled to assess the significant differences between sample types using UniFrac weighted and unweighted distances^42,43^. The percentage of variation between grouped samples was measured by R^2^, using adonis function of the vegan package^44^ in R software. Estimation of *P* values was done through Monte Carlo test with 999 random permutations of the data set. Preliminarily, combined data from both farms were compared before and after antimicrobial treatment elimination to explore the data. However, each farm was analysed separately at the different time points throughout all the study. Samples were considered to be significantly different when the accompanying *P* value was ≤0.05. Results were confirmed with SAS software through PROC GLM analysis.

The production data parameters were statistically analysed through Krukal-Wallis non-parametric test using R software^45^.

## Supplementary information

Supplementary info

## Data Availability

The entire sequence dataset is available at the NCBI database, SRA accession PRJNA495126.
