# Supplementary material for: Antimicrobial removal on piglets promotes health and higher bacterial diversity in the nasal microbiota
Source: Sci Rep. 2019 Apr 25;9:6545. doi: 10.1038/s41598-019-43022-y (PMC6484018; doi:10.1038/s41598-019-43022-y)
Supplement: Supplementary file 1 — Supplementary info [file 41598_2019_43022_MOESM1_ESM.pdf]

Antimicrobial removal on piglets promotes health and higher bacterial diversity in the nasal microbiota

Florencia Correa-Fiz[1,*], José Maurício Gonçalves dos Santos[2], Francesc Illas[3], Virginia Aragon[1]

[1] IRTA, Centre de Recerca en Sanitat Animal (CReSA, IRTA-UAB), Campus de la Universitat Autònoma de Barcelona, 08193 Bellaterra, Spain

[2] Unicesumar-Centro Universitário de Maringá, Maringá, Paraná, Brazil

[3] Selección Batallé. Avinguda dels segadors. 17421 Riudarenes, Spain

*Corresponding author: Florencia Correa-Fiz

email: flor.correa@irta.cat

José Maurício Gonçalves dos Santos

email: jmgds@hotmail.com

Francesc Illas

email: fillas@batalle.com

Virginia Aragon

email: virginia.aragon@irta.cat

Additional file 1A. Frequencies of OTUs assigned at genus level from farm MT at the two sampling times (MT1 and MT2).

OTU	Phylum	Class	Order	Family	Genus	Frquency		FDR_P
						MT1	MT2	
Bacteria	*Firmicutes*	*Bacilli*	*Lactobacillales*	*Aerococcaceae*	*Abiotrophia*	1,46812E-05	0	0,117601295
Bacteria	*Firmicutes*	*Clostridia*	*Clostridiales*	*Ruminococcaceae*	*Acetanaerobacterium*	9,19829E-05	0,002419222	0,020524464
Bacteria	*Firmicutes*	*Clostridia*	*Clostridiales*	*Lachnospiraceae*	*Acetitomaculum*	0	7,84276E-06	0,037588881
Bacteria	*Firmicutes*	*Clostridia*	*Clostridiales*	*Ruminococcaceae*	*Acetivibrio*	1,44989E-05	0,000160301	0,03646983
Bacteria	*Firmicutes*	*Clostridia*	*Clostridiales*	*Peptostreptococcaceae*	*Acetoanaerobium*	0	1,19968E-06	0,349574806
Bacteria	*Proteobacteria*	*Alphaproteobacteria*	*Rhodospirillales*	*Acetobacteraceae*	*Acetobacter*	0	0,000037	0,082355525
Bacteria	*Tenericutes*	*Mollicutes*	*Acholeplasmatales*	*Acholeplasmataceae*	*Acholeplasma*	0,00025045	9,1606E-06	0,65591049
Bacteria	*Proteobacteria*	*Betaproteobacteria*	*Burkholderiales*	*Alcaligenaceae*	*Achromobacter*	0	2,99919E-06	0,349574806
Bacteria	*Firmicutes*	*Clostridia*	*Clostridiales*	*Clostridiales_Incertae Sedis XII*	*Acidaminobacter*	0	5,87721E-07	0,349574806
Bacteria	*Firmicutes*	*Negativicutes*	*Selenomonadales*	*Acidaminococcaceae*	*Acidaminococcus*	1,37351E-05	0,000732089	0,014276949
Bacteria	*Proteobacteria*	*Alphaproteobacteria*	*Rhodospirillales*	*Acetobacteraceae*	*Acidicaldus*	3,94286E-06	0	0,285049407
Bacteria	*Proteobacteria*	*Betaproteobacteria*	*Burkholderiales*	*Comamonadaceae*	*Acidovorax*	3,50182E-05	1,23301E-05	0,945071382
Bacteria	*Proteobacteria*	*Gammaproteobacteria*	*Pseudomonadales*	*Moraxellaceae*	*Acinetobacter*	0,010899768	0,00834828	0,132463567
Bacteria	*Proteobacteria*	*Gammaproteobacteria*	*Pasteurellales*	*Pasteurellaceae*	*Actinobacillus*	0,005321002	0,008967238	0,728454189
Bacteria	*Actinobacteria*	*Actinobacteria*	*Actinomycetales*	*Actinomycetaceae*	*Actinobaculum*	5,29732E-06	8,99757E-07	0,362848315

Bacteria	*Actinobacteria*	*Actinobacteria*	*Actinomycetales*	*Actinomycetaceae*	*Actinomyces*	6,98171E-05	0,000162829	0,035557906
Bacteria	*Actinobacteria*	*Actinobacteria*	*Actinomycetales*	*Micromonosporaceae*	*Actinoplanes*	7,80857E-06	0	0,285049407
Bacteria	*Actinobacteria*	*Actinobacteria*	*Coriobacteriales*	*Coriobacteriaceae*	*Adlercreutzia*	0,000000862	2,22611E-06	0,619656703
Bacteria	*Proteobacteria*	*Betaproteobacteria*	*Burkholderiales*	*Alcaligenaceae*	*Advenella*	0,000100444	3,25633E-05	0,55568979
Bacteria	*Firmicutes*	*Bacilli*	*Bacillales*	*Bacillaceae 1*	*Aeribacillus*	1,41306E-06	0	0,285049407
Bacteria	*Firmicutes*	*Bacilli*	*Lactobacillales*	*Aerococcaceae*	*Aerococcus*	0,001004635	0,001634028	0,104881801
Bacteria	*Proteobacteria*	*Gammaproteobacteria*	*Aeromonadales*	*Aeromonadaceae*	*Aeromonas*	7,18931E-05	1,14766E-05	0,148871993
Bacteria	*Proteobacteria*	*Alphaproteobacteria*	*Rhizobiales*	*Bradyrhizobiaceae*	*Afipia*	8,50657E-06	0,000725663	0,033894854
Bacteria	*Actinobacteria*	*Actinobacteria*	*Actinomycetales*	*Microbacteriaceae*	*Agrococcus*	7,06532E-07	0	0,285049407
Bacteria	*Verrucomicrobia*	*Verrucomicrobiae*	*Verrucomicrobiales*	*Verrucomicrobiaceae*	*Akkermansia*	1,98358E-05	0,000237814	0,040997061
Bacteria	*Proteobacteria*	*Betaproteobacteria*	*Burkholderiales*	*Comamonadaceae*	*Albidiferax*	0	1,48386E-06	0,349574806
Bacteria	*Proteobacteria*	*Betaproteobacteria*	*Burkholderiales*	*Alcaligenaceae*	*Alcaligenes*	5,36476E-05	2,91662E-07	0,013115904
Bacteria	*Proteobacteria*	*Gammaproteobacteria*	*Alteromonadales*	*Alteromonadaceae*	*Alishewanella*	0,000001393	4,79871E-06	1
Bacteria	*Bacteroidetes*	*Bacteroidia*	*Bacteroidales*	*Rikenellaceae*	*Alistipes*	0,000157418	1,40027E-05	0,002183045
Bacteria	*Bacteroidetes*	*Bacteroidia*	*Bacteroidales*	*Marinilabiaceae*	*Alkaliflexus*	0	4,19887E-06	0,349574806
Bacteria	*Bacteroidetes*	*Bacteroidia*	*Bacteroidales*	*Marinilabiliaceae*	*Alkalitalea*	0	4,73698E-07	0,349574806
Bacteria	*Proteobacteria*	*Gammaproteobacteria*	*Pseudomonadales*	*Moraxellaceae*	*Alkanindiges*	2,43212E-05	6,25013E-06	0,695298567
Bacteria	*Firmicutes*	*Negativicutes*	*Selenomonadales*	*Veillonellaceae*	*Allisonella*	7,17768E-06	0,000378233	0,020462223

Bacteria	*Firmicutes*	*Erysipelotrichia*	*Erysipelotrichales*	*Erysipelotrichaceae*	*Allobaculum*	0,000000862	4,42176E-07	0,844911363
Bacteria	*Firmicutes*	*Bacilli*	*Lactobacillales*	*Carnobacteriaceae*	*Alloiococcus*	1,97927E-05	0,000363317	0,79400268
Bacteria	*Proteobacteria*	*Gammaproteobacteria*	*Vibrionales*	*Vibrionaceae*	*Allomonas*	3,86271E-05	0	0,117601295
Bacteria	*Bacteroidetes*	*Bacteroidia*	*Bacteroidales*	*Prevotellaceae*	*Alloprevotella*	0,00048278	0,018995671	0,064077507
Bacteria	*Proteobacteria*	*Betaproteobacteria*	*Neisseriales*	*Neisseriaceae*	*Alysiella*	0,000533679	3,99027E-05	0,706491451
Bacteria	*Proteobacteria*	*Alphaproteobacteria*	*Rhodobacterales*	*Rhodobacteraceae*	*Amaricoccus*	1,66233E-05	0	0,117601295
Bacteria	*Firmicutes*	*Clostridia*	*Clostridiales*	*Clostridiaceae 1*	*Anaerobacter*	1,90093E-05	5,64288E-05	0,082579274
Bacteria	*Proteobacteria*	*Gammaproteobacteria*	*Aeromonadales*	*Succinivibrionaceae*	*Anaerobiospirillum*	0	7,36337E-05	0,037588881
Bacteria	*Firmicutes*	*Clostridia*	*Clostridiales*	*Clostridiales_Incertae Sedis XI*	*Anaerococcus*	0,000135921	0,000346854	0,813663716
Bacteria	*Firmicutes*	*Clostridia*	*Clostridiales*	*Ruminococcaceae*	*Anaerofilum*	1,84716E-05	2,25993E-05	0,473289465
Bacteria	*Firmicutes*	*Clostridia*	*Clostridiales*	*Eubacteriaceae*	*Anaerofustis*	0	3,78847E-06	0,037588881
Bacteria	*Firmicutes*	*Negativicutes*	*Selenomonadales*	*Veillonellaceae*	*Anaeroglobus*	0	1,97729E-06	0,082355525
Bacteria	*Proteobacteria*	*Deltaproteobacteria*	*Myxococcales*	*Cystobacteraceae*	*Anaeromyxobacter*	0	1,8518E-06	0,349574806
Bacteria	*Bacteroidetes*	*Bacteroidia*	*Bacteroidales*	*Marinilabiaceae*	*Anaerophaga*	0,000179729	0,003246126	0,036566184
Bacteria	*Tenericutes*	*Mollicutes*	*Anaeroplasmatales*	*Anaeroplasmataceae*	*Anaeroplasma*	5,59218E-05	0,00015883	0,765087193
Bacteria	*Bacteroidetes*	*Bacteroidia*	*Bacteroidales*	*Bacteroidaceae*	*Anaerorhabdus*	0	2,24169E-06	0,17090352
Bacteria	*Firmicutes*	*Clostridia*	*Clostridiales*	*Incertae Sedis XI*	*Anaerosphaera*	8,75623E-06	4,28835E-06	0,740726595
Bacteria	*Firmicutes*	*Clostridia*	*Clostridiales*	*Clostridiaceae 1*	*Anaerosporobacter*	4,77613E-06	0,000100381	0,007280692

Bacteria	*Firmicutes*	*Clostridia*	*Clostridiales*	*Lachnospiraceae*	*Anaerostipes*	6,89563E-05	0,000153987	1
Bacteria	*Firmicutes*	*Clostridia*	*Clostridiales*	*Ruminococcaceae*	*Anaerotruncus*	9,9237E-06	5,35615E-05	0,094264307
Bacteria	*Firmicutes*	*Negativicutes*	*Selenomonadales*	*Veillonellaceae*	*Anaerovibrio*	0,000112203	0,010087487	0,037242545
Bacteria	*Firmicutes*	*Clostridia*	*Clostridiales*	*Clostridiales_Incertae Sedis XIII*	*Anaerovorax*	2,32093E-05	0,000531362	0,003813532
Bacteria	*Firmicutes*	*Bacilli*	*Bacillales*	*Bacillaceae 1*	*Anoxybacillus*	8,76192E-07	0	0,285049407
Bacteria	*Proteobacteria*	*Betaproteobacteria*	*Burkholderiales*	*Burkholderiales_incertae_sedis*	*Aquabacterium*	2,17767E-05	7,42095E-07	0,334764268
Bacteria	*Proteobacteria*	*Alphaproteobacteria*	*Rhizobiales*	*Phyllobacteriaceae*	*Aquamicrobium*	1,41546E-05	2,09943E-06	0,456570763
Bacteria	*Actinobacteria*	*Actinobacteria*	*Actinomycetales*	*Actinomycetaceae*	*Arcanobacterium*	8,15922E-06	0,000262903	0,372857936
Bacteria	*Proteobacteria*	*Epsilonproteobacteria*	*Campylobacterales*	*Campylobacteraceae*	*Arcobacter*	0,003726715	0,000177386	0,48352206
Bacteria	*Armatimonadetes*	*Armatimonadetes_gp5*	*Armatimonadetes_gp5*	*Armatimonadetes_gp5*	*Armatimonadetes_gp5*	5,14343E-05	0	0,017484674
Bacteria	*Actinobacteria*	*Actinobacteria*	*Actinomycetales*	*Micrococcaceae*	*Arthrobacter*	6,80457E-05	2,37748E-05	0,143234908
Bacteria	*Tenericutes*	*Mollicutes*	*Anaeroplasmatales*	*Anaeroplasmataceae*	*Asteroleplasma*	0,000024146	0,001211365	0,055829295
Bacteria	*Proteobacteria*	*Alphaproteobacteria*	*Caulobacterales*	*Caulobacteraceae*	*Asticcacaulis*	3,02417E-06	7,86281E-07	0,766432717
Bacteria	*Firmicutes*	*Bacilli*	*Lactobacillales*	*Carnobacteriaceae*	*Atopobacter*	0,00000362	3,22388E-06	0,900108638
Bacteria	*Actinobacteria*	*Actinobacteria*	*Coriobacteriales*	*Coriobacteriaceae*	*Atopobium*	5,44437E-06	3,25256E-06	0,740726595
Bacteria	*Firmicutes*	*Bacilli*	*Lactobacillales*	*Carnobacteriaceae*	*Atopostipes*	5,03444E-05	0	0,047105791
Bacteria	*Proteobacteria*	*Alphaproteobacteria*	*Rhizobiales*	*Aurantimonadaceae*	*Aurantimonas*	1,22498E-05	0	0,285049407
Bacteria	*Proteobacteria*	*Betaproteobacteria*	*Rhodocyclales*	*Rhodocyclaceae*	*Azoarcus*	0	0,000003599	0,349574806

Bacteria	*Proteobacteria*	*Gammaproteobacteria*	*Pseudomonadales*	*Pseudomonadaceae*	*Azomonas*	0,000006042	0	0,047105791
Bacteria	*Proteobacteria*	*Gammaproteobacteria*	*Pseudomonadales*	*Pseudomonadaceae*	*Azorhizophilus*	4,77613E-06	0	0,285049407
Bacteria	*Firmicutes*	*Bacilli*	*Bacillales*	*Bacillaceae 1*	*Bacillus*	0,000289558	2,48493E-05	0,010614291
Bacteria	*Bacteroidetes*	*Bacteroidia*	*Bacteroidales*	*Bacteroidaceae*	*Bacteroides*	0,001139096	0,002438111	0,417886964
Bacteria	*Bacteroidetes*	*Bacteroidia*	*Bacteroidales*	*Porphyromonadaceae*	*Barnesiella*	0,001050761	0,020044029	0,049140834
Bacteria	*Proteobacteria*	*Gammaproteobacteria*	*Pasteurellales*	*Pasteurellaceae*	*Basfia*	0	4,42176E-07	0,349574806
Bacteria	*Firmicutes*	*Bacilli*	*Lactobacillales*	*Enterococcaceae*	*Bavariicoccus*	1,87951E-05	4,3619E-06	0,65591049
Bacteria	*Proteobacteria*	*Betaproteobacteria*	*Neisseriales*	*Neisseriaceae*	*Bergeriella*	3,62737E-05	5,28418E-05	0,562484662
Bacteria	*Bacteroidetes*	*Flavobacteriia*	*Flavobacteriales*	*Flavobacteriaceae*	*Bergeyella*	0,232747015	0,043696028	0,002621822
Bacteria	*Proteobacteria*	*Gammaproteobacteria*	*Pasteurellales*	*Pasteurellaceae*	*Bibersteinia*	7,00205E-06	0,000028771	0,293270727
Bacteria	*Actinobacteria*	*Actinobacteria*	*Bifidobacteriales*	*Bifidobacteriaceae*	*Bifidobacterium*	6,95368E-06	6,36513E-05	0,067277961
Bacteria	*Proteobacteria*	*Deltaproteobacteria*	*Desulfovibrionales*	*Desulfovibrionaceae*	*Bilophila*	1,22731E-05	0,000194952	0,020462223
Bacteria	*Planctomycetes*	*Planctomycetia*	*Planctomycetales*	*Planctomycetaceae*	*Blastopirellula*	0	1,0299E-06	0,17090352
Bacteria	*Firmicutes*	*Clostridia*	*Clostridiales*	*Lachnospiraceae*	*Blautia*	0,000272265	0,005602877	0,082579274
Bacteria	*Proteobacteria*	*Betaproteobacteria*	*Burkholderiales*	*Alcaligenaceae*	*Bordetella*	7,76121E-06	6,14968E-06	0,740726595
Bacteria	*Proteobacteria*	*Alphaproteobacteria*	*Rhizobiales*	*Bradyrhizobiaceae*	*Bosea*	3,06667E-06	0,000858209	0,005020543
Bacteria	*Actinobacteria*	*Actinobacteria*	*Actinomycetales*	*Dermabacteraceae*	*Brachybacterium*	0,000116245	0,000210955	0,726095162
Bacteria	*Proteobacteria*	*Betaproteobacteria*	*Burkholderiales*	*Comamonadaceae*	*Brachymonas*	6,28151E-06	3,72161E-05	0,881930721

Bacteria	*Spirochaetes*	*Spirochaetia*	*Spirochaetales*	*Brachyspiraceae*	*Brachyspira*	0	9,25898E-07	0,349574806
Bacteria	*Proteobacteria*	*Alphaproteobacteria*	*Rhizobiales*	*Bradyrhizobiaceae*	*Bradyrhizobium*	0	1,46713E-05	0,037588881
Bacteria	*Actinobacteria*	*Actinobacteria*	*Actinomycetales*	*Brevibacteriaceae*	*Brevibacterium*	0,000125586	3,89682E-05	0,013328329
Bacteria	*Proteobacteria*	*Alphaproteobacteria*	*Caulobacterales*	*Caulobacteraceae*	*Brevundimonas*	0,000634013	7,50335E-06	0,000967429
Bacteria	*Firmicutes*	*Bacilli*	*Bacillales*	*Listeriaceae*	*Brochothrix*	2,28778E-05	0	0,285049407
Bacteria	*Actinobacteria*	*Actinobacteria*	*Actinomycetales*	*Propionibacteriaceae*	*Brooklawnia*	0	2,82927E-06	0,082355525
Bacteria	*Proteobacteria*	*Alphaproteobacteria*	*Rhizobiales*	*Brucellaceae*	*Brucella*	7,51376E-06	0	0,000440292
Bacteria	*Firmicutes*	*Erysipelotrichia*	*Erysipelotrichales*	*Erysipelotrichaceae*	*Bulleidia*	4,98691E-05	0,001675337	0,027750993
Bacteria	*Firmicutes*	*Clostridia*	*Clostridiales*	*Ruminococcaceae*	*Butyricicoccus*	0,000155912	0,001132204	0,064077507
Bacteria	*Bacteroidetes*	*Bacteroidia*	*Bacteroidales*	*Porphyromonadaceae*	*Butyricimonas*	0,000016587	0,000193007	0,22251223
Bacteria	*Firmicutes*	*Clostridia*	*Clostridiales*	*Lachnospiraceae*	*Butyrivibrio*	0	0,000532976	0,000636299
Bacteria	*Proteobacteria*	*Epsilonproteobacteria*	*Campylobacterales*	*Campylobacteraceae*	*Campylobacter*	7,01854E-05	0,018825711	0,020524464
Bacteria	*Proteobacteria*	*Gammaproteobacteria*	*Candidatus Carsonella*	*Candidatus Carsonella*	*Candidatus Carsonella*	4,22904E-05	4,87934E-06	0,002807439
Bacteria	*Proteobacteria*	*Betaproteobacteria*	*Burkholderiales*	*Alcaligenaceae*	*Candidimonas*	6,36817E-06	0	0,285049407
Bacteria	*Bacteroidetes*	*Flavobacteriia*	*Flavobacteriales*	*Flavobacteriaceae*	*Capnocytophaga*	1,88001E-05	8,67288E-06	0,408368921
Bacteria	*Firmicutes*	*Bacilli*	*Lactobacillales*	*Carnobacteriaceae*	*Carnobacterium*	9,18491E-06	0	0,285049407
Bacteria	*Firmicutes*	*Bacilli*	*Bacillales*	*Planococcaceae*	*Caryophanon*	4,27148E-05	6,73019E-05	1
Bacteria	*Proteobacteria*	*Betaproteobacteria*	*Burkholderiales*	*Alcaligenaceae*	*Castellaniella*	2,78607E-05	0	0,285049407

Bacteria	*Firmicutes*	*Bacilli*	*Lactobacillales*	*Carnobacteriaceae*	*Catellicoccus*	3,40273E-07	5,99838E-07	1
Bacteria	*Firmicutes*	*Erysipelotrichia*	*Erysipelotrichales*	*Erysipelotrichaceae*	*Catenibacterium*	0,000140752	0,001354201	0,082579274
Bacteria	*Firmicutes*	*Clostridia*	*Clostridiales*	*Lachnospiraceae*	*Catonella*	1,60714E-05	9,54332E-06	1
Bacteria	*Actinobacteria*	*Actinobacteria*	*Actinomycetales*	*Cellulomonadaceae*	*Cellulomonas*	1,31429E-06	0	0,285049407
Bacteria	*Firmicutes*	*Clostridia*	*Clostridiales*	*Ruminococcaceae*	*Cellulosibacter*	0	1,09779E-05	0,006111784
Bacteria	*Firmicutes*	*Clostridia*	*Clostridiales*	*Lachnospiraceae*	*Cellulosilyticum*	0	2,02009E-05	0,037588881
Bacteria	*Proteobacteria*	*Gammaproteobacteria*	*Pseudomonadales*	*Pseudomonadaceae*	*Cellvibrio*	5,23914E-06	0	0,117601295
Bacteria	*Firmicutes*	*Bacilli*	*Bacillales*	*Bacillaceae 2*	*Cerasibacillus*	6,57144E-07	0	0,285049407
Bacteria	*Fusobacteria*	*Fusobacteriia*	*Fusobacteriales*	*Fusobacteriaceae*	*Cetobacterium*	1,36109E-06	5,09863E-06	1
Bacteria	*Bacteroidetes*	*Flavobacteriia*	*Flavobacteriales*	*Flavobacteriaceae*	*Chryseobacterium*	0,000128699	0,00014261	0,282058876
Bacteria	*Proteobacteria*	*Gammaproteobacteria*	*Enterobacteriales*	*Enterobacteriaceae*	*Citrobacter*	6,20085E-05	5,38548E-05	0,018422126
Bacteria	*Synergistetes*	*Synergistia*	*Synergistales*	*Synergistaceae*	*Cloacibacillus*	7,06474E-05	0,000244115	0,706491451
Bacteria	*Bacteroidetes*	*Flavobacteriia*	*Flavobacteriales*	*Flavobacteriaceae*	*Cloacibacterium*	0,000168514	0	0,005853245
Bacteria	*Firmicutes*	*Clostridia*	*Clostridiales*	*Ruminococcaceae*	*Clostridium III*	4,23919E-06	4,32807E-05	0,023856401
Bacteria	*Firmicutes*	*Clostridia*	*Clostridiales*	*Ruminococcaceae*	*Clostridium IV*	0,000278252	0,003695407	0,132463567
Bacteria	*Firmicutes*	*Clostridia*	*Clostridiales*	*Clostridiaceae 1*	*Clostridium sensu stricto*	0,004729025	0,004227616	0,562833754
Bacteria	*Firmicutes*	*Clostridia*	*Clostridiales*	*Peptostreptococcaceae*	*Clostridium XI*	0,003988915	0,001739411	0,020637558
Bacteria	*Fusobacteria*	*Fusobacteriia*	*Fusobacteriales*	*Fusobacteriaceae*	*Clostridium XIX*	5,28246E-06	0,00000271	0,695298567

Bacteria	*Firmicutes*	*Clostridia*	*Clostridiales*	*Lachnospiraceae*	*Clostridium XlVa*	0,001371008	0,00836941	0,105192505
Bacteria	*Firmicutes*	*Clostridia*	*Clostridiales*	*Lachnospiraceae*	*Clostridium XlVb*	0,000101329	0,000572136	0,201399822
Bacteria	*Firmicutes*	*Erysipelotrichia*	*Erysipelotrichales*	*Erysipelotrichaceae*	*Clostridium XVIII*	2,19658E-05	4,62949E-07	0,045714365
Bacteria	*Actinobacteria*	*Actinobacteria*	*Coriobacteriales*	*Coriobacteriaceae*	*Collinsella*	3,16375E-05	0	0,005853245
Bacteria	*Proteobacteria*	*Betaproteobacteria*	*Burkholderiales*	*Comamonadaceae*	*Comamonas*	0,001623778	7,65938E-05	0,014997039
Bacteria	*Proteobacteria*	*Betaproteobacteria*	*Neisseriales*	*Neisseriaceae*	*Conchiformibius*	2,12693E-05	0	0,047105791
Bacteria	*Firmicutes*	*Erysipelotrichia*	*Erysipelotrichales*	*Erysipelotrichaceae*	*Coprobacillus*	8,73837E-06	1,05067E-06	0,65591049
Bacteria	*Firmicutes*	*Clostridia*	*Clostridiales*	*Lachnospiraceae*	*Coprococcus*	0,000221351	0,00159338	0,007773957
Bacteria	*Verrucomicrobia*	*Opitutae*	*Puniceicoccales*	*Puniceicoccaceae*	*Coraliomargarita*	0	6,92428E-06	0,17090352
Bacteria	*Actinobacteria*	*Actinobacteria*	*Actinomycetales*	*Corynebacteriaceae*	*Corynebacterium*	0,001968928	0,003379973	0,297621468
Bacteria	*Bacteroidetes*	*Flavobacteriia*	*Flavobacteriales*	*Flavobacteriaceae*	*Cruoricaptor*	0	1,20268E-06	0,17090352
Bacteria	*Proteobacteria*	*Betaproteobacteria*	*Burkholderiales*	*Burkholderiaceae*	*Cupriavidus*	0	2,78137E-06	0,17090352
Bacteria	*Actinobacteria*	*Actinobacteria*	*Actinomycetales*	*Microbacteriaceae*	*Curtobacterium*	0	2,99919E-07	0,349574806
Bacteria	*Proteobacteria*	*Betaproteobacteria*	*Rhodocyclales*	*Rhodocyclaceae*	*Dechloromonas*	2,11959E-06	0	0,285049407
Bacteria	*Firmicutes*	*Clostridia*	*Clostridiales*	*Defluviitaleaceae*	*Defluviitalea*	3,06246E-06	7,10324E-06	0,270306711
Bacteria	*Deinococcus-Thermus*	*Deinococci*	*Deinococcales*	*Deinococcaceae*	*Deinococcus*	6,57144E-06	1,07971E-05	1
Bacteria	*Proteobacteria*	*Betaproteobacteria*	*Burkholderiales*	*Comamonadaceae*	*Delftia*	0,000143308	7,29849E-05	0,049140834
Bacteria	*Actinobacteria*	*Actinobacteria*	*Actinomycetales*	*Demequinaceae*	*Demequina*	0,000001791	0	0,285049407

Bacteria	*Actinobacteria*	*Actinobacteria*	*Actinomycetales*	*Dermacoccaceae*	*Dermacoccus*	3,03361E-05	0	0,047105791
Bacteria	*Actinobacteria*	*Actinobacteria*	*Actinomycetales*	*Dermatophilaceae*	*Dermatophilus*	0	0,000120615	0,082355525
Bacteria	*Firmicutes*	*Bacilli*	*Lactobacillales*	*Carnobacteriaceae*	*Desemzia*	0	9,25898E-07	0,349574806
Bacteria	*Proteobacteria*	*Deltaproteobacteria*	*Desulfobacterales*	*Desulfobulbaceae*	*Desulfobulbus*	0	2,09943E-06	0,349574806
Bacteria	*Proteobacteria*	*Deltaproteobacteria*	*Desulfovibrionales*	*Desulfovibrionaceae*	*Desulfovibrio*	7,28887E-05	0,003612145	0,048332655
Bacteria	*Proteobacteria*	*Alphaproteobacteria*	*Rhizobiales*	*Hyphomicrobiaceae*	*Devosia*	1,45887E-05	8,99757E-07	0,456570763
Bacteria	*Firmicutes*	*Negativicutes*	*Selenomonadales*	*Veillonellaceae*	*Dialister*	7,16704E-05	0,001664206	0,409395486
Bacteria	*Proteobacteria*	*Betaproteobacteria*	*Burkholderiales*	*Comamonadaceae*	*Diaphorobacter*	6,11262E-05	4,34649E-06	0,010177442
Bacteria	*Actinobacteria*	*Actinobacteria*	*Actinomycetales*	*Dietziaceae*	*Dietzia*	5,41814E-05	0	0,005853245
Bacteria	*Firmicutes*	*Bacilli*	*Lactobacillales*	*Carnobacteriaceae*	*Dolosigranulum*	0	2,99919E-07	0,349574806
Bacteria	*Proteobacteria*	*Alphaproteobacteria*	*Rhodospirillales*	*Rhodospirillaceae*	*Dongia*	2,46823E-06	0	0,117601295
Bacteria	*Firmicutes*	*Clostridia*	*Clostridiales*	*Lachnospiraceae*	*Dorea*	0,00018525	0,001203876	0,105192505
Bacteria	*Bacteroidetes*	*Bacteroidia*	*Bacteroidales*	*Porphyromonadaceae*	*Dysgonomonas*	6,20335E-05	0,000039816	0,414553486
Bacteria	*Bacteroidetes*	*Flavobacteriia*	*Flavobacteriales*	*Flavobacteriaceae*	*Elizabethkingia*	0	0,000452084	0,006111784
Bacteria	*Elusimicrobia*	*Elusimicrobia*	*Elusimicrobiales*	*Elusimicrobiaceae*	*Elusimicrobium*	2,43106E-05	0,000135628	0,050961937
Bacteria	*Bacteroidetes*	*Flavobacteriia*	*Flavobacteriales*	*Flavobacteriaceae*	*Empedobacter*	0,000120925	0,000103145	0,714393038
Bacteria	*Proteobacteria*	*Gammaproteobacteria*	*Pseudomonadales*	*Moraxellaceae*	*Enhydrobacter*	7,18973E-05	2,79956E-05	0,179300898
Bacteria	*Proteobacteria*	*Gammaproteobacteria*	*Enterobacteriales*	*Enterobacteriaceae*	*Enterobacter*	2,30968E-05	1,07794E-06	0,881930721

Bacteria	*Firmicutes*	*Bacilli*	*Lactobacillales*	*Enterococcaceae*	*Enterococcus*	0,000312725	0,000277936	1
Bacteria	*Actinobacteria*	*Actinobacteria*	*Coriobacteriales*	*Coriobacteriaceae*	*Enterorhabdus*	0,000005877	7,39733E-06	0,562548843
Bacteria	*Firmicutes*	*Erysipelotrichia*	*Erysipelotrichales*	*Erysipelotrichaceae*	*Erysipelothrix*	2,51857E-05	0,000185412	0,048332655
Bacteria	*Firmicutes*	*Erysipelotrichia*	*Erysipelotrichales*	*Erysipelotrichaceae*	*Erysipelotrichaceae_incertae_sedis*	0,000115114	0,002037731	0,049140834
Bacteria	*Proteobacteria*	*Gammaproteobacteria*	*Enterobacteriales*	*Enterobacteriaceae*	*Escherichia/Shigella*	0,000992541	0,011752799	0,417886964
Bacteria	*Firmicutes*	*Clostridia*	*Clostridiales*	*Ruminococcaceae*	*Ethanoligenens*	0	2,85123E-05	0,006111784
Bacteria	*Firmicutes*	*Clostridia*	*Clostridiales*	*Eubacteriaceae*	*Eubacterium*	0,000139422	0,00657523	0,027890529
Bacteria	*Firmicutes*	*Bacilli*	*Bacillales*	*Bacillales_Incertae Sedis XII*	*Exiguobacterium*	6,24715E-06	0	0,117601295
Bacteria	*Firmicutes*	*Bacilli*	*Lactobacillales*	*Aerococcaceae*	*Facklamia*	0,000315975	0,000367323	0,026505644
Bacteria	*Firmicutes*	*Clostridia*	*Clostridiales*	*Ruminococcaceae*	*Faecalibacterium*	0,000216487	0,014128783	0,010896354
Bacteria	*Firmicutes*	*Clostridia*	*Clostridiales*	*Ruminococcaceae*	*Fastidiosipila*	1,84454E-05	0,00011547	0,625585232
Bacteria	*Fibrobacteres*	*Fibrobacteria*	*Fibrobacterales*	*Fibrobacteraceae*	*Fibrobacter*	7,59818E-05	0,00109288	0,077099872
Bacteria	*Firmicutes*	*Clostridia*	*Clostridiales*	*Peptostreptococcaceae*	*Filifactor*	3,16835E-05	3,17815E-06	0,65591049
Bacteria	*Bacteroidetes*	*Sphingobacteriia*	*Sphingobacteriales*	*Chitinophagaceae*	*Filimonas*	2,88376E-05	1,04998E-05	0,113048724
Bacteria	*Firmicutes*	*Clostridia*	*Clostridiales*	*Clostridiales_Incertae Sedis XI*	*Finegoldia*	2,06313E-05	0,000010271	0,766432717
Bacteria	*Bacteroidetes*	*Sphingobacteriia*	*Sphingobacteriales*	*Chitinophagaceae*	*Flavihumibacter*	3,40273E-07	0	0,285049407
Bacteria	*Bacteroidetes*	*Sphingobacteriia*	*Sphingobacteriales*	*Chitinophagaceae*	*Flavitalea*	1,30143E-06	0	0,285049407
Bacteria	*Bacteroidetes*	*Flavobacteriia*	*Flavobacteriales*	*Flavobacteriaceae*	*Flavobacterium*	0,001038177	6,01473E-05	0,188593442

Bacteria	*Firmicutes*	*Clostridia*	*Clostridiales*	*Ruminococcaceae*	*Flavonifractor*	8,21808E-05	0,0017575 43	0,02063 7558
Bacteria	*Bacteroidetes*	*Flavobacteriia*	*Flavobacteriales*	*Cryomorphaceae*	*Fluviicola*	9,57215E-05	0	0,28504 9407
Bacteria	*Fusobacteria*	*Fusobacteriia*	*Fusobacteriales*	*Fusobacteriaceae*	*Fusobacterium*	0,000955587	0,0002899 78	0,06407 7507
Bacteria	*Firmicutes*	*Clostridia*	*Clostridiales*	*Clostridiales_Incertae Sedis XI*	*Gallicola*	1,37536E-05	8,11067E-05	0,80177 6827
Bacteria	*Bacteroidetes*	*Flavobacteriia*	*Flavobacteriales*	*Flavobacteriaceae*	*Gelidibacter*	0	1,4996E-06	0,34957 4806
Bacteria	*Firmicutes*	*Bacilli*	*Bacillales*	*Bacillales_Incertae Sedis XI*	*Gemella*	0,000272139	1,82939E-05	0,22251 223
Bacteria	*Planctomycetes*	*Planctomycetia*	*Planctomycetales*	*Planctomycetaceae*	*Gemmata*	1,29304E-05	0	0,28504 9407
Bacteria	*Gemmatimonadetes*	*Gemmatimonadetes*	*Gemmatimonadales*	*Gemmatimonadaceae*	*Gemmatimonas*	0,000000862	0	0,28504 9407
Bacteria	*Firmicutes*	*Clostridia*	*Clostridiales*	*Ruminococcaceae*	*Gemmiger*	0,00018848	0,0125043 24	0,01508 7256
Bacteria	*Actinobacteria*	*Actinobacteria*	*Actinomycetales*	*Bogoriellaceae*	*Georgenia*	0	2,91662E-07	0,34957 4806
Bacteria	*Bacteroidetes*	*Flavobacteriia*	*Flavobacteriales*	*Flavobacteriaceae*	*Gillisia*	0	4,73698E-07	0,34957 4806
Bacteria	*Firmicutes*	*Bacilli*	*Lactobacillales*	*Aerococcaceae*	*Globicatella*	0,00020017	0,0017711 78	0,29589 0126
Bacteria	*Actinobacteria*	*Actinobacteria*	*Actinomycetales*	*Glycomycetaceae*	*Glycomyces*	1,97143E-06	0	0,28504 9407
Bacteria	*Actinobacteria*	*Actinobacteria*	*Actinomycetales*	*Nocardiaceae*	*Gordonia*	3,14576E-05	0	0,11760 1295
Bacteria	*Actinobacteria*	*Actinobacteria*	*Coriobacteriales*	*Coriobacteriaceae*	*Gordonibacter*	0,000001724	3,66206E-06	0,37285 7936
Bacteria	*Acidobacteria*	*Acidobacteria_Gp16*	*Gp16*	*Gp16*	*Gp16*	8,54287E-06	0	0,28504 9407
Bacteria	*Acidobacteria*	*Acidobacteria_Gp6*	*Gp6*	*Gp6*	*Gp6*	3,44809E-06	0	0,28504 9407
Bacteria	*Acidobacteria*	*Acidobacteria_Gp7*	*Gp7*	*Gp7*	*Gp7*	3,18408E-06	0	0,28504 9407

Bacteria	*Cyanobacteria/Chloroplast*	*Cyanobacteria*	*Family I*	*Family I*	*GpI*	1,71022E-05	0	0,117601295
Bacteria	*Firmicutes*	*Bacilli*	*Lactobacillales*	*Carnobacteriaceae*	*Granulicatella*	7,53363E-05	2,95999E-05	0,338979844
Bacteria	*Actinobacteria*	*Actinobacteria*	*Actinomycetales*	*Propionibacteriaceae*	*Granulicoccus*	0	2,99919E-07	0,349574806
Bacteria	*Firmicutes*	*Clostridia*	*Clostridiales*	*Clostridiales_Incertae Sedis XII*	*Guggenheimella*	4,16191E-06	4,18101E-05	0,372857936
Bacteria	*Actinobacteria*	*Actinobacteria*	*Actinomycetales*	*Microbacteriaceae*	*Gulosibacter*	4,57712E-06	2,99919E-07	0,844911363
Bacteria	*Proteobacteria*	*Gammaproteobacteria*	*Pasteurellales*	*Pasteurellaceae*	*Haemophilus*	0,138797358	0,028801318	0,015087256
Bacteria	*Bacteroidetes*	*Bacteroidia*	*Bacteroidales*	*Prevotellaceae*	*Hallella*	2,08979E-05	0,002399751	0,027333767
Bacteria	*Actinobacteria*	*Actinobacteria*	*Actinomycetales*	*Ruaniaceae*	*Haloactinobacterium*	0	3,89895E-06	0,349574806
Bacteria	*Firmicutes*	*Bacilli*	*Bacillales*	*Bacillaceae 2*	*Halolactibacillus*	0	1,19968E-06	0,349574806
Bacteria	*Firmicutes*	*Clostridia*	*Clostridiales*	*Clostridiales_Incertae Sedis XI*	*Helcococcus*	6,64828E-05	0,001677115	0,55568979
Bacteria	*Proteobacteria*	*Epsilonproteobacteria*	*Campylobacterales*	*Helicobacteraceae*	*Helicobacter*	5,92942E-06	0,000135159	0,068743836
Bacteria	*Firmicutes*	*Clostridia*	*Clostridiales*	*Lachnospiraceae*	*Hespellia*	0	6,96344E-06	0,015891197
Bacteria	*Firmicutes*	*Erysipelotrichia*	*Erysipelotrichales*	*Erysipelotrichaceae*	*Holdemania*	8,33623E-05	0,003027302	0,027750993
Bacteria	*Firmicutes*	*Clostridia*	*Clostridiales*	*Lachnospiraceae*	*Howardella*	0	3,19799E-05	0,015891197
Bacteria	*Firmicutes*	*Clostridia*	*Clostridiales*	*Ruminococcaceae*	*Hydrogenoanaerobacterium*	0	3,66177E-05	0,002102622
Bacteria	*Proteobacteria*	*Betaproteobacteria*	*Burkholderiales*	*Comamonadaceae*	*Hydrogenophaga*	3,31524E-05	0	0,117601295
Bacteria	*Proteobacteria*	*Betaproteobacteria*	*Hydrogenophilales*	*Hydrogenophilaceae*	*Hydrogenophilus*	0,000000991	0	0,117601295
Bacteria	*Bacteroidetes*	*Sphingobacteriia*	*Sphingobacteriales*	*Chitinophagaceae*	*Hydrotalea*	6,80546E-07	0	0,285049407

Bacteria	*Proteobacteria*	*Alphaproteobacteria*	*Rhizobiales*	*Hyphomicrobiaceae*	*Hyphomicrobium*	6,03417E-06	1,02386E-05	0,214919521
Bacteria	*Proteobacteria*	*Gammaproteobacteria*	*Xanthomonadales*	*Xanthomonadaceae*	*Ignatzschineria*	1,37092E-05	0	0,117601295
Bacteria	*Actinobacteria*	*Actinobacteria*	*Acidimicrobiales*	*Acidimicrobiaceae*	*Ilumatobacter*	0,000001791	2,33816E-05	0,619656703
Bacteria	*Proteobacteria*	*Alphaproteobacteria*	*Rhodospirillales*	*Rhodospirillaceae*	*Insolitispirillum*	3,31343E-05	5,87721E-07	0,045714365
Bacteria	*Firmicutes*	*Bacilli*	*Lactobacillales*	*Carnobacteriaceae*	*Isobaculum*	0,000000995	2,81489E-06	0,234763663
Bacteria	*Actinobacteria*	*Actinobacteria*	*Actinomycetales*	*Intrasporangiaceae*	*Janibacter*	0,000007565	7,2242E-06	0,881930721
Bacteria	*Proteobacteria*	*Betaproteobacteria*	*Burkholderiales*	*Oxalobacteraceae*	*Janthinobacterium*	1,39524E-05	0	0,047105791
Bacteria	*Firmicutes*	*Bacilli*	*Bacillales*	*Staphylococcaceae*	*Jeotgalicoccus*	0,000100408	0,000196838	0,282058876
Bacteria	*Firmicutes*	*Clostridia*	*Clostridiales*	*Lachnospiraceae*	*Johnsonella*	0,000000199	0	0,285049407
Bacteria	*Actinobacteria*	*Actinobacteria*	*Actinomycetales*	*Jonesiaceae*	*Jonesia*	2,82613E-06	0	0,285049407
Bacteria	*Firmicutes*	*Erysipelotrichia*	*Erysipelotrichales*	*Erysipelotrichaceae*	*Kandleria*	0	5,88275E-06	0,17090352
Bacteria	*Proteobacteria*	*Betaproteobacteria*	*Burkholderiales*	*Alcaligenaceae*	*Kerstersia*	0,000000398	0	0,285049407
Bacteria	*Actinobacteria*	*Actinobacteria*	*Actinomycetales*	*Kineosporiaceae*	*Kineococcus*	0,000037929	0	0,285049407
Bacteria	*Proteobacteria*	*Betaproteobacteria*	*Neisseriales*	*Neisseriaceae*	*Kingella*	0,003130715	0,001977631	0,164914823
Bacteria	*Proteobacteria*	*Gammaproteobacteria*	*Enterobacteriales*	*Enterobacteriaceae*	*Klebsiella*	0,001106914	0,000103761	0,037073147
Bacteria	*Actinobacteria*	*Actinobacteria*	*Actinomycetales*	*Micrococcaceae*	*Kocuria*	0,000112007	2,63599E-05	0,010197877
Bacteria	*Firmicutes*	*Bacilli*	*Bacillales*	*Planococcaceae*	*Kurthia*	3,35537E-05	0,000230548	0,625585232
Bacteria	*Actinobacteria*	*Actinobacteria*	*Actinomycetales*	*Dermacoccaceae*	*Kytococcus*	2,18913E-05	0	0,017484674

Bacteria	*Proteobacteria*	*Alphaproteobacteria*	*Rhizobiales*	*Xanthobacteraceae*	*Labrys*	0	2,47309E-06	0,349574806
Bacteria	*Firmicutes*	*Clostridia*	*Clostridiales*	*Lachnospiraceae*	*Lachnoanaerobaculum*	1,22178E-05	1,91984E-05	0,285992181
Bacteria	*Firmicutes*	*Clostridia*	*Clostridiales*	*Lachnospiraceae*	*Lachnobacterium*	0	1,16675E-05	0,037588881
Bacteria	*Firmicutes*	*Clostridia*	*Clostridiales*	*Lachnospiraceae*	*Lachnospira*	0	6,03365E-05	0,015891197
Bacteria	*Firmicutes*	*Clostridia*	*Clostridiales*	*Lachnospiraceae*	*Lachnospiracea_incertae_sedis*	0,000693852	0,012367029	0,132463567
Bacteria	*Firmicutes*	*Bacilli*	*Lactobacillales*	*Carnobacteriaceae*	*Lacticigenium*	7,06532E-07	0	0,285049407
Bacteria	*Firmicutes*	*Bacilli*	*Lactobacillales*	*Lactobacillaceae*	*Lactobacillus*	0,003277203	0,034557655	0,003813532
Bacteria	*Firmicutes*	*Bacilli*	*Lactobacillales*	*Streptococcaceae*	*Lactococcus*	4,39816E-06	0,0108555	0,002183045
Bacteria	*Firmicutes*	*Clostridia*	*Clostridiales*	*Lachnospiraceae*	*Lactonifactor*	7,06532E-07	4,60501E-05	0,012056639
Bacteria	*Firmicutes*	*Bacilli*	*Lactobacillales*	*Streptococcaceae*	*Lactovum*	4,37984E-05	2,40007E-05	0,417470854
Bacteria	*Proteobacteria*	*Betaproteobacteria*	*Burkholderiales*	*Comamonadaceae*	*Lampropedia*	0	4,42176E-07	0,349574806
Bacteria	*Bacteroidetes*	*Cytophagia*	*Cytophagales*	*Cytophagaceae*	*Leadbetterella*	2,60697E-05	0	0,285049407
Bacteria	*Proteobacteria*	*Gammaproteobacteria*	*Enterobacteriales*	*Enterobacteriaceae*	*Leclercia*	4,38096E-07	4,42176E-07	1
Bacteria	*Proteobacteria*	*Gammaproteobacteria*	*Enterobacteriales*	*Enterobacteriaceae*	*Leminorella*	0,000000199	0	0,285049407
Bacteria	*Fusobacteria*	*Fusobacteriia*	*Fusobacteriales*	*Leptotrichiaceae*	*Leptotrichia*	0,000108726	3,42739E-05	0,393153438
Bacteria	*Actinobacteria*	*Actinobacteria*	*Actinomycetales*	*Microbacteriaceae*	*Leucobacter*	3,35391E-05	9,29749E-06	0,113048724
Bacteria	*Firmicutes*	*Bacilli*	*Lactobacillales*	*Leuconostocaceae*	*Leuconostoc*	3,65688E-05	0,001122494	0,048332655
Bacteria	*Proteobacteria*	*Betaproteobacteria*	*Burkholderiales*	*Comamonadaceae*	*Limnohabitans*	9,33337E-06	1,39701E-05	0,490840373

Bacteria	*Proteobacteria*	*Gammaproteobacteria*	*Pasteurellales*	*Pasteurellaceae*	*Lonepinella*	0	4,42176E-07	0,349574806
Bacteria	*Proteobacteria*	*Gammaproteobacteria*	*Xanthomonadales*	*Xanthomonadaceae*	*Luteimonas*	0	3,29911E-06	0,349574806
Bacteria	*Actinobacteria*	*Actinobacteria*	*Actinomycetales*	*Propionibacteriaceae*	*Luteococcus*	8,40996E-05	7,13403E-05	0,144376523
Bacteria	*Verrucomicrobia*	*Verrucomicrobiae*	*Verrucomicrobiales*	*Verrucomicrobiaceae*	*Luteolibacter*	3,88131E-06	5,87721E-07	0,362848315
Bacteria	*Firmicutes*	*Clostridia*	*Clostridiales*	*Gracilibacteraceae*	*Lutispora*	6,89619E-06	6,18123E-06	0,740726595
Bacteria	*Firmicutes*	*Bacilli*	*Bacillales*	*Planococcaceae*	*Lysinibacillus*	0,000000995	0,00000743	0,298500252
Bacteria	*Proteobacteria*	*Gammaproteobacteria*	*Xanthomonadales*	*Xanthomonadaceae*	*Lysobacter*	2,38191E-06	0	0,285049407
Bacteria	*Firmicutes*	*Bacilli*	*Bacillales*	*Staphylococcaceae*	*Macrococcus*	1,13045E-05	0	0,285049407
Bacteria	*Bacteroidetes*	*Bacteroidia*	*Bacteroidales*	*Marinilabiliaceae*	*Mangroviflexus*	1,13045E-05	1,32653E-06	0,844911363
Bacteria	*Proteobacteria*	*Gammaproteobacteria*	*Pasteurellales*	*Pasteurellaceae*	*Mannheimia*	0,006325729	0,001890478	0,489953608
Bacteria	*Proteobacteria*	*Gammaproteobacteria*	*Oceanospirillales*	*Oceanospirillaceae*	*Marinospirillum*	2,93846E-05	4,79871E-06	0,362848315
Bacteria	*Actinobacteria*	*Actinobacteria*	*Actinomycetales*	*Nocardioidaceae*	*Marmoricola*	0,000003743	0	0,285049407
Bacteria	*Proteobacteria*	*Alphaproteobacteria*	*Rhizobiales*	*Aurantimonadaceae*	*Martelella*	0,000000398	0	0,285049407
Bacteria	*Firmicutes*	*Clostridia*	*Clostridiales*	*Lachnospiraceae*	*Marvinbryantia*	1,88492E-05	3,84986E-06	0,233073458
Bacteria	*Proteobacteria*	*Betaproteobacteria*	*Burkholderiales*	*Oxalobacteraceae*	*Massilia*	1,82364E-05	0	0,047105791
Bacteria	*Firmicutes*	*Negativicutes*	*Selenomonadales*	*Veillonellaceae*	*Megasphaera*	0,000107022	0,021632281	0,007773957
Bacteria	*Firmicutes*	*Bacilli*	*Lactobacillales*	*Enterococcaceae*	*Melissococcus*	0	2,91662E-07	0,349574806
Bacteria	*Bacteroidetes*	*Cytophagia*	*Cytophagales*	*Cytophagaceae*	*Meniscus*	0	4,73698E-07	0,349574806

Bacteria	*Proteobacteria*	*Alphaproteobacteria*	*Rhizobiales*	*Phyllobacteriaceae*	*Mesorhizobium*	1,15005E-05	3,25504E-05	0,949955982
Bacteria	*Actinobacteria*	*Actinobacteria*	*Bifidobacteriales*	*Bifidobacteriaceae*	*Metascardovia*	0	4,8573E-06	0,082355525
Archaea	*Euryarchaeota*	*Methanobacteria*	*Methanobacteriales*	*Methanobacteriaceae*	*Methanobrevibacter*	0,000174022	0,001034084	0,417886964
Archaea	*Euryarchaeota*	*Methanobacteria*	*Methanobacteriales*	*Methanobacteriaceae*	*Methanosphaera*	2,60286E-06	4,94619E-07	0,844911363
Bacteria	*Proteobacteria*	*Betaproteobacteria*	*Methylophilales*	*Methylophilaceae*	*Methylobacillus*	1,87551E-05	0	0,117601295
Bacteria	*Proteobacteria*	*Alphaproteobacteria*	*Rhizobiales*	*Methylobacteriaceae*	*Methylobacterium*	2,35034E-05	4,42176E-07	0,013115904
Bacteria	*Proteobacteria*	*Betaproteobacteria*	*Methylophilales*	*Methylophilaceae*	*Methylophilus*	1,27885E-05	0	0,117601295
Bacteria	*Actinobacteria*	*Actinobacteria*	*Actinomycetales*	*Microbacteriaceae*	*Microbacterium*	0,000880889	8,23771E-06	0,000817973
Bacteria	*Actinobacteria*	*Actinobacteria*	*Actinomycetales*	*Micrococcaceae*	*Micrococcus*	0,000180634	2,23779E-05	0,010197877
Bacteria	*Actinobacteria*	*Actinobacteria*	*Actinomycetales*	*Propionibacteriaceae*	*Microlunatus*	1,95214E-06	0	0,285049407
Bacteria	*Actinobacteria*	*Actinobacteria*	*Actinomycetales*	*Nocardiaceae*	*Millisia*	0,000000199	3,29911E-06	1
Bacteria	*Firmicutes*	*Negativicutes*	*Selenomonadales*	*Veillonellaceae*	*Mitsuokella*	4,15509E-05	0,00197205	0,007773957
Bacteria	*Firmicutes*	*Clostridia*	*Clostridiales*	*Clostridiales_Incertae Sedis XIII*	*Mogibacterium*	2,49425E-05	2,14525E-05	0,203017106
Bacteria	*Bacteroidetes*	*Flavobacteriia*	*Flavobacteriales*	*Flavobacteriaceae*	*Moheibacter*	2,77578E-05	2,98345E-06	0,270306711
Bacteria	*Proteobacteria*	*Gammaproteobacteria*	*Pseudomonadales*	*Moraxellaceae*	*Moraxella*	0,342219279	0,091731595	0,015087256
Bacteria	*Proteobacteria*	*Gammaproteobacteria*	*Enterobacteriales*	*Enterobacteriaceae*	*Morganella*	8,13932E-05	0	0,285049407
Bacteria	*Firmicutes*	*Clostridia*	*Clostridiales*	*Lachnospiraceae*	*Moryella*	1,67557E-06	0	0,117601295
Bacteria	*Actinobacteria*	*Actinobacteria*	*Actinomycetales*	*Motilibacter*	*Motilibacter*	0,000004555	0	0,285049407

Bacteria	*Deferribacteres*	*Deferribacteres*	*Deferribacterales*	*Deferribacteraceae*	*Mucispirillum*	2,28417E-05	0,001106287	0,209482376
Bacteria	*Firmicutes*	*Clostridia*	*Clostridiales*	*Incertae Sedis XI*	*Murdochiella*	1,64151E-05	8,76506E-05	0,850652329
Bacteria	*Actinobacteria*	*Actinobacteria*	*Actinomycetales*	*Mycobacteriaceae*	*Mycobacterium*	1,89645E-05	0	0,285049407
Bacteria	*Tenericutes*	*Mollicutes*	*Mycoplasmatales*	*Mycoplasmataceae*	*Mycoplasma*	0,030510822	0,005765404	0,010896354
Bacteria	*Bacteroidetes*	*Flavobacteriia*	*Flavobacteriales*	*Flavobacteriaceae*	*Myroides*	0,003350288	2,56877E-05	0,006716646
Bacteria	*Firmicutes*	*Clostridia*	*Clostridiales*	*Natranaerovirga*	*Natranaerovirga*	0	8,84352E-07	0,349574806
Bacteria	*Proteobacteria*	*Betaproteobacteria*	*Burkholderiales*	*Oxalobacteraceae*	*Naxibacter*	4,14041E-06	2,33937E-05	0,562548843
Bacteria	*Firmicutes*	*Negativicutes*	*Selenomonadales*	*Veillonellaceae*	*Negativicoccus*	0	4,94619E-07	0,349574806
Bacteria	*Proteobacteria*	*Betaproteobacteria*	*Neisseriales*	*Neisseriaceae*	*Neisseria*	0,049348792	0,04257156	0,35453948
Bacteria	*Actinobacteria*	*Actinobacteria*	*Actinomycetales*	*Micrococcaceae*	*Nesterenkonia*	6,53571E-05	2,99919E-07	0,000988911
Bacteria	*Proteobacteria*	*Gammaproteobacteria*	*Pasteurellales*	*Pasteurellaceae*	*Nicoletella*	2,58607E-06	0	0,285049407
Bacteria	*Proteobacteria*	*Gammaproteobacteria*	*Oceanospirillales*	*Oceanospirillaceae*	*Nitrincola*	0,000001194	0	0,285049407
Bacteria	*Proteobacteria*	*Alphaproteobacteria*	*Rhizobiales*	*Bradyrhizobiaceae*	*Nitrobacter*	1,05433E-05	0	0,117601295
Bacteria	*Actinobacteria*	*Actinobacteria*	*Actinomycetales*	*Nocardioidaceae*	*Nocardioides*	1,65612E-05	1,52574E-06	0,65591049
Bacteria	*Firmicutes*	*Bacilli*	*Bacillales*	*Staphylococcaceae*	*Nosocomiicoccus*	4,34529E-06	6,04831E-05	0,272253581
Bacteria	*Proteobacteria*	*Alphaproteobacteria*	*Sphingomonadales*	*Sphingomonadaceae*	*Novosphingobium*	5,54849E-05	0	0,017484674
Bacteria	*Proteobacteria*	*Gammaproteobacteria*	*Aeromonadales*	*Aeromonadaceae*	*Oceanimonas*	1,25373E-05	1,4996E-06	0,844911363
Bacteria	*Proteobacteria*	*Alphaproteobacteria*	*Rhizobiales*	*Brucellaceae*	*Ochrobactrum*	0,000889613	0,000028509	0,001076633

Bacteria	*Bacteroidetes*	*Bacteroidia*	*Bacteroidales*	*Porphyromonadaceae*	*Odoribacter*	6,56252E-06	7,25376E-05	0,490840373
Bacteria	*Bacteroidetes*	*Bacteroidetes"_incertae_sedis*	*Ohtaekwangia*	*Ohtaekwangia*	*Ohtaekwangia*	0	2,31475E-06	0,349574806
Bacteria	*Actinobacteria*	*Actinobacteria*	*Actinomycetales*	*Microbacteriaceae*	*Okibacterium*	8,79551E-07	0	0,117601295
Bacteria	*Proteobacteria*	*Betaproteobacteria*	*Burkholderiales*	*Alcaligenaceae*	*Oligella*	0,000000796	0	0,285049407
Bacteria	*Proteobacteria*	*Alphaproteobacteria*	*Rhizobiales*	*Bradyrhizobiaceae*	*Oligotropha*	0	8,915E-07	0,17090352
Bacteria	*Actinobacteria*	*Actinobacteria*	*Coriobacteriales*	*Coriobacteriaceae*	*Olsenella*	1,01113E-05	0,00010059	0,169282508
Bacteria	*Firmicutes*	*Clostridia*	*Clostridiales*	*Lachnospiraceae*	*Oribacterium*	0,000139212	0,000953139	0,04893855
Bacteria	*Actinobacteria*	*Actinobacteria*	*Actinomycetales*	*Intrasporangiaceae*	*Ornithinimicrobium*	0	2,98345E-06	0,17090352
Bacteria	*Bacteroidetes*	*Flavobacteriia*	*Flavobacteriales*	*Flavobacteriaceae*	*Ornithobacterium*	2,14444E-05	0,000055934	0,063939089
Bacteria	*Firmicutes*	*Clostridia*	*Clostridiales*	*Ruminococcaceae*	*Oscillibacter*	0,001180058	0,01423382	0,164914823
Bacteria	*Proteobacteria*	*Gammaproteobacteria*	*Pasteurellales*	*Pasteurellaceae*	*Otariodibacter*	6,48808E-05	4,67181E-05	0,643428844
Bacteria	*Bacteroidetes*	*Sphingobacteriia*	*Sphingobacteriales*	*Chitinophagaceae*	*Other*	0,026914734	0,008368843	0,048332655
Bacteria	*Other*	*Other*	*Other*	*Other*	*Other*	0,010044255	0,003342864	0,132463567
Bacteria	*Firmicutes*	*Clostridia*	*Clostridiales*	*Ruminococcaceae*	*Other*	0,002837881	0,024979279	0,082579274
Bacteria	*Firmicutes*	*Clostridia*	*Clostridiales*	*Other*	*Other*	0,001954701	0,018041689	0,132463567
Bacteria	*Firmicutes*	*Clostridia*	*Clostridiales*	*Lachnospiraceae*	*Other*	0,001358907	0,009779488	0,105192505
Bacteria	*Firmicutes*	*Negativicutes*	*Selenomonadales*	*Veillonellaceae*	*Other*	0,000138323	0,008259076	0,010825507
Bacteria	*Bacteroidetes*	*Bacteroidia*	*Bacteroidales*	*Porphyromonadaceae*	*Other*	0,000176862	0,006048777	0,064077507

Bacteria	*Bacteroidetes*	*Other*	*Other*	*Other*	*Other*	0,000426828	0,005515593	0,027890529
Bacteria	*Bacteroidetes*	*Flavobacteriia*	*Flavobacteriales*	*Flavobacteriaceae*	*Other*	0,000443254	0,005107163	0,105192505
Bacteria	*Bacteroidetes*	*Bacteroidia*	*Bacteroidales*	*Other*	*Other*	0,000445299	0,005062005	0,027890529
Bacteria	*Bacteroidetes*	*Bacteroidia*	*Bacteroidales*	*Prevotellaceae*	*Other*	3,52357E-05	0,00407461	0,042658104
Bacteria	*Proteobacteria*	*Gammaproteobacteria*	*Pasteurellales*	*Pasteurellaceae*	*Other*	0,004814278	0,003956373	0,203017106
Bacteria	*Firmicutes*	*Other*	*Other*	*Other*	*Other*	0,000230167	0,00344632	0,203017106
Bacteria	*Firmicutes*	*Bacilli*	*Lactobacillales*	*Enterococcaceae*	*Other*	0,003645571	0,00254444	0,561432374
Bacteria	*Firmicutes*	*Bacilli*	*Lactobacillales*	*Aerococcaceae*	*Other*	0,000220519	0,002536873	1
Bacteria	*Proteobacteria*	*Deltaproteobacteria*	*Other*	*Other*	*Other*	3,56846E-05	0,001213876	0,014195744
Bacteria	*Planctomycetes*	*Planctomycetia*	*Planctomycetales*	*Planctomycetaceae*	*Other*	7,97945E-05	0,001158077	0,063128008
Bacteria	*Firmicutes*	*Erysipelotrichia*	*Erysipelotrichales*	*Erysipelotrichaceae*	*Other*	6,22529E-05	0,000810452	0,020637558
Bacteria	*Bacteroidetes*	*Sphingobacteriia*	*Sphingobacteriales*	*Sphingobacteriaceae*	*Other*	0,000118235	0,000549154	0,642237837
Bacteria	*Firmicutes*	*Bacilli*	*Lactobacillales*	*Other*	*Other*	3,30442E-05	0,000508194	0,002621822
Bacteria	*Proteobacteria*	*Betaproteobacteria*	*Neisseriales*	*Neisseriaceae*	*Other*	0,001085817	0,000458841	0,105192505
Bacteria	*Firmicutes*	*Clostridia*	*Clostridiales*	*Clostridiales_Incertae Sedis XIII*	*Other*	1,52786E-05	0,000412218	0,020186618
Bacteria	*Proteobacteria*	*Deltaproteobacteria*	*Desulfovibrionales*	*Desulfovibrionaceae*	*Other*	5,87389E-05	0,000293713	0,090410706
Bacteria	*Firmicutes*	*Bacilli*	*Lactobacillales*	*Carnobacteriaceae*	*Other*	3,65281E-05	0,000290665	0,036566184
Bacteria	*Proteobacteria*	*Gammaproteobacteria*	*Enterobacteriales*	*Enterobacteriaceae*	*Other*	0,000034318	0,000288175	0,202613531

Unclassified	*Other*	*Other*	*Other*	*Other*	*Other*	0,0055402	0,000285374	0,001780211
Bacteria	*Actinobacteria*	*Actinobacteria*	*Coriobacteriales*	*Coriobacteriaceae*	*Other*	6,49454E-05	0,000240768	0,072593149
Bacteria	*Firmicutes*	*Clostridia*	*Clostridiales*	*Clostridiales_Incertae Sedis XI*	*Other*	6,58162E-05	0,000235456	0,346484118
Bacteria	*Proteobacteria*	*Alphaproteobacteria*	*Other*	*Other*	*Other*	6,40578E-06	0,000207409	0,001886168
Bacteria	*Firmicutes*	*Clostridia*	*Clostridiales*	*Clostridiaceae 1*	*Other*	6,66141E-05	0,000194602	0,064077507
Bacteria	*Proteobacteria*	*Betaproteobacteria*	*Burkholderiales*	*Other*	*Other*	0,000034557	0,000178001	0,201399822
Bacteria	*Firmicutes*	*Negativicutes*	*Selenomonadales*	*Acidaminococcaceae*	*Other*	0	0,000168738	0,006111784
Bacteria	*Actinobacteria*	*Actinobacteria*	*Actinomycetales*	*Other*	*Other*	0,000355748	0,000157894	0,082305182
Bacteria	*Proteobacteria*	*Gammaproteobacteria*	*Pseudomonadales*	*Moraxellaceae*	*Other*	0,000332247	0,000138773	0,104881801
Bacteria	*Proteobacteria*	*Betaproteobacteria*	*Other*	*Other*	*Other*	0,000102763	0,000130469	0,35453948
Bacteria	*Firmicutes*	*Negativicutes*	*Selenomonadales*	*Other*	*Other*	0,000001724	0,000121686	0,036713856
Bacteria	*Spirochaetes*	*Spirochaetia*	*Spirochaetales*	*Spirochaetaceae*	*Other*	0	0,000119004	0,015891197
Bacteria	*Proteobacteria*	*Gammaproteobacteria*	*Other*	*Other*	*Other*	0,000359929	0,000106706	0,005478553
Bacteria	*Firmicutes*	*Clostridia*	*Clostridiales*	*Incertae Sedis XI*	*Other*	0	9,27013E-05	0,082355525
Bacteria	*Proteobacteria*	*Other*	*Other*	*Other*	*Other*	0,000400693	8,42972E-05	0,015087256
Bacteria	*Firmicutes*	*Bacilli*	*Other*	*Other*	*Other*	1,31488E-05	7,28777E-05	0,131079232
Bacteria	*Firmicutes*	*Bacilli*	*Lactobacillales*	*Lactobacillaceae*	*Other*	2,38119E-06	7,07067E-05	0,000967429
Bacteria	*Proteobacteria*	*Epsilonproteobacteria*	*Campylobacterales*	*Other*	*Other*	0	5,18825E-05	0,006111784

Bacteria	*Firmicutes*	*Clostridia*	*Other*	*Other*	*Other*	7,78551E-06	5,13085E-05	0,019769406
Bacteria	*Proteobacteria*	*Deltaproteobacteria*	*Desulfovibrionales*	*Other*	*Other*	0	0,00002425	0,006111784
Bacteria	*Bacteroidetes*	*Bacteroidia*	*Bacteroidales*	*Marinilabiaceae*	*Other*	0	0,000023066	0,002102622
Bacteria	*Synergistetes*	*Synergistia*	*Synergistales*	*Synergistaceae*	*Other*	3,04167E-05	2,27804E-05	0,625585232
Bacteria	*Firmicutes*	*Bacilli*	*Lactobacillales*	*Streptococcaceae*	*Other*	3,9297E-06	2,04634E-05	0,625585232
Bacteria	*Fusobacteria*	*Fusobacteriia*	*Fusobacteriales*	*Leptotrichiaceae*	*Other*	4,08328E-06	2,01207E-05	0,619656703
Bacteria	*Proteobacteria*	*Alphaproteobacteria*	*Rhodospirillales*	*Rhodospirillaceae*	*Other*	0,000005749	0,000019618	0,148871993
Bacteria	*Proteobacteria*	*Betaproteobacteria*	*Burkholderiales*	*Sutterellaceae*	*Other*	0	1,63834E-05	0,037588881
Bacteria	*Spirochaetes*	*Spirochaetia*	*Spirochaetales*	*Other*	*Other*	0	1,16735E-05	0,037588881
Bacteria	*Actinobacteria*	*Actinobacteria*	*Actinomycetales*	*Micrococcaceae*	*Other*	1,82226E-05	1,08583E-05	1
Bacteria	*Proteobacteria*	*Betaproteobacteria*	*Burkholderiales*	*Alcaligenaceae*	*Other*	9,35325E-06	9,65522E-06	0,372857936
Bacteria	*Proteobacteria*	*Alphaproteobacteria*	*Sphingomonadales*	*Other*	*Other*	0,000000199	9,29749E-06	1
Bacteria	*Actinobacteria*	*Actinobacteria*	*Actinomycetales*	*Corynebacteriaceae*	*Other*	4,38096E-07	9,06842E-06	0,050186187
Bacteria	*Proteobacteria*	*Gammaproteobacteria*	*Pseudomonadales*	*Other*	*Other*	2,99795E-05	9,00509E-06	0,048595087
Bacteria	*Proteobacteria*	*Epsilonproteobacteria*	*Other*	*Other*	*Other*	0	8,17793E-06	0,037588881
Bacteria	*Verrucomicrobia*	*Opitutae*	*Puniceicoccales*	*Puniceicoccaceae*	*Other*	0	6,58282E-06	0,17090352
Bacteria	*Actinobacteria*	*Actinobacteria*	*Other*	*Other*	*Other*	0	6,2983E-06	0,349574806
Bacteria	*Fusobacteria*	*Fusobacteriia*	*Fusobacteriales*	*Fusobacteriaceae*	*Other*	2,95552E-06	5,68375E-06	0,896124778

Bacteria	*Proteobacteria*	*Betaproteobacteria*	*Burkholderiales*	*Comamonadaceae*	*Other*	4,68385E-05	5,54533E-06	0,238592829
Bacteria	*Actinobacteria*	*Actinobacteria*	*Actinomycetales*	*Propionibacteriaceae*	*Other*	0,000010175	5,39854E-06	0,456570763
Bacteria	*Firmicutes*	*Bacilli*	*Bacillales*	*Other*	*Other*	5,67973E-05	5,16472E-06	0,07886029
Bacteria	*Bacteroidetes*	*Sphingobacteriia*	*Sphingobacteriales*	*Other*	*Other*	6,80546E-07	0,000004425	0,372857936
Bacteria	*Proteobacteria*	*Alphaproteobacteria*	*Rhizobiales*	*Bradyrhizobiaceae*	*Other*	0,000000219	3,46156E-06	0,234763663
Bacteria	*Proteobacteria*	*Gammaproteobacteria*	*Aeromonadales*	*Succinivibrionaceae*	*Other*	0	3,18907E-06	0,082355525
Bacteria	*Lentisphaerae*	*Other*	*Other*	*Other*	*Other*	0	3,09523E-06	0,349574806
Bacteria	*Cyanobacteria/Chloroplast*	*Chloroplast*	*Chloroplast*	*Chloroplast*	*Other*	0,000000219	2,95893E-06	1
Bacteria	*Tenericutes*	*Mollicutes*	*Other*	*Other*	*Other*	0	2,9386E-06	0,349574806
Bacteria	*Firmicutes*	*Clostridia*	*Clostridiales*	*Peptostreptococcaceae*	*Other*	5,40363E-06	2,67175E-06	0,329113986
Bacteria	*Proteobacteria*	*Betaproteobacteria*	*Burkholderiales*	*Burkholderiales_incertae_sedis*	*Other*	6,56561E-06	2,65305E-06	0,456570763
Bacteria	*Bacteroidetes*	*Cytophagia*	*Cytophagales*	*Other*	*Other*	0	2,64751E-06	0,17090352
Bacteria	*Proteobacteria*	*Alphaproteobacteria*	*Sphingomonadales*	*Sphingomonadaceae*	*Other*	0	0,000002625	0,349574806
Bacteria	*Actinobacteria*	*Actinobacteria*	*Actinomycetales*	*Dermacoccaceae*	*Other*	0,000001724	2,28861E-06	0,740726595
Bacteria	*Actinobacteria*	*Actinobacteria*	*Actinomycetales*	*Microbacteriaceae*	*Other*	1,43262E-05	2,0752E-06	0,020462223
Bacteria	*Firmicutes*	*Clostridia*	*Clostridiales*	*Eubacteriaceae*	*Other*	0	1,94714E-06	0,082355525
Bacteria	*Actinobacteria*	*Actinobacteria*	*Actinomycetales*	*Intrasporangiaceae*	*Other*	0	1,79951E-06	0,349574806
Bacteria	*Verrucomicrobia*	*Verrucomicrobiae*	*Verrucomicrobiales*	*Verrucomicrobiaceae*	*Other*	1,41306E-06	1,76963E-06	0,457717434

Bacteria	*Proteobacteria*	*Deltaproteobacteria*	*Desulfovibrionales*	*Desulfohalobiaceae*	*Other*	0	1,7687E-06	0,349574806
Bacteria	*Actinobacteria*	*Actinobacteria*	*Actinomycetales*	*Dermabacteraceae*	*Other*	0,00000126	1,66263E-06	1
Bacteria	*Actinobacteria*	*Actinobacteria*	*Actinomycetales*	*Dermatophilaceae*	*Other*	1,13045E-05	1,4996E-06	0,844911363
Bacteria	*Proteobacteria*	*Betaproteobacteria*	*Rhodocyclales*	*Rhodocyclaceae*	*Other*	0	1,4996E-06	0,349574806
Archaea	*Other*	*Other*	*Other*	*Other*	*Other*	2,87398E-05	1,48748E-06	0,005020543
Bacteria	*Proteobacteria*	*Alphaproteobacteria*	*Rhizobiales*	*Hyphomicrobiaceae*	*Other*	0,000050848	1,47947E-06	0,004738512
Bacteria	*Proteobacteria*	*Alphaproteobacteria*	*Rhodobacterales*	*Rhodobacteraceae*	*Other*	0,000026016	1,39438E-06	0,270306711
Bacteria	*Proteobacteria*	*Gammaproteobacteria*	*Aeromonadales*	*Aeromonadaceae*	*Other*	5,57215E-06	1,36807E-06	0,740726595
Bacteria	*Firmicutes*	*Bacilli*	*Bacillales*	*Staphylococcaceae*	*Other*	7,19091E-05	1,35059E-06	0,187502587
Bacteria	*Actinobacteria*	*Actinobacteria*	*Acidimicrobiales*	*Other*	*Other*	0	1,19968E-06	0,349574806
Bacteria	*Bacteroidetes*	*Flavobacteriia*	*Flavobacteriales*	*Other*	*Other*	0,000000597	1,17544E-06	1
Archaea	*Euryarchaeota*	*Other*	*Other*	*Other*	*Other*	0	1,17544E-06	0,349574806
Bacteria	*Firmicutes*	*Clostridia*	*Clostridiales*	*Gracilibacteraceae*	*Other*	0	1,17544E-06	0,349574806
Archaea	*Crenarchaeota*	*Thermoprotei*	*Other*	*Other*	*Other*	7,7309E-06	1,06528E-06	0,148871993
Bacteria	*Verrucomicrobia*	*Other*	*Other*	*Other*	*Other*	0	1,06142E-06	0,17090352
Bacteria	*Firmicutes*	*Bacilli*	*Bacillales*	*Planococcaceae*	*Other*	3,5821E-06	0,000001042	0,740726595
Bacteria	*Proteobacteria*	*Alphaproteobacteria*	*Rhizobiales*	*Other*	*Other*	1,59298E-05	8,915E-07	0,023856401
Bacteria	*Proteobacteria*	*Betaproteobacteria*	*Burkholderiales*	*Oxalobacteraceae*	*Other*	7,45463E-06	8,84352E-07	0,137485208

Bacteria	*Proteobacteria*	*Gammaproteobacteria*	*Xanthomonadales*	*Xanthomonadaceae*	*Other*	6,64222E-06	5,99838E-07	0,010075007
Bacteria	*Proteobacteria*	*Alphaproteobacteria*	*Rhizobiales*	*Phyllobacteriaceae*	*Other*	1,41306E-06	5,99838E-07	0,844911363
Bacteria	*Bacteroidetes*	*Cytophagia*	*Cytophagales*	*Cytophagaceae*	*Other*	0	5,87721E-07	0,349574806
Bacteria	*Firmicutes*	*Clostridia*	*Clostridiales*	*Peptococcaceae 1*	*Other*	0	4,62949E-07	0,349574806
Bacteria	*Proteobacteria*	*Epsilonproteobacteria*	*Campylobacterales*	*Helicobacteraceae*	*Other*	0	4,42176E-07	0,349574806
Bacteria	*Proteobacteria*	*Gammaproteobacteria*	*Aeromonadales*	*Other*	*Other*	0	4,42176E-07	0,349574806
Bacteria	*Proteobacteria*	*Alphaproteobacteria*	*Rhodospirillales*	*Other*	*Other*	7,3632E-06	2,99919E-07	0,844911363
Bacteria	*Proteobacteria*	*Alphaproteobacteria*	*Rhodospirillales*	*Acetobacteraceae*	*Other*	0	2,99919E-07	0,349574806
Bacteria	*Firmicutes*	*Bacilli*	*Lactobacillales*	*Leuconostocaceae*	*Other*	0	2,91662E-07	0,349574806
Bacteria	*Proteobacteria*	*Gammaproteobacteria*	*Pseudomonadales*	*Pseudomonadaceae*	*Other*	9,07211E-05	0	0,005853245
Bacteria	*Firmicutes*	*Bacilli*	*Bacillales*	*Bacillaceae 1*	*Other*	4,20572E-05	0	0,285049407
Bacteria	*Proteobacteria*	*Alphaproteobacteria*	*Sphingomonadales*	*Erythrobacteraceae*	*Other*	2,25969E-05	0	0,117601295
Bacteria	*Actinobacteria*	*Actinobacteria*	*Actinomycetales*	*Kineosporiaceae*	*Other*	1,75127E-05	0	0,117601295
Bacteria	*Actinobacteria*	*Actinobacteria*	*Actinomycetales*	*Geodermatophilaceae*	*Other*	1,07278E-05	0	0,117601295
Bacteria	*Firmicutes*	*Bacilli*	*Bacillales*	*Bacillaceae 2*	*Other*	7,28833E-06	0	0,047105791
Bacteria	*Proteobacteria*	*Gammaproteobacteria*	*Oceanospirillales*	*Other*	*Other*	7,16419E-06	0	0,285049407
Bacteria	*Proteobacteria*	*Deltaproteobacteria*	*Myxococcales*	*Polyangiaceae*	*Other*	3,06246E-06	0	0,285049407
Bacteria	*Proteobacteria*	*Gammaproteobacteria*	*Vibrionales*	*Vibrionaceae*	*Other*	2,0101E-06	0	0,117601295

Bacteria	*Proteobacteria*	*Alphaproteobacteria*	*Caulobacterales*	*Caulobacteraceae*	*Other*	1,9431E-06	0	0,117601295
Bacteria	*Bacteroidetes*	*Flavobacteriia*	*Flavobacteriales*	*Cryomorphaceae*	*Other*	0,000000995	0	0,285049407
Bacteria	*Chloroflexi*	*Other*	*Other*	*Other*	*Other*	0,000000862	0	0,285049407
Bacteria	*Proteobacteria*	*Gammaproteobacteria*	*Oceanospirillales*	*Oceanospirillaceae*	*Other*	0,000000796	0	0,285049407
Bacteria	*Proteobacteria*	*Alphaproteobacteria*	*Rhizobiales*	*Brucellaceae*	*Other*	6,37101E-07	0	0,117601295
Bacteria	*Proteobacteria*	*Epsilonproteobacteria*	*Campylobacterales*	*Campylobacteraceae*	*Other*	0,000000398	0	0,285049407
Bacteria	*Proteobacteria*	*Betaproteobacteria*	*Burkholderiales*	*Oxalobacteraceae*	*Oxalobacter*	0	0,000116042	0,002102622
Bacteria	*Proteobacteria*	*Betaproteobacteria*	*Burkholderiales*	*Alcaligenaceae*	*Paenalcaligenes*	0,000242556	9,89237E-07	0,362848315
Bacteria	*Firmicutes*	*Bacilli*	*Bacillales*	*Paenibacillaceae 1*	*Paenibacillus*	1,99005E-06	0	0,285049407
Bacteria	*Proteobacteria*	*Alphaproteobacteria*	*Rhizobiales*	*Brucellaceae*	*Paenochrobactrum*	2,84578E-05	1,97847E-06	0,844911363
Bacteria	*Bacteroidetes*	*Bacteroidia*	*Bacteroidales*	*Porphyromonadaceae*	*Paludibacter*	0,00022408	0,000951321	0,245466862
Bacteria	*Proteobacteria*	*Gammaproteobacteria*	*Enterobacteriales*	*Enterobacteriaceae*	*Pantoea*	5,70972E-06	9,25898E-07	0,456570763
Bacteria	*Firmicutes*	*Clostridia*	*Clostridiales*	*Ruminococcaceae*	*Papillibacter*	2,84254E-05	0,00033782	0,151493992
Bacteria	*Bacteroidetes*	*Bacteroidia*	*Bacteroidales*	*Porphyromonadaceae*	*Parabacteroides*	0,000228437	0,000707313	0,48745329
Bacteria	*Proteobacteria*	*Alphaproteobacteria*	*Rhodobacterales*	*Rhodobacteraceae*	*Paracoccus*	0,000133918	3,83897E-05	0,010414995
Bacteria	*Actinobacteria*	*Actinobacteria*	*Coriobacteriales*	*Coriobacteriaceae*	*Paraeggerthella*	0,000010592	7,09705E-06	0,765087193
Bacteria	*Firmicutes*	*Bacilli*	*Lactobacillales*	*Lactobacillaceae*	*Paralactobacillus*	2,11959E-06	3,87194E-05	0,002807439
Bacteria	*Proteobacteria*	*Gammaproteobacteria*	*Pseudomonadales*	*Moraxellaceae*	*Paraperlucidibaca*	0	2,99919E-07	0,349574806

Bacteria	*Bacteroidetes*	*Bacteroidia*	*Bacteroidales*	*Prevotellaceae*	*Paraprevotella*	9,81604E-05	0,010713998	0,027890529
Bacteria	*Proteobacteria*	*Betaproteobacteria*	*Burkholderiales*	*Alcaligenaceae*	*Parapusillimonas*	0,000000398	0	0,285049407
Bacteria	*Bacteroidetes*	*Sphingobacteriia*	*Sphingobacteriales*	*Chitinophagaceae*	*Parasegetibacter*	0	2,91662E-07	0,349574806
Bacteria	*Firmicutes*	*Clostridia*	*Clostridiales*	*Lachnospiraceae*	*Parasporobacterium*	7,06532E-07	4,57716E-05	0,017081066
Bacteria	*Proteobacteria*	*Betaproteobacteria*	*Burkholderiales*	*Sutterellaceae*	*Parasutterella*	3,17413E-05	8,94758E-05	0,370027712
Bacteria	*Parcubacteria*	*Parcubacteria_genera_incertae_sedis*	*Parcubacteria_genera_incertae_sedis*	*Parcubacteria_genera_incertae_sedis*	*Parcubacteria_genera_incertae_sedis*	0	7,39733E-07	0,349574806
Bacteria	*Firmicutes*	*Clostridia*	*Clostridiales*	*Clostridiales_Incertae Sedis XI*	*Parvimonas*	8,1594E-06	7,30815E-06	0,79400268
Bacteria	*Proteobacteria*	*Gammaproteobacteria*	*Pasteurellales*	*Pasteurellaceae*	*Pasteurella*	0,000176255	0,000120868	0,602200006
Bacteria	*Firmicutes*	*Bacilli*	*Lactobacillales*	*Lactobacillaceae*	*Pediococcus*	0,000496375	6,58156E-06	0,055829295
Bacteria	*Bacteroidetes*	*Sphingobacteriia*	*Sphingobacteriales*	*Sphingobacteriaceae*	*Pedobacter*	2,58607E-06	4,62949E-07	0,844911363
Bacteria	*Proteobacteria*	*Alphaproteobacteria*	*Rhizobiales*	*Hyphomicrobiaceae*	*Pedomicrobium*	7,32089E-06	0	0,285049407
Bacteria	*Proteobacteria*	*Alphaproteobacteria*	*Rhizobiales*	*Hyphomicrobiaceae*	*Pelagibacterium*	0	3,29911E-06	0,349574806
Bacteria	*Proteobacteria*	*Betaproteobacteria*	*Burkholderiales*	*Comamonadaceae*	*Pelomonas*	0,000933318	9,65865E-06	0,000967429
Bacteria	*Firmicutes*	*Clostridia*	*Clostridiales*	*Syntrophomonadaceae*	*Pelospora*	0	0,000002502	0,17090352
Bacteria	*Firmicutes*	*Clostridia*	*Clostridiales*	*Peptococcaceae 1*	*Peptococcus*	2,92096E-05	0,000142396	0,06170669
Bacteria	*Firmicutes*	*Clostridia*	*Clostridiales*	*Clostridiales_Incertae Sedis XI*	*Peptoniphilus*	5,37567E-05	0,00012996	0,857714481
Bacteria	*Firmicutes*	*Clostridia*	*Clostridiales*	*Peptostreptococcaceae*	*Peptostreptococcaceae_incertae_sedis*	2,95543E-05	1,79951E-06	0,181320849
Bacteria	*Firmicutes*	*Clostridia*	*Clostridiales*	*Peptostreptococcaceae*	*Peptostreptococcus*	0,00012851	2,84327E-05	0,120232801

Bacteria	*Proteobacteria*	*Deltaproteobacteria*	*Bdellovibrionales*	*Bacteriovoracaceae*	*Peredibacter*	4,97513E-06	1,4996E-06	0,844911363
Bacteria	*Proteobacteria*	*Gammaproteobacteria*	*Pseudomonadales*	*Moraxellaceae*	*Perlucidibaca*	0	6,89814E-06	0,349574806
Bacteria	*Bacteroidetes*	*Bacteroidia*	*Bacteroidales*	*Porphyromonadaceae*	*Petrimonas*	0,000649814	6,53192E-05	0,945071382
Bacteria	*Firmicutes*	*Negativicutes*	*Selenomonadales*	*Acidaminococcaceae*	*Phascolarctobacterium*	0,000432971	0,030125145	0,003813532
Bacteria	*Proteobacteria*	*Deltaproteobacteria*	*Myxococcales*	*Phaselicystidaceae*	*Phaselicystis*	1,83022E-06	0	0,285049407
Bacteria	*Proteobacteria*	*Alphaproteobacteria*	*Rhizobiales*	*Phyllobacteriaceae*	*Phyllobacterium*	0	0,000059531	0,006111784
Bacteria	*Firmicutes*	*Bacilli*	*Lactobacillales*	*Enterococcaceae*	*Pilibacter*	0	2,99919E-07	0,349574806
Bacteria	*Bacteroidetes*	*Flavobacteriia*	*Flavobacteriales*	*Flavobacteriaceae*	*Planobacterium*	0,003220167	0,000760112	0,033894854
Bacteria	*Firmicutes*	*Bacilli*	*Bacillales*	*Planococcaceae*	*Planococcaceae_incertae_sedis*	7,94526E-05	2,28458E-05	0,198958931
Bacteria	*Firmicutes*	*Bacilli*	*Bacillales*	*Planococcaceae*	*Planomicrobium*	9,17128E-06	0	0,117601295
Bacteria	*Proteobacteria*	*Betaproteobacteria*	*Burkholderiales*	*Burkholderiaceae*	*Polynucleobacter*	0	2,42065E-06	0,17090352
Bacteria	*Bacteroidetes*	*Cytophagia*	*Cytophagales*	*Cytophagaceae*	*Pontibacter*	0	2,3333E-06	0,349574806
Bacteria	*Proteobacteria*	*Alphaproteobacteria*	*Sphingomonadales*	*Erythrobacteraceae*	*Porphyrobacter*	3,44809E-06	0	0,285049407
Bacteria	*Bacteroidetes*	*Bacteroidia*	*Bacteroidales*	*Porphyromonadaceae*	*Porphyromonas*	0,000737638	9,34212E-05	0,007719174
Bacteria	*Bacteroidetes*	*Bacteroidia*	*Bacteroidales*	*Prevotellaceae*	*Prevotella*	0,003662488	0,141538222	0,082579274
Bacteria	*Bacteroidetes*	*Bacteroidetes"_incertae_sedis*	*Prolixibacter*	*Prolixibacter*	*Prolixibacter*	0	4,42176E-07	0,349574806
Bacteria	*Actinobacteria*	*Actinobacteria*	*Actinomycetales*	*Propionibacteriaceae*	*Propionibacterium*	0,003445225	4,01923E-05	0,001145938
Bacteria	*Actinobacteria*	*Actinobacteria*	*Actinomycetales*	*Propionibacteriaceae*	*Propionicicella*	0,000000796	0,000023078	0,508110562

Bacteria	*Actinobacteria*	*Actinobacteria*	*Actinomycetales*	*Propionibacteriaceae*	*Propionicimonas*	0	4,39357E-06	0,17090352
Bacteria	*Fusobacteria*	*Fusobacteriia*	*Fusobacteriales*	*Fusobacteriaceae*	*Propionigenium*	5,72662E-05	9,29749E-06	0,234763663
Bacteria	*Actinobacteria*	*Actinobacteria*	*Actinomycetales*	*Propionibacteriaceae*	*Propionimicrobium*	0	9,89237E-07	0,349574806
Bacteria	*Firmicutes*	*Clostridia*	*Clostridiales*	*Clostridiaceae 1*	*Proteiniclasticum*	2,18624E-05	1,97947E-05	0,562548843
Bacteria	*Bacteroidetes*	*Bacteroidia*	*Bacteroidales*	*Porphyromonadaceae*	*Proteiniphilum*	8,2193E-06	5,99838E-06	0,562548843
Bacteria	*Firmicutes*	*Clostridia*	*Clostridiales*	*Peptostreptococcaceae*	*Proteocatella*	2,01371E-05	0,00002846	0,881930721
Bacteria	*Proteobacteria*	*Gammaproteobacteria*	*Enterobacteriales*	*Enterobacteriaceae*	*Proteus*	4,82203E-05	0	0,117601295
Bacteria	*Proteobacteria*	*Gammaproteobacteria*	*Enterobacteriales*	*Enterobacteriaceae*	*Providencia*	0,000115224	0	0,285049407
Bacteria	*Firmicutes*	*Clostridia*	*Clostridiales*	*Lachnospiraceae*	*Pseudobutyrivibrio*	0	4,55022E-05	0,015891197
Bacteria	*Proteobacteria*	*Alphaproteobacteria*	*Rhizobiales*	*Brucellaceae*	*Pseudochrobactrum*	4,17911E-06	1,28916E-06	0,740726595
Bacteria	*Actinobacteria*	*Actinobacteria*	*Actinomycetales*	*Microbacteriaceae*	*Pseudoclavibacter*	0,000001061	1,4211E-06	0,562548843
Bacteria	*Firmicutes*	*Clostridia*	*Clostridiales*	*Ruminococcaceae*	*Pseudoflavonifractor*	0,000168818	0,001182566	0,164537456
Bacteria	*Proteobacteria*	*Gammaproteobacteria*	*Pseudomonadales*	*Pseudomonadaceae*	*Pseudomonas*	0,003370249	1,76785E-05	0,001181672
Bacteria	*Actinobacteria*	*Actinobacteria*	*Actinomycetales*	*Pseudonocardiaceae*	*Pseudonocardia*	8,45928E-06	0	0,285049407
Bacteria	*Firmicutes*	*Clostridia*	*Clostridiales*	*Eubacteriaceae*	*Pseudoramibacter*	0	2,57831E-06	0,17090352
Bacteria	*Bacteroidetes*	*Sphingobacteriia*	*Sphingobacteriales*	*Sphingobacteriaceae*	*Pseudosphingobacterium*	0	0,000005459	0,17090352
Bacteria	*Proteobacteria*	*Alphaproteobacteria*	*Rhodobacterales*	*Rhodobacteraceae*	*Pseudovibrio*	1,18964E-05	0	0,285049407
Bacteria	*Proteobacteria*	*Gammaproteobacteria*	*Xanthomonadales*	*Xanthomonadaceae*	*Pseudoxanthomonas*	0,000169018	0	0,047105791

Bacteria	*Firmicutes*	*Bacilli*	*Bacillales*	*Planococcaceae*	*Psychrobacillus*	3,06667E-06	5,26166E-06	0,619656703
Bacteria	*Proteobacteria*	*Gammaproteobacteria*	*Pseudomonadales*	*Moraxellaceae*	*Psychrobacter*	0,002760908	0,055317475	0,562833754
Bacteria	*Firmicutes*	*Negativicutes*	*Selenomonadales*	*Veillonellaceae*	*Psychrosinus*	0	4,73698E-07	0,349574806
Bacteria	*Proteobacteria*	*Betaproteobacteria*	*Burkholderiales*	*Alcaligenaceae*	*Pusillimonas*	1,06968E-05	0	0,117601295
Bacteria	*Synergistetes*	*Synergistia*	*Synergistales*	*Synergistaceae*	*Pyramidobacter*	7,3796E-06	6,91294E-05	0,090155978
Bacteria	*Proteobacteria*	*Betaproteobacteria*	*Burkholderiales*	*Burkholderiaceae*	*Ralstonia*	0	9,57264E-06	0,17090352
Bacteria	*Actinobacteria*	*Actinobacteria*	*Actinomycetales*	*Micrococcaceae*	*Renibacterium*	0,000230735	0	0,000440292
Bacteria	*Proteobacteria*	*Gammaproteobacteria*	*Chromatiales*	*Chromatiaceae*	*Rheinheimera*	0,000166427	7,19806E-06	0,084991283
Bacteria	*Proteobacteria*	*Gammaproteobacteria*	*Pseudomonadales*	*Pseudomonadaceae*	*Rhizobacter*	1,41306E-06	0	0,285049407
Bacteria	*Proteobacteria*	*Alphaproteobacteria*	*Rhizobiales*	*Rhizobiaceae*	*Rhizobium*	0,000299533	2,23507E-05	0,010825507
Bacteria	*Proteobacteria*	*Alphaproteobacteria*	*Rhodobacterales*	*Rhodobacteraceae*	*Rhodobacter*	1,01337E-05	1,16968E-05	0,147940923
Bacteria	*Actinobacteria*	*Actinobacteria*	*Actinomycetales*	*Nocardiaceae*	*Rhodococcus*	0,000397734	0	0,000440292
Bacteria	*Bacteroidetes*	*Flavobacteriia*	*Flavobacteriales*	*Flavobacteriaceae*	*Riemerella*	2,20421E-05	3,16277E-05	0,72367361
Bacteria	*Firmicutes*	*Clostridia*	*Clostridiales*	*Lachnospiraceae*	*Robinsoniella*	1,58104E-05	1,43536E-05	0,083081187
Bacteria	*Firmicutes*	*Clostridia*	*Clostridiales*	*Lachnospiraceae*	*Roseburia*	0,000198163	0,02474264	0,005478553
Bacteria	*Proteobacteria*	*Alphaproteobacteria*	*Rhodobacterales*	*Rhodobacteraceae*	*Roseovarius*	7,06532E-07	0	0,285049407
Bacteria	*Actinobacteria*	*Actinobacteria*	*Actinomycetales*	*Micrococcaceae*	*Rothia*	0,005578648	0,014062349	0,816961322
Bacteria	*Proteobacteria*	*Alphaproteobacteria*	*Rhodobacterales*	*Rhodobacteraceae*	*Rubellimicrobium*	1,02082E-06	0,000002625	1

Bacteria	*Actinobacteria*	*Actinobacteria*	*Rubrobacterales*	*Rubrobacteraceae*	*Rubrobacter*	1,03443E-05	0	0,285049407
Bacteria	*Proteobacteria*	*Gammaproteobacteria*	*Aeromonadales*	*Succinivibrionaceae*	*Ruminobacter*	3,18408E-06	6,11115E-05	0,234763663
Bacteria	*Firmicutes*	*Clostridia*	*Clostridiales*	*Ruminococcaceae*	*Ruminococcus*	0,000655817	0,004439634	0,105192505
Bacteria	*Firmicutes*	*Clostridia*	*Clostridiales*	*Lachnospiraceae*	*Ruminococcus2*	0,000146657	0,000738994	0,020637558
Bacteria	*Candidatus Saccharibacteria*	*Saccharibacteria_genera_incertae_sedis*	*Saccharibacteria_genera_incertae_sedis*	*Saccharibacteria_genera_incertae_sedis*	*Saccharibacteria_genera_incertae_sedis*	0,000213031	0,000629193	0,562833754
Bacteria	*Firmicutes*	*Clostridia*	*Clostridiales*	*Ruminococcaceae*	*Saccharofermentans*	0,000000597	6,30203E-05	0,003414791
Bacteria	*Actinobacteria*	*Actinobacteria*	*Actinomycetales*	*Pseudonocardiaceae*	*Saccharomonospora*	0	8,99757E-07	0,349574806
Bacteria	*Firmicutes*	*Bacilli*	*Bacillales*	*Staphylococcaceae*	*Salinicoccus*	1,48372E-05	3,65635E-06	0,740726595
Bacteria	*Bacteroidetes*	*Flavobacteriia*	*Flavobacteriales*	*Flavobacteriaceae*	*Salinimicrobium*	3,44809E-05	0	0,285049407
Bacteria	*Proteobacteria*	*Gammaproteobacteria*	*Enterobacteriales*	*Enterobacteriaceae*	*Salmonella*	0,000012156	2,61091E-05	0,038099036
Bacteria	*Firmicutes*	*Clostridia*	*Clostridiales*	*Clostridiaceae 1*	*Sarcina*	5,09529E-06	0,00012611	0,063128008
Bacteria	*Proteobacteria*	*Betaproteobacteria*	*Burkholderiales*	*Comamonadaceae*	*Schlegelella*	0,000014486	0	0,047105791
Bacteria	*Firmicutes*	*Negativicutes*	*Selenomonadales*	*Veillonellaceae*	*Schwartzia*	2,11959E-06	0,003594695	0,002807439
Bacteria	*Firmicutes*	*Clostridia*	*Clostridiales*	*Clostridiales_Incertae Sedis XI*	*Sedimentibacter*	3,98011E-06	0	0,285049407
Bacteria	*Firmicutes*	*Negativicutes*	*Selenomonadales*	*Veillonellaceae*	*Selenomonas*	6,91351E-05	0,010997337	0,027890529
Bacteria	*Actinobacteria*	*Actinobacteria*	*Actinomycetales*	*Beutenbergiaceae*	*Serinibacter*	0	0,000129865	0,349574806
Bacteria	*Proteobacteria*	*Gammaproteobacteria*	*Pseudomonadales*	*Pseudomonadaceae*	*Serpens*	3,61881E-05	2,99919E-07	0,045714365
Bacteria	*Proteobacteria*	*Gammaproteobacteria*	*Enterobacteriales*	*Enterobacteriaceae*	*Serratia*	0	1,45831E-06	0,349574806

Bacteria	*Firmicutes*	*Erysipelotrichia*	*Erysipelotrichales*	*Erysipelotrichaceae*	*Sharpea*	6,03417E-06	0	0,285049407
Bacteria	*Proteobacteria*	*Gammaproteobacteria*	*Alteromonadales*	*Shewanellaceae*	*Shewanella*	1,68598E-05	6,89814E-06	0,147940923
Bacteria	*Proteobacteria*	*Betaproteobacteria*	*Rhodocyclales*	*Rhodocyclaceae*	*Shinella*	1,18381E-05	2,99919E-07	0,137485208
Bacteria	*Firmicutes*	*Clostridia*	*Clostridiales*	*Lachnospiraceae*	*Shuttleworthia*	0	5,07992E-06	0,082355525
Bacteria	*Proteobacteria*	*Betaproteobacteria*	*Burkholderiales*	*Comamonadaceae*	*Simplicispira*	3,18408E-06	5,99838E-06	1
Bacteria	*Proteobacteria*	*Alphaproteobacteria*	*Rhodospirillales*	*Rhodospirillaceae*	*Skermanella*	0,00003462	0	0,285049407
Bacteria	*Actinobacteria*	*Actinobacteria*	*Coriobacteriales*	*Coriobacteriaceae*	*Slackia*	4,94572E-06	1,05067E-06	0,740726595
Bacteria	*Fusobacteria*	*Fusobacteriia*	*Fusobacteriales*	*Leptotrichiaceae*	*Sneathia*	0	1,80089E-06	0,17090352
Bacteria	*Actinobacteria*	*Actinobacteria*	*Solirubrobacterales*	*Solirubrobacteraceae*	*Solirubrobacter*	7,75821E-06	0	0,285049407
Bacteria	*Bacteroidetes*	*Flavobacteriia*	*Flavobacteriales*	*Flavobacteriaceae*	*Soonwooa*	0,000204741	3,39191E-05	0,801776827
Bacteria	*Chloroflexi*	*Thermomicrobia*	*Sphaerobacterales*	*Sphaerobacteraceae*	*Sphaerobacter*	4,11211E-06	1,79951E-06	0,456570763
Bacteria	*Spirochaetes*	*Spirochaetia*	*Spirochaetales*	*Spirochaetaceae*	*Sphaerochaeta*	0,000086758	0,001498719	0,04893855
Bacteria	*Bacteroidetes*	*Sphingobacteriia*	*Sphingobacteriales*	*Sphingobacteriaceae*	*Sphingobacterium*	0,000394893	3,94684E-05	0,191694602
Bacteria	*Proteobacteria*	*Alphaproteobacteria*	*Sphingomonadales*	*Sphingomonadaceae*	*Sphingobium*	3,11701E-05	4,19887E-06	0,181320849
Bacteria	*Proteobacteria*	*Alphaproteobacteria*	*Sphingomonadales*	*Sphingomonadaceae*	*Sphingomonas*	0,000168477	0,000259155	0,417886964
Bacteria	*Proteobacteria*	*Alphaproteobacteria*	*Sphingomonadales*	*Sphingomonadaceae*	*Sphingopyxis*	4,97513E-06	0	0,285049407
Bacteria	*Proteobacteria*	*Alphaproteobacteria*	*Sphingomonadales*	*Sphingomonadaceae*	*Sphingosinicella*	6,57144E-06	0	0,285049407
Bacteria	*Spirochaetes*	*Spirochaetia*	*Spirochaetales*	*Spirochaetaceae*	*Spirochaeta*	0	4,42176E-07	0,349574806

Bacteria	*Firmicutes*	*Clostridia*	*Clostridiales*	*Peptostreptococcaceae*	*Sporacetigenium*	0,000206771	0,000127091	0,105192505
Bacteria	*Firmicutes*	*Clostridia*	*Clostridiales*	*Ruminococcaceae*	*Sporobacter*	0,000108698	0,001201708	0,010896354
Bacteria	*Firmicutes*	*Bacilli*	*Bacillales*	*Sporolactobacillaceae*	*Sporolactobacillaceae_incertae_sedis*	0	4,62949E-07	0,349574806
Bacteria	*Firmicutes*	*Bacilli*	*Bacillales*	*Planococcaceae*	*Sporosarcina*	7,3632E-06	0	0,285049407
Bacteria	*SR1*	*SR1_genera_incertae_sedis*	*SR1_genera_incertae_sedis*	*SR1_genera_incertae_sedis*	*SR1_genera_incertae_sedis*	8,8471E-06	2,39996E-05	0,372857936
Bacteria	*Proteobacteria*	*Alphaproteobacteria*	*Sphingomonadales*	*Sphingomonadaceae*	*Stakelama*	7,06532E-07	0	0,285049407
Bacteria	*Firmicutes*	*Bacilli*	*Bacillales*	*Staphylococcaceae*	*Staphylococcus*	0,014930157	0,000286782	0,001713496
Bacteria	*Proteobacteria*	*Gammaproteobacteria*	*Xanthomonadales*	*Xanthomonadaceae*	*Stenotrophomonas*	0,00044035	1,64699E-05	0,000967429
Bacteria	*Fusobacteria*	*Fusobacteriia*	*Fusobacteriales*	*Leptotrichiaceae*	*Streptobacillus*	8,87339E-05	2,39935E-06	0,362848315
Bacteria	*Firmicutes*	*Bacilli*	*Lactobacillales*	*Streptococcaceae*	*Streptococcus*	0,015834647	0,010277126	0,417886964
Bacteria	*Actinobacteria*	*Actinobacteria*	*Actinomycetales*	*Streptomycetaceae*	*Streptomyces*	1,73871E-05	0	0,285049407
Bacteria	*Cyanobacteria/Chloroplast*	*Chloroplast*	*Chloroplast*	*Chloroplast*	*Streptophyta*	0,001530934	0,000120038	0,002621822
Bacteria	*Verrucomicrobia*	*Subdivision5*	*Subdivision5_genera_incertae_sedis*	*Subdivision5_genera_incertae_sedis*	*Subdivision5_genera_incertae_sedis*	0,000188272	0,00027059	0,48745329
Bacteria	*Firmicutes*	*Clostridia*	*Clostridiales*	*Ruminococcaceae*	*Subdoligranulum*	0,000000862	1,87995E-05	0,050186187
Bacteria	*Firmicutes*	*Negativicutes*	*Selenomonadales*	*Acidaminococcaceae*	*Succiniclasticum*	0	4,42176E-07	0,349574806
Bacteria	*Firmicutes*	*Negativicutes*	*Selenomonadales*	*Acidaminococcaceae*	*Succinispira*	0	4,42176E-07	0,349574806
Bacteria	*Proteobacteria*	*Gammaproteobacteria*	*Aeromonadales*	*Succinivibrionaceae*	*Succinivibrio*	0,000123977	0,010582261	0,037242545
Bacteria	*Proteobacteria*	*Betaproteobacteria*	*Burkholderiales*	*Sutterellaceae*	*Sutterella*	9,13008E-06	0,000271033	0,031443735

Bacteria	*Proteobacteria*	*Gammaproteobacteria*	*Cardiobacteriales*	*Cardiobacteriaceae*	*Suttonella*	0	3,70778E-06	0,037588881
Bacteria	*Synergistetes*	*Synergistia*	*Synergistales*	*Synergistaceae*	*Synergistes*	2,16189E-05	2,45508E-05	0,881930721
Bacteria	*Firmicutes*	*Clostridia*	*Clostridiales*	*Lachnospiraceae*	*Syntrophococcus*	6,80546E-06	6,51954E-06	0,457717434
Bacteria	*Bacteroidetes*	*Bacteroidia*	*Bacteroidales*	*Porphyromonadaceae*	*Tannerella*	6,61888E-05	0,000151869	0,816315804
Bacteria	*Proteobacteria*	*Betaproteobacteria*	*Burkholderiales*	*Burkholderiales_incertae_sedis*	*Tepidimonas*	4,01889E-05	0	0,047105791
Bacteria	*Actinobacteria*	*Actinobacteria*	*Actinomycetales*	*Propionibacteriaceae*	*Tessaracoccus*	1,10684E-05	0,000240535	0,298500252
Bacteria	*Firmicutes*	*Bacilli*	*Lactobacillales*	*Enterococcaceae*	*Tetragenococcus*	0	3,0307E-06	0,082355525
Bacteria	*Proteobacteria*	*Gammaproteobacteria*	*Oceanospirillales*	*Oceanospirillaceae*	*Thalassolituus*	3,98011E-06	0	0,285049407
Bacteria	*Proteobacteria*	*Betaproteobacteria*	*Rhodocyclales*	*Rhodocyclaceae*	*Thauera*	2,38806E-06	5,99838E-07	0,844911363
Bacteria	*Firmicutes*	*Bacilli*	*Bacillales*	*Bacillales_Incertae Sedis X*	*Thermicanus*	8,62024E-06	0	0,285049407
Archaea	*Euryarchaeota*	*Thermoplasmata*	*Thermoplasmatales*	*Thermoplasmatales_incertae_sedis*	*Thermogymnomonas*	9,75978E-06	1,63439E-05	0,679325522
Bacteria	*Actinobacteria*	*Thermoleophilia*	*Thermoleophilales*	*Thermoleophilaceae*	*Thermoleophilum*	1,28116E-05	0	0,285049407
Bacteria	*Proteobacteria*	*Gammaproteobacteria*	*Xanthomonadales*	*Xanthomonadaceae*	*Thermomonas*	1,07118E-05	0	0,047105791
Bacteria	*Proteobacteria*	*Gammaproteobacteria*	*Thiotrichales*	*Thiotrichaceae*	*Thiothrix*	0	7,7979E-06	0,349574806
Bacteria	*Firmicutes*	*Clostridia*	*Clostridiales*	*Clostridiales_Incertae Sedis XI*	*Tissierella*	3,08859E-05	2,39935E-06	0,137485208
Bacteria	*Proteobacteria*	*Gammaproteobacteria*	*Aeromonadales*	*Aeromonadaceae*	*Tolumonas*	0,000000199	4,73698E-07	1
Bacteria	*Actinobacteria*	*Actinobacteria*	*Actinomycetales*	*Corynebacterineae_incertae_sedis*	*Tomitella*	2,72976E-06	0	0,047105791
Bacteria	*Spirochaetes*	*Spirochaetia*	*Spirochaetales*	*Spirochaetaceae*	*Treponema*	0,000471774	0,026672722	0,105192505

Bacteria	*Firmicutes*	*Bacilli*	*Lactobacillales*	*Carnobacteriaceae*	*Trichococcus*	0,000195363	0,000035615	0,188593442
Bacteria	*Deinococcus-Thermus*	*Deinococci*	*Deinococcales*	*Trueperaceae*	*Truepera*	8,62024E-06	0	0,285049407
Bacteria	*Actinobacteria*	*Actinobacteria*	*Actinomycetales*	*Actinomycetaceae*	*Trueperella*	0	5,92295E-05	0,037588881
Bacteria	*Actinobacteria*	*Actinobacteria*	*Actinomycetales*	*Corynebacteriaceae*	*Turicella*	1,33769E-06	0,000009808	0,191694602
Bacteria	*Firmicutes*	*Erysipelotrichia*	*Erysipelotrichales*	*Erysipelotrichaceae*	*Turicibacter*	5,83048E-05	0,000405155	0,027750993
Bacteria	*Proteobacteria*	*Betaproteobacteria*	*Burkholderiales*	*Oxalobacteraceae*	*Undibacterium*	0,000635577	5,84609E-06	0,000967429
Bacteria	*Firmicutes*	*Bacilli*	*Bacillales*	*Planococcaceae*	*Ureibacillus*	0	8,84352E-07	0,349574806
Bacteria	*Proteobacteria*	*Betaproteobacteria*	*Neisseriales*	*Neisseriaceae*	*Uruburuella*	1,0986E-06	2,84706E-05	0,315285648
Bacteria	*Firmicutes*	*Bacilli*	*Lactobacillales*	*Enterococcaceae*	*Vagococcus*	0,000100959	5,82023E-05	0,902908911
Bacteria	*Proteobacteria*	*Deltaproteobacteria*	*Bdellovibrionales*	*Bdellovibrionaceae*	*Vampirovibrio*	0,000189007	0,000498828	0,027890529
Bacteria	*Proteobacteria*	*Betaproteobacteria*	*Burkholderiales*	*Comamonadaceae*	*Variovorax*	2,18906E-06	1,47947E-06	0,844911363
Bacteria	*Firmicutes*	*Negativicutes*	*Selenomonadales*	*Veillonellaceae*	*Veillonella*	0,000106465	0,000252748	0,131079232
Bacteria	*Proteobacteria*	*Gammaproteobacteria*	*Vibrionales*	*Vibrionaceae*	*Vibrio*	0,000148541	6,3144E-06	0,945071382
Bacteria	*Lentisphaerae*	*Lentisphaeria*	*Victivallales*	*Victivallaceae*	*Victivallis*	0,00000911	3,94448E-05	0,089648055
Bacteria	*Proteobacteria*	*Betaproteobacteria*	*Neisseriales*	*Neisseriaceae*	*Vitreoscilla*	3,83586E-05	5,08825E-06	0,79400268
Bacteria	*Bacteroidetes*	*Flavobacteriia*	*Flavobacteriales*	*Flavobacteriaceae*	*Wautersiella*	0,001665323	4,82547E-05	0,11277615
Bacteria	*Bacteroidetes*	*Flavobacteriia*	*Flavobacteriales*	*Flavobacteriaceae*	*Weeksella*	4,81318E-05	0	0,047105791
Bacteria	*Firmicutes*	*Bacilli*	*Lactobacillales*	*Leuconostocaceae*	*Weissella*	0,001485887	0,000340158	0,164537456

Bacteria	*Actinobacteria*	*Actinobacteria*	*Actinomycetales*	*Nocardiaceae*	*Williamsia*	0,000000862	0	0,285049407
Bacteria	*Proteobacteria*	*Gammaproteobacteria*	*Xanthomonadales*	*Xanthomonadaceae*	*Wohlfahrtiimonas*	0,000629628	7,1974E-06	0,601508134
Bacteria	*Proteobacteria*	*Epsilonproteobacteria*	*Campylobacterales*	*Helicobacteraceae*	*Wolinella*	0	2,21088E-06	0,349574806
Bacteria	*Proteobacteria*	*Gammaproteobacteria*	*Xanthomonadales*	*Xanthomonadaceae*	*Xanthomonas*	1,92173E-05	0	0,285049407
Bacteria	*Bacteroidetes*	*Bacteroidia*	*Bacteroidales*	*Prevotellaceae*	*Xylanibacter*	0	2,31948E-05	0,015891197
Bacteria	*Proteobacteria*	*Gammaproteobacteria*	*Xanthomonadales*	*Xanthomonadaceae*	*Xylella*	1,50043E-06	0	0,117601295
Bacteria	*Actinobacteria*	*Actinobacteria*	*Actinomycetales*	*Micrococcaceae*	*Yaniella*	5,61024E-06	5,69846E-06	0,562548843
Bacteria	*Proteobacteria*	*Gammaproteobacteria*	*Enterobacteriales*	*Enterobacteriaceae*	*Yokenella*	0	2,6946E-06	0,17090352
Bacteria	*Proteobacteria*	*Gammaproteobacteria*	*Aeromonadales*	*Aeromonadaceae*	*Zobellella*	1,57214E-05	0	0,285049407
Bacteria	*Proteobacteria*	*Betaproteobacteria*	*Rhodocyclales*	*Rhodocyclaceae*	*Zoogloea*	0	0,000003599	0,349574806

Additional file 1B. Frequencies of OTUs assigned at genus level from farm MC at the two sampling times (MC1 and MC2).

Domain	Phylum	Class	Order	Family	Genus	Frequency		FDR_P
						MC1	MC2	
Bacteria	*Firmicutes*	*Bacilli*	*Lactobacillales*	*Aerococcaceae*	*Abiotrophia*	3,85279E-06	0	0,240955
Bacteria	*Firmicutes*	*Clostridia*	*Clostridiales*	*Ruminococcaceae*	*Acetanaerobacterium*	0,000249138	0,001487629	0,003837
Bacteria	*Firmicutes*	*Clostridia*	*Clostridiales*	*Lachnospiraceae*	*Acetitomaculum*	3,86726E-05	2,52909E-06	0,562711
Bacteria	*Firmicutes*	*Clostridia*	*Clostridiales*	*Ruminococcaceae*	*Acetivibrio*	8,99057E-05	9,32725E-05	0,559796
Bacteria	*Firmicutes*	*Clostridia*	*Clostridiales*	*Peptostreptococcaceae*	*Acetoanaerobium*	0	0,000042065	0,393769
Bacteria	*Proteobacteria*	*Alphaproteobacteria*	*Rhodospirillales*	*Acetobacteraceae*	*Acetobacter*	0	4,22886E-07	0,393769
Bacteria	*Proteobacteria*	*Betaproteobacteria*	*Burkholderiales*	*Alcaligenaceae*	*Achromobacter*	0	1,16274E-05	0,215311
Bacteria	*Firmicutes*	*Clostridia*	*Clostridiales*	*Clostridiales_Incertae Sedis XII*	*Acidaminobacter*	0	0,00000021	0,393769
Bacteria	*Firmicutes*	*Negativicutes*	*Selenomonadales*	*Acidaminococcaceae*	*Acidaminococcus*	0,000048249	0,00179064	0,002533
Bacteria	*Proteobacteria*	*Betaproteobacteria*	*Burkholderiales*	*Comamonadaceae*	*Acidovorax*	5,40671E-06	9,82409E-06	0,745201
Bacteria	*Proteobacteria*	*Gammaproteobacteria*	*Pseudomonadales*	*Moraxellaceae*	*Acinetobacter*	0,002451465	0,003426505	0,08278
Bacteria	*Proteobacteria*	*Gammaproteobacteria*	*Pasteurellales*	*Pasteurellaceae*	*Actinobacillus*	0,010895738	0,023693333	0,1372
Bacteria	*Actinobacteria*	*Actinobacteria*	*Actinomycetales*	*Actinomycetaceae*	*Actinobaculum*	2,16269E-06	0	0,240955
Bacteria	*Actinobacteria*	*Actinobacteria*	*Actinomycetales*	*Actinomycetaceae*	*Actinomyces*	1,82398E-05	1,21859E-05	0,256447
Bacteria	*Actinobacteria*	*Actinobacteria*	*Actinomycetales*	*Pseudonocardiaceae*	*Actinomycetospora*	9,84399E-07	0	0,240955
Bacteria	*Actinobacteria*	*Actinobacteria*	*Coriobacteriales*	*Coriobacteriaceae*	*Adlercreutzia*	0	0,00000021	0,393769
Bacteria	*Proteobacteria*	*Betaproteobacteria*	*Burkholderiales*	*Alcaligenaceae*	*Advenella*	8,62305E-05	1,29879E-05	0,920279

Bacteria	*Firmicutes*	*Bacilli*	*Lactobacillales*	*Aerococcaceae*	*Aerococcus*	0,000830181	0,001284797	0,317468
Bacteria	*Actinobacteria*	*Actinobacteria*	*Actinomycetales*	*Nocardioidaceae*	*Aeromicrobium*	0	0,000003486	0,393769
Bacteria	*Proteobacteria*	*Gammaproteobacteria*	*Aeromonadales*	*Aeromonadaceae*	*Aeromonas*	0,00023934	3,85947E-05	0,020263
Bacteria	*Proteobacteria*	*Alphaproteobacteria*	*Rhizobiales*	*Bradyrhizobiaceae*	*Afipia*	4,40319E-06	0,010893441	0,000517
Bacteria	*Actinobacteria*	*Actinobacteria*	*Actinomycetales*	*Microbacteriaceae*	*Agrococcus*	3,95305E-06	1,25915E-05	0,831292
Bacteria	*Verrucomicrobia*	*Verrucomicrobiae*	*Verrucomicrobiales*	*Verrucomicrobiaceae*	*Akkermansia*	0,000529487	0,000539944	0,057543
Bacteria	*Proteobacteria*	*Betaproteobacteria*	*Burkholderiales*	*Alcaligenaceae*	*Alcaligenes*	1,88601E-05	5,39894E-06	0,562711
Bacteria	*Bacteroidetes*	*Bacteroidia*	*Bacteroidales*	*Rikenellaceae*	*Alistipes*	0,01242031	0,000058865	0,000247
Bacteria	*Firmicutes*	*Clostridia*	*Clostridiales*	*Eubacteriaceae*	*Alkalibacter*	6,60944E-05	0	0,240955
Bacteria	*Bacteroidetes*	*Bacteroidia*	*Bacteroidales*	*Marinilabiliaceae*	*Alkalitalea*	0	1,86309E-06	0,215311
Bacteria	*Proteobacteria*	*Gammaproteobacteria*	*Pseudomonadales*	*Moraxellaceae*	*Alkanindiges*	0,000098404	2,19089E-07	0,009265
Bacteria	*Firmicutes*	*Negativicutes*	*Selenomonadales*	*Veillonellaceae*	*Allisonella*	3,13748E-05	0,00035201	0,027385
Bacteria	*Firmicutes*	*Erysipelotrichia*	*Erysipelotrichales*	*Erysipelotrichaceae*	*Allobaculum*	0,000056476	3,68379E-07	0,009265
Bacteria	*Firmicutes*	*Bacilli*	*Lactobacillales*	*Carnobacteriaceae*	*Alloiococcus*	6,65059E-06	6,34329E-07	0,756733
Bacteria	*Bacteroidetes*	*Bacteroidia*	*Bacteroidales*	*Prevotellaceae*	*Alloprevotella*	0,004440881	0,01635263	0,020776
Bacteria	*Proteobacteria*	*Alphaproteobacteria*	*Sphingomonadales*	*Erythrobacteraceae*	*Altererythrobacter*	1,94395E-05	0	0,240955
Bacteria	*Proteobacteria*	*Betaproteobacteria*	*Neisseriales*	*Neisseriaceae*	*Alysiella*	1,46111E-05	3,68014E-05	0,76399
Bacteria	*Firmicutes*	*Clostridia*	*Clostridiales*	*Clostridiaceae 1*	*Anaerobacter*	4,93842E-06	3,23939E-05	0,010059
Bacteria	*Proteobacteria*	*Gammaproteobacteria*	*Aeromonadales*	*Succinivibrionaceae*	*Anaerobiospirillum*	0,000115705	1,97484E-05	0,920279

Bacteria	*Firmicutes*	*Clostridia*	*Clostridiales*	*Clostridiales_Incertae Sedis XI*	*Anaerococcus*	0,000733188	1,45974E-05	0,001292
Bacteria	*Firmicutes*	*Clostridia*	*Clostridiales*	*Ruminococcaceae*	*Anaerofilum*	1,55813E-05	1,81807E-05	0,35801
Bacteria	*Firmicutes*	*Clostridia*	*Clostridiales*	*Eubacteriaceae*	*Anaerofustis*	0	2,20888E-05	0,063994
Bacteria	*Firmicutes*	*Negativicutes*	*Selenomonadales*	*Veillonellaceae*	*Anaeroglobus*	0	8,28228E-06	0,063994
Bacteria	*Bacteroidetes*	*Bacteroidia*	*Bacteroidales*	*Marinilabiaceae*	*Anaerophaga*	0,000416787	0,00383883	0,038989
Bacteria	*Tenericutes*	*Mollicutes*	*Anaeroplasmatales*	*Anaeroplasmataceae*	*Anaeroplasma*	0,001608723	0,000501912	0,006432
Bacteria	*Bacteroidetes*	*Bacteroidia*	*Bacteroidales*	*Bacteroidaceae*	*Anaerorhabdus*	0	0,000019079	0,063994
Bacteria	*Firmicutes*	*Negativicutes*	*Selenomonadales*	*Veillonellaceae*	*Anaerosinus*	0	5,22264E-05	0,393769
Bacteria	*Firmicutes*	*Clostridia*	*Clostridiales*	*Incertae Sedis XI*	*Anaerosphaera*	0	1,59314E-06	0,393769
Bacteria	*Firmicutes*	*Clostridia*	*Clostridiales*	*Clostridiaceae 1*	*Anaerosporobacter*	0,000269978	2,90102E-05	0,080216
Bacteria	*Firmicutes*	*Clostridia*	*Clostridiales*	*Lachnospiraceae*	*Anaerostipes*	7,86722E-05	6,79029E-05	0,1625
Bacteria	*Firmicutes*	*Clostridia*	*Clostridiales*	*Ruminococcaceae*	*Anaerotruncus*	6,22565E-05	3,36816E-05	0,617189
Bacteria	*Firmicutes*	*Negativicutes*	*Selenomonadales*	*Veillonellaceae*	*Anaerovibrio*	0,00047358	0,010889484	0,010475
Bacteria	*Firmicutes*	*Clostridia*	*Clostridiales*	*Clostridiales_Incertae Sedis XIII*	*Anaerovorax*	8,78456E-05	0,000524325	0,002209
Bacteria	*Firmicutes*	*Bacilli*	*Bacillales*	*Bacillaceae 1*	*Anoxybacillus*	0,000013964	2,91599E-05	0,831292
Bacteria	*Proteobacteria*	*Betaproteobacteria*	*Burkholderiales*	*Burkholderiales_incertae_sedis*	*Aquabacterium*	0,000004388	0,000022529	0,417741
Bacteria	*Actinobacteria*	*Actinobacteria*	*Actinomycetales*	*Actinomycetaceae*	*Arcanobacterium*	9,85273E-06	4,87782E-06	0,728372
Bacteria	*Proteobacteria*	*Epsilonproteobacteria*	*Campylobacterales*	*Campylobacteraceae*	*Arcobacter*	7,81105E-05	0,000147272	0,41505

Bacteria	*Bacteroidetes*	*Sphingobacteriia*	*Sphingobacteriales*	*Sphingobacteriaceae*	*Arcticibacter*	0	8,76225 E-06	0,393769
Bacteria	*Armatimonadetes*	*Armatimonadetes_gp5*	*Armatimonadetes_gp5*	*Armatimonadetes_gp5*	*Armatimonadetes_gp5*	0	3,97513 E-05	0,393769
Bacteria	*Actinobacteria*	*Actinobacteria*	*Actinomycetales*	*Micrococcaceae*	*Arthrobacter*	0,00040690 1	0,00157 1564	0,185676
Bacteria	*Tenericutes*	*Mollicutes*	*Anaeroplasmatales*	*Anaeroplasmataceae*	*Asteroleplasma*	8,72316E-05	0,00054 7879	0,013355
Bacteria	*Proteobacteria*	*Alphaproteobacteria*	*Caulobacterales*	*Caulobacteraceae*	*Asticcacaulis*	0	1,68658 E-06	0,11879
Bacteria	*Firmicutes*	*Bacilli*	*Lactobacillales*	*Carnobacteriaceae*	*Atopobacter*	6,18883E-06	0,00000 8138	0,73845
Bacteria	*Actinobacteria*	*Actinobacteria*	*Coriobacteriales*	*Coriobacteriaceae*	*Atopobium*	4,43372E-06	5,80171 E-07	0,756733
Bacteria	*Firmicutes*	*Bacilli*	*Lactobacillales*	*Carnobacteriaceae*	*Atopostipes*	1,67642E-05	1,79473 E-05	0,816905
Bacteria	*Proteobacteria*	*Alphaproteobacteria*	*Rhizobiales*	*Aurantimonadaceae*	*Aurantimonas*	3,05164E-05	0	0,240955
Bacteria	*Proteobacteria*	*Gammaproteobacteria*	*Pseudomonadales*	*Pseudomonadaceae*	*Azorhizophilus*	1,9688E-06	0	0,240955
Bacteria	*Proteobacteria*	*Betaproteobacteria*	*Rhodocyclales*	*Rhodocyclaceae*	*Azospira*	0	1,89794 E-05	0,393769
Bacteria	*Cyanobacteria/Chl oroplast*	*Chloroplast*	*Chloroplast*	*Chloroplast*	*Bacillariophyta*	8,55676E-05	0	0,240955
Bacteria	*Firmicutes*	*Bacilli*	*Bacillales*	*Bacillaceae 1*	*Bacillus*	8,69667E-05	0,00033 6086	0,619679
Bacteria	*Bacteroidetes*	*Bacteroidia*	*Bacteroidales*	*Bacteroidaceae*	*Bacteroides*	0,01694078 3	0,00365 948	0,00028
Bacteria	*Bacteroidetes*	*Bacteroidia*	*Bacteroidales*	*Porphyromonadaceae*	*Barnesiella*	0,01159725 1	0,02524 7799	0,1604
Bacteria	*Proteobacteria*	*Alphaproteobacteria*	*Rhodospirillales*	*Acetobacteraceae*	*Belnapia*	9,35678E-06	0	0,240955
Bacteria	*Proteobacteria*	*Betaproteobacteria*	*Neisseriales*	*Neisseriaceae*	*Bergeriella*	2,9548E-06	1,30433 E-06	0,311555
Bacteria	*Bacteroidetes*	*Flavobacteriia*	*Flavobacteriales*	*Flavobacteriaceae*	*Bergeyella*	0,08204834 9	9,96825 E-05	0,00028
Bacteria	*Firmicutes*	*Bacilli*	*Bacillales*	*Planococcaceae*	*Bhargavaea*	1,21088E-05	0	0,240955
Bacteria	*Proteobacteria*	*Gammaproteobacteria*	*Pasteurellales*	*Pasteurellaceae*	*Bibersteinia*	1,75521E-05	0,00000 2198	0,845405

Bacteria	*Actinobacteria*	*Actinobacteria*	*Bifidobacteriales*	*Bifidobacteriaceae*	*Bifidobacterium*	4,24439E-05	1,06659E-05	0,08074
Bacteria	*Proteobacteria*	*Deltaproteobacteria*	*Desulfovibrionales*	*Desulfovibrionaceae*	*Bilophila*	9,76043E-06	0,000120605	0,003989
Bacteria	*Proteobacteria*	*Alphaproteobacteria*	*Sphingomonadales*	*Sphingomonadaceae*	*Blastomonas*	0	2,11443E-07	0,393769
Bacteria	*Planctomycetes*	*Planctomycetia*	*Planctomycetales*	*Planctomycetaceae*	*Blastopirellula*	0	1,00017E-06	0,215311
Bacteria	*Firmicutes*	*Clostridia*	*Clostridiales*	*Lachnospiraceae*	*Blautia*	0,000817093	0,003415115	0,010475
Bacteria	*Proteobacteria*	*Betaproteobacteria*	*Burkholderiales*	*Alcaligenaceae*	*Bordetella*	0,001104765	7,7467E-07	8,54E-05
Bacteria	*Proteobacteria*	*Alphaproteobacteria*	*Rhizobiales*	*Bradyrhizobiaceae*	*Bosea*	0	0,01152507	0,00048
Bacteria	*Actinobacteria*	*Actinobacteria*	*Actinomycetales*	*Dermabacteraceae*	*Brachybacterium*	7,04411E-05	0,000247076	0,731467
Bacteria	*Proteobacteria*	*Betaproteobacteria*	*Burkholderiales*	*Comamonadaceae*	*Brachymonas*	6,43141E-05	0,000001526	0,071628
Bacteria	*Spirochaetes*	*Spirochaetia*	*Spirochaetales*	*Brachyspiraceae*	*Brachyspira*	0	2,75573E-05	0,215311
Bacteria	*Proteobacteria*	*Alphaproteobacteria*	*Rhizobiales*	*Bradyrhizobiaceae*	*Bradyrhizobium*	0	0,000471333	0,016307
Bacteria	*Actinobacteria*	*Actinobacteria*	*Actinomycetales*	*Brevibacteriaceae*	*Brevibacterium*	0,000100572	0,000136009	0,044667
Bacteria	*Proteobacteria*	*Alphaproteobacteria*	*Caulobacterales*	*Caulobacteraceae*	*Brevundimonas*	0,000636084	9,65301E-05	0,001072
Bacteria	*Firmicutes*	*Bacilli*	*Bacillales*	*Listeriaceae*	*Brochothrix*	3,87837E-05	0	0,088427
Bacteria	*Actinobacteria*	*Actinobacteria*	*Actinomycetales*	*Propionibacteriaceae*	*Brooklawnia*	3,65668E-05	0	0,240955
Bacteria	*Proteobacteria*	*Alphaproteobacteria*	*Rhizobiales*	*Brucellaceae*	*Brucella*	3,57634E-06	0	0,088427
Bacteria	*Firmicutes*	*Erysipelotrichia*	*Erysipelotrichales*	*Erysipelotrichaceae*	*Bulleidia*	0,000092288	0,001314834	0,001671
Bacteria	*Proteobacteria*	*Gammaproteobacteria*	*Enterobacteriales*	*Enterobacteriaceae*	*Buttiauxella*	0,000019587	0	0,088427
Bacteria	*Firmicutes*	*Clostridia*	*Clostridiales*	*Ruminococcaceae*	*Butyricicoccus*	0,000979883	0,001505645	1
Bacteria	*Bacteroidetes*	*Bacteroidia*	*Bacteroidales*	*Porphyromonadaceae*	*Butyricimonas*	2,63269E-05	6,39855E-05	0,081566

Bacteria	*Firmicutes*	*Clostridia*	*Clostridiales*	*Lachnospiraceae*	*Butyrivibrio*	5,9064E-06	0,000180965	0,062837
Bacteria	*Chloroflexi*	*Caldilineae*	*Caldilineales*	*Caldilineaceae*	*Caldilinea*	0	2,1037E-06	0,393769
Bacteria	*Proteobacteria*	*Alphaproteobacteria*	*Rhizobiales*	*Beijerinckiaceae*	*Camelimonas*	0	0,000008134	0,393769
Bacteria	*Proteobacteria*	*Epsilonproteobacteria*	*Campylobacterales*	*Campylobacteraceae*	*Campylobacter*	0,000304001	0,028826866	0,000937
Bacteria	*Proteobacteria*	*Gammaproteobacteria*	*Candidatus Carsonella*	*Candidatus Carsonella*	*Candidatus Carsonella*	0,000055523	0	0,00024
Bacteria	*Bacteroidetes*	*Flavobacteriia*	*Flavobacteriales*	*Flavobacteriaceae*	*Capnocytophaga*	0,000244748	1,84189E-07	0,000478
Bacteria	*Firmicutes*	*Bacilli*	*Lactobacillales*	*Carnobacteriaceae*	*Carnobacterium*	3,90115E-05	2,11443E-07	0,033134
Bacteria	*Firmicutes*	*Bacilli*	*Bacillales*	*Planococcaceae*	*Caryophanon*	2,09827E-05	3,62958E-05	0,581292
Bacteria	*Proteobacteria*	*Betaproteobacteria*	*Burkholderiales*	*Alcaligenaceae*	*Castellaniella*	0,000003244	0	0,240955
Bacteria	*Firmicutes*	*Erysipelotrichia*	*Erysipelotrichales*	*Erysipelotrichaceae*	*Catenibacterium*	8,29944E-05	0,000855222	0,00294
Bacteria	*Firmicutes*	*Clostridia*	*Clostridiales*	*Lachnospiraceae*	*Catonella*	1,6512E-06	1,74363E-05	0,017977
Bacteria	*Proteobacteria*	*Alphaproteobacteria*	*Caulobacterales*	*Caulobacteraceae*	*Caulobacter*	0	6,57266E-07	0,393769
Bacteria	*Actinobacteria*	*Actinobacteria*	*Actinomycetales*	*Cellulomonadaceae*	*Cellulomonas*	6,09001E-05	7,36758E-07	0,105083
Bacteria	*Firmicutes*	*Clostridia*	*Clostridiales*	*Ruminococcaceae*	*Cellulosibacter*	2,65999E-05	6,53008E-05	0,232121
Bacteria	*Firmicutes*	*Clostridia*	*Clostridiales*	*Lachnospiraceae*	*Cellulosilyticum*	0,000390533	8,54221E-05	0,009809
Bacteria	*Actinobacteria*	*Actinobacteria*	*Actinomycetales*	*Promicromonosporaceae*	*Cellulosimicrobium*	0,000008776	0	0,240955
Bacteria	*Proteobacteria*	*Gammaproteobacteria*	*Pseudomonadales*	*Pseudomonadaceae*	*Cellvibrio*	0	1,03554E-05	0,393769
Bacteria	*Fusobacteria*	*Fusobacteriia*	*Fusobacteriales*	*Fusobacteriaceae*	*Cetobacterium*	1,08134E-06	2,88246E-06	0,649136
Bacteria	*Chlamydiae*	*Chlamydiia*	*Chlamydiales*	*Chlamydiaceae*	*Chlamydia*	3,32529E-05	0	0,240955

Bacteria	*Proteobacteria*	*Deltaproteobacteria*	*Myxococcales*	*Polyangiaceae*	*Chondromyces*	1,27972E-05	0	0,240955
Bacteria	*Bacteroidetes*	*Flavobacteriia*	*Flavobacteriales*	*Flavobacteriaceae*	*Chryseobacterium*	0,00072186 1	0,00011 2955	0,018308
Bacteria	*Actinobacteria*	*Actinobacteria*	*Actinomycetales*	*Micrococcaceae*	*Citricoccus*	0	2,11443 E-07	0,393769
Bacteria	*Synergistetes*	*Synergistia*	*Synergistales*	*Synergistaceae*	*Cloacibacillus*	5,02044E-05	5,29517 E-05	0,08074
Bacteria	*Bacteroidetes*	*Flavobacteriia*	*Flavobacteriales*	*Flavobacteriaceae*	*Cloacibacterium*	0,00020399 6	1,02729 E-05	0,667698
Bacteria	*Firmicutes*	*Clostridia*	*Clostridiales*	*Ruminococcaceae*	*Clostridium III*	1,08134E-05	8,50554 E-05	0,006807
Bacteria	*Firmicutes*	*Clostridia*	*Clostridiales*	*Ruminococcaceae*	*Clostridium IV*	0,00508743 4	0,00461 2574	0,457391
Bacteria	*Firmicutes*	*Clostridia*	*Clostridiales*	*Clostridiaceae 1*	*Clostridium sensu stricto*	0,01201745 1	0,00735 7085	0,038989
Bacteria	*Firmicutes*	*Clostridia*	*Clostridiales*	*Peptostreptococcaceae*	*Clostridium XI*	0,01339832 1	0,00717 5893	0,016639
Bacteria	*Fusobacteria*	*Fusobacteriia*	*Fusobacteriales*	*Fusobacteriaceae*	*Clostridium XIX*	0	0,00000 2368	0,11879
Bacteria	*Firmicutes*	*Clostridia*	*Clostridiales*	*Lachnospiraceae*	*Clostridium XlVa*	0,02473216 3	0,01283 1223	0,016639
Bacteria	*Firmicutes*	*Clostridia*	*Clostridiales*	*Lachnospiraceae*	*Clostridium XlVb*	0,00121196 6	0,00072 9861	0,016639
Bacteria	*Firmicutes*	*Erysipelotrichia*	*Erysipelotrichales*	*Erysipelotrichaceae*	*Clostridium XVIII*	9,75154E-06	5,46959 E-06	0,688915
Bacteria	*Actinobacteria*	*Actinobacteria*	*Coriobacteriales*	*Coriobacteriaceae*	*Collinsella*	2,33411E-05	0	0,00024
Bacteria	*Proteobacteria*	*Betaproteobacteria*	*Burkholderiales*	*Comamonadaceae*	*Comamonas*	0,00013579 4	0,00010 1488	0,262864
Bacteria	*Proteobacteria*	*Betaproteobacteria*	*Neisseriales*	*Neisseriaceae*	*Conchiformibius*	3,49165E-05	0	0,088427
Bacteria	*Firmicutes*	*Erysipelotrichia*	*Erysipelotrichales*	*Erysipelotrichaceae*	*Coprobacillus*	0,00000877 6	7,07215 E-06	0,562711
Bacteria	*Firmicutes*	*Clostridia*	*Clostridiales*	*Lachnospiraceae*	*Coprococcus*	0,00192138 5	0,00144 7824	0,508883
Bacteria	*Verrucomicrobia*	*Opitutae*	*Puniceicoccales*	*Puniceicoccaceae*	*Coraliomargarita*	0	2,90086 E-07	0,393769
Bacteria	*Actinobacteria*	*Actinobacteria*	*Actinomycetales*	*Corynebacteriaceae*	*Corynebacterium*	0,00114364 4	0,00279 3825	0,246846

Bacteria	*Proteobacteria*	*Gammaproteobacteria*	*Enterobacteriales*	*Enterobacteriaceae*	*Cronobacter*	1,36967E-05	2,26779 E-05	0,193236
Bacteria	*Bacteroidetes*	*Flavobacteriia*	*Flavobacteriales*	*Flavobacteriaceae*	*Cruoricaptor*	0	2,54086 E-06	0,11879
Bacteria	*Proteobacteria*	*Betaproteobacteria*	*Burkholderiales*	*Burkholderiaceae*	*Cupriavidus*	0	2,71135 E-06	0,393769
Bacteria	*Proteobacteria*	*Betaproteobacteria*	*Burkholderiales*	*Comamonadaceae*	*Curvibacter*	0	3,18627 E-06	0,393769
Bacteria	*Firmicutes*	*Clostridia*	*Clostridiales*	*Defluviitaleaceae*	*Defluviitalea*	0	1,79037 E-05	0,033134
Bacteria	*Deinococcus-Thermus*	*Deinococci*	*Deinococcales*	*Deinococcaceae*	*Deinococcus*	2,2016E-06	0	0,240955
Bacteria	*Proteobacteria*	*Betaproteobacteria*	*Burkholderiales*	*Comamonadaceae*	*Delftia*	0,00025072 2	0,00080 051	0,679708
Bacteria	*Actinobacteria*	*Actinobacteria*	*Actinomycetales*	*Dermabacteraceae*	*Dermabacter*	3,32529E-06	0	0,240955
Bacteria	*Actinobacteria*	*Actinobacteria*	*Actinomycetales*	*Dermacoccaceae*	*Dermacoccus*	6,93649E-05	0	0,088427
Bacteria	*Actinobacteria*	*Actinobacteria*	*Actinomycetales*	*Dermatophilaceae*	*Dermatophilus*	0	6,34329 E-07	0,393769
Bacteria	*Firmicutes*	*Bacilli*	*Lactobacillales*	*Carnobacteriaceae*	*Desemzia*	0	1,84189 E-07	0,393769
Bacteria	*Proteobacteria*	*Deltaproteobacteria*	*Desulfovibrionales*	*Desulfomicrobiaceae*	*Desulfomicrobium*	3,68655E-05	5,84784 E-06	0,845405
Bacteria	*Proteobacteria*	*Deltaproteobacteria*	*Desulfovibrionales*	*Desulfovibrionaceae*	*Desulfovibrio*	0,00028892	0,00230 3318	0,014812
Bacteria	*Firmicutes*	*Clostridia*	*Clostridiales*	*Incertae Sedis XI*	*Dethiosulfatibacter*	0	0,00000 021	0,393769
Bacteria	*Proteobacteria*	*Alphaproteobacteria*	*Rhizobiales*	*Hyphomicrobiaceae*	*Devosia*	5,94013E-05	0,00003 2206	0,202876
Bacteria	*Firmicutes*	*Negativicutes*	*Selenomonadales*	*Veillonellaceae*	*Dialister*	7,37311E-06	0,00112 2931	0,062837
Bacteria	*Proteobacteria*	*Betaproteobacteria*	*Burkholderiales*	*Comamonadaceae*	*Diaphorobacter*	0,00013780 3	0	0,000958
Bacteria	*Actinobacteria*	*Actinobacteria*	*Actinomycetales*	*Dietziaceae*	*Dietzia*	0,00011080 8	0,00025 0128	0,351615
Bacteria	*Firmicutes*	*Bacilli*	*Lactobacillales*	*Carnobacteriaceae*	*Dolosigranulum*	4,32537E-06	0	0,240955
Bacteria	*Firmicutes*	*Bacilli*	*Bacillales*	*Bacillaceae 1*	*Domibacillus*	1,44096E-05	0	0,240955

Bacteria	*Firmicutes*	*Clostridia*	*Clostridiales*	*Lachnospiraceae*	*Dorea*	0,000714028	0,001168751	0,804353
Bacteria	*Bacteroidetes*	*Bacteroidia*	*Bacteroidales*	*Porphyromonadaceae*	*Dysgonomonas*	6,65405E-05	4,94735E-07	0,105083
Bacteria	*Actinobacteria*	*Actinobacteria*	*Coriobacteriales*	*Coriobacteriaceae*	*Eggerthella*	0	2,32587E-06	0,393769
Bacteria	*Bacteroidetes*	*Flavobacteriia*	*Flavobacteriales*	*Flavobacteriaceae*	*Elizabethkingia*	0,000196853	6,34329E-07	0,002267
Bacteria	*Elusimicrobia*	*Elusimicrobia*	*Elusimicrobiales*	*Elusimicrobiaceae*	*Elusimicrobium*	0	0,000208382	0,007557
Bacteria	*Bacteroidetes*	*Flavobacteriia*	*Flavobacteriales*	*Flavobacteriaceae*	*Empedobacter*	9,77933E-05	3,94684E-05	0,118152
Bacteria	*Proteobacteria*	*Gammaproteobacteria*	*Pseudomonadales*	*Moraxellaceae*	*Enhydrobacter*	0,000577111	9,74639E-06	0,000247
Bacteria	*Proteobacteria*	*Gammaproteobacteria*	*Enterobacteriales*	*Enterobacteriaceae*	*Enterobacter*	4,61002E-05	9,71147E-06	0,018426
Bacteria	*Firmicutes*	*Bacilli*	*Lactobacillales*	*Enterococcaceae*	*Enterococcus*	0,000619571	4,11948E-05	0,011377
Bacteria	*Actinobacteria*	*Actinobacteria*	*Coriobacteriales*	*Coriobacteriaceae*	*Enterorhabdus*	5,4724E-06	2,59843E-06	0,728372
Bacteria	*Firmicutes*	*Erysipelotrichia*	*Erysipelotrichales*	*Erysipelotrichaceae*	*Erysipelothrix*	0	0,000116852	0,007557
Bacteria	*Firmicutes*	*Erysipelotrichia*	*Erysipelotrichales*	*Erysipelotrichaceae*	*Erysipelotrichaceae_incertae_sedis*	0,000299978	0,002680763	0,002239
Bacteria	*Proteobacteria*	*Alphaproteobacteria*	*Sphingomonadales*	*Erythrobacteraceae*	*Erythrobacter*	0,000019264	0	0,240955
Bacteria	*Proteobacteria*	*Gammaproteobacteria*	*Enterobacteriales*	*Enterobacteriaceae*	*Escherichia/Shigella*	0,005282428	0,022126692	0,057543
Bacteria	*Firmicutes*	*Clostridia*	*Clostridiales*	*Ruminococcaceae*	*Ethanoligenens*	4,00008E-05	0,000198388	0,309563
Bacteria	*Firmicutes*	*Clostridia*	*Clostridiales*	*Eubacteriaceae*	*Eubacterium*	0,001177985	0,009923741	0,031803
Bacteria	*Firmicutes*	*Bacilli*	*Lactobacillales*	*Aerococcaceae*	*Facklamia*	0,000332198	0,000145346	0,122519
Bacteria	*Firmicutes*	*Clostridia*	*Clostridiales*	*Ruminococcaceae*	*Faecalibacterium*	0,000975977	0,016317624	0,000957

Bacteria	*Firmicutes*	*Clostridia*	*Clostridiales*	*Ruminococcaceae*	*Fastidiosipila*	0	5,48393 E-05	0,11879
Bacteria	*Fibrobacteres*	*Fibrobacteria*	*Fibrobacterales*	*Fibrobacteraceae*	*Fibrobacter*	0,00020988 6	0,00132 7895	0,025524
Bacteria	*Firmicutes*	*Clostridia*	*Clostridiales*	*Peptostreptococcaceae*	*Filifactor*	1,02387E-05	1,08809 E-05	0,745201
Bacteria	*Firmicutes*	*Clostridia*	*Clostridiales*	*Clostridiales_Incertae Sedis XI*	*Finegoldia*	0,00019692 8	0	0,010871
Bacteria	*Bacteroidetes*	*Sphingobacteriia*	*Sphingobacteriales*	*Chitinophagaceae*	*Flavitalea*	0	2,11443 E-07	0,393769
Bacteria	*Bacteroidetes*	*Flavobacteriia*	*Flavobacteriales*	*Flavobacteriaceae*	*Flavobacterium*	0,00022904	5,65785 E-05	0,08074
Bacteria	*Firmicutes*	*Clostridia*	*Clostridiales*	*Ruminococcaceae*	*Flavonifractor*	0,00486277 4	0,00099 6722	0,00028
Bacteria	*Fusobacteria*	*Fusobacteriia*	*Fusobacteriales*	*Fusobacteriaceae*	*Fusobacterium*	0,00161851 1	0,00111 4342	0,038989
Bacteria	*Actinobacteria*	*Actinobacteria*	*Gaiellales*	*Gaiellaceae*	*Gaiella*	1,6512E-06	0	0,240955
Bacteria	*Bacteroidetes*	*Flavobacteriia*	*Flavobacteriales*	*Flavobacteriaceae*	*Galbibacter*	0	0,00000 042	0,393769
Bacteria	*Firmicutes*	*Clostridia*	*Clostridiales*	*Clostridiales_Incertae Sedis XI*	*Gallicola*	0	3,55411 E-06	0,215311
Bacteria	*Actinobacteria*	*Actinobacteria*	*Bifidobacteriales*	*Bifidobacteriaceae*	*Gardnerella*	0,00000129 6	4,65175 E-06	0,876911
Bacteria	*Bacteroidetes*	*Flavobacteriia*	*Flavobacteriales*	*Flavobacteriaceae*	*Gelidibacter*	2,56703E-05	0	0,240955
Bacteria	*Firmicutes*	*Bacilli*	*Bacillales*	*Bacillales_Incertae Sedis XI*	*Gemella*	0,00016909 1	2,79493 E-05	0,08083
Bacteria	*Gemmatimonadet es*	*Gemmatimonadetes*	*Gemmatimonadales*	*Gemmatimonadaceae*	*Gemmatimonas*	0	0,00000 1162	0,393769
Bacteria	*Firmicutes*	*Clostridia*	*Clostridiales*	*Ruminococcaceae*	*Gemmiger*	0,00174954	0,00754 3141	0,006432
Bacteria	*Firmicutes*	*Bacilli*	*Bacillales*	*Bacillaceae 1*	*Geobacillus*	1,42556E-05	0	0,240955
Bacteria	*Proteobacteria*	*Deltaproteobacteria*	*Desulfuromonadales*	*Geobacteraceae*	*Geopsychrobacter*	0	3,87335 E-07	0,393769
Bacteria	*Actinobacteria*	*Actinobacteria*	*Actinomycetales*	*Bogoriellaceae*	*Georgenia*	0	2,07214 E-05	0,393769
Bacteria	*Firmicutes*	*Bacilli*	*Lactobacillales*	*Aerococcaceae*	*Globicatella*	9,99282E-05	0,00013 7036	0,348499

Bacteria	*Actinobacteria*	*Actinobacteria*	*Coriobacteriales*	*Coriobacteriaceae*	*Gordonibacter*	5,78334E-05	4,08071 E-05	0,16117
Bacteria	*Acidobacteria*	*Acidobacteria_Gp3*	*Gp3*	*Gp3*	*Gp3*	7,31336E-06	0	0,240955
Bacteria	*Acidobacteria*	*Acidobacteria_Gp4*	*Gp4*	*Gp4*	*Gp4*	0,00042213 4	0	0,240955
Bacteria	*Acidobacteria*	*Acidobacteria_Gp6*	*Gp6*	*Gp6*	*Gp6*	0,00001100 8	2,19901 E-05	0,876911
Bacteria	*Cyanobacteria/Chloroplast*	*Cyanobacteria*	*Family I*	*Family I*	*GpI*	0,00010164 9	0	0,003397
Bacteria	*Firmicutes*	*Clostridia*	*Clostridiales*	*Gracilibacteraceae*	*Gracilibacter*	3,35743E-05	0	0,240955
Bacteria	*Firmicutes*	*Bacilli*	*Lactobacillales*	*Carnobacteriaceae*	*Granulicatella*	2,73537E-05	0	0,088427
Bacteria	*Actinobacteria*	*Actinobacteria*	*Actinomycetales*	*Microbacteriaceae*	*Gulosibacter*	2,2016E-06	1,55553 E-05	0,876911
Bacteria	*Proteobacteria*	*Gammaproteobacteria*	*Pasteurellales*	*Pasteurellaceae*	*Haemophilus*	0,00164778 2	0,00271 7378	0,025719
Bacteria	*Bacteroidetes*	*Bacteroidia*	*Bacteroidales*	*Prevotellaceae*	*Hallella*	7,79493E-05	0,00250 1353	0,000524
Bacteria	*Verrucomicrobia*	*Verrucomicrobiae*	*Verrucomicrobiales*	*Verrucomicrobiaceae*	*Haloferula*	2,56703E-05	0	0,240955
Bacteria	*Actinobacteria*	*Actinobacteria*	*Actinomycetales*	*Dermabacteraceae*	*Helcobacillus*	0,00001643 2	0	0,088427
Bacteria	*Firmicutes*	*Clostridia*	*Clostridiales*	*Clostridiales_Incertae Sedis XI*	*Helcococcus*	0,0003125	0,00000 7761	0,034509
Bacteria	*Proteobacteria*	*Epsilonproteobacteria*	*Campylobacterales*	*Helicobacteraceae*	*Helicobacter*	0,00014302 7	0,00015 1244	0,865516
Bacteria	*Firmicutes*	*Clostridia*	*Clostridiales*	*Lachnospiraceae*	*Hespellia*	3,68655E-05	2,02257 E-06	0,845405
Bacteria	*Proteobacteria*	*Alphaproteobacteria*	*Rhizobiales*	*Phyllobacteriaceae*	*Hoeflea*	7,31336E-06	0	0,240955
Bacteria	*Firmicutes*	*Erysipelotrichia*	*Erysipelotrichales*	*Erysipelotrichaceae*	*Holdemania*	0,00026865 1	0,00078 9688	0,030314
Bacteria	*Firmicutes*	*Clostridia*	*Clostridiales*	*Lachnospiraceae*	*Howardella*	0	2,19179 E-05	0,063994
Bacteria	*Proteobacteria*	*Epsilonproteobacteria*	*Campylobacterales*	*Hydrogenimonaceae*	*Hydrogenimonas*	0	6,03093 E-07	0,393769
Bacteria	*Firmicutes*	*Clostridia*	*Clostridiales*	*Ruminococcaceae*	*Hydrogenoanaerobacterium*	0,00022621 3	2,96312 E-05	0,009323
Bacteria	*Aquificae*	*Aquificae*	*Aquificales*	*Aquificaceae*	*Hydrogenobacter*	0	7,7467E -07	0,393769

Bacteria	*Proteobacteria*	*Betaproteobacteria*	*Burkholderiales*	*Comamonadaceae*	*Hydrogenophaga*	2,85225E-06	1,84189 E-07	0,756733
Bacteria	*Bacteroidetes*	*Sphingobacteriia*	*Sphingobacteriales*	*Chitinophagaceae*	*Hydrotalea*	0	4,94735 E-07	0,393769
Bacteria	*Bacteroidetes*	*Cytophagia*	*Cytophagales*	*Cytophagaceae*	*Hymenobacter*	0,00000877 6	0	0,240955
Bacteria	*Proteobacteria*	*Alphaproteobacteria*	*Rhizobiales*	*Hyphomicrobiaceae*	*Hyphomicrobium*	4,40319E-06	0,00015 9602	0,263359
Bacteria	*Actinobacteria*	*Actinobacteria*	*Acidimicrobiales*	*Acidimicrobiaceae*	*Ilumatobacter*	3,84947E-05	6,34329 E-07	0,298379
Bacteria	*Proteobacteria*	*Alphaproteobacteria*	*Rhodospirillales*	*Rhodospirillaceae*	*Insolitispirillum*	0,00333733 9	0,00000 084	8,54E-05
Bacteria	*Actinobacteria*	*Actinobacteria*	*Actinomycetales*	*Intrasporangiaceae*	*Janibacter*	2,98895E-05	1,36504 E-05	0,1625
Bacteria	*Proteobacteria*	*Betaproteobacteria*	*Burkholderiales*	*Oxalobacteraceae*	*Janthinobacterium*	0	5,52568 E-07	0,393769
Bacteria	*Firmicutes*	*Bacilli*	*Bacillales*	*Staphylococcaceae*	*Jeotgalicoccus*	0,00040817 3	0,00143 2052	0,404809
Bacteria	*Firmicutes*	*Clostridia*	*Clostridiales*	*Lachnospiraceae*	*Johnsonella*	2,16269E-06	0	0,240955
Bacteria	*Synergistetes*	*Synergistia*	*Synergistales*	*Synergistaceae*	*Jonquetella*	0	1,28304 E-05	0,11879
Bacteria	*Firmicutes*	*Erysipelotrichia*	*Erysipelotrichales*	*Erysipelotrichaceae*	*Kandleria*	0,00003317 9	4,60169 E-05	0,745201
Bacteria	*Actinobacteria*	*Actinobacteria*	*Actinomycetales*	*Kineosporiaceae*	*Kineococcus*	5,54216E-06	0	0,240955
Bacteria	*Proteobacteria*	*Betaproteobacteria*	*Neisseriales*	*Neisseriaceae*	*Kingella*	0,00422679 5	6,31892 E-05	0,000247
Bacteria	*Proteobacteria*	*Gammaproteobacteria*	*Enterobacteriales*	*Enterobacteriaceae*	*Klebsiella*	0,08805895 2	9,13837 E-05	0,000263
Bacteria	*Actinobacteria*	*Actinobacteria*	*Actinomycetales*	*Micrococcaceae*	*Kocuria*	0,00021088 6	2,19089 E-07	8,54E-05
Bacteria	*Proteobacteria*	*Deltaproteobacteria*	*Myxococcales*	*Kofleriaceae*	*Kofleria*	0,00000324 4	0	0,240955
Bacteria	*Firmicutes*	*Bacilli*	*Bacillales*	*Planococcaceae*	*Kurthia*	0,00033030 5	0,00214 5709	0,866788
Bacteria	*Actinobacteria*	*Actinobacteria*	*Actinomycetales*	*Dermacoccaceae*	*Kytococcus*	1,66524E-05	0	0,088427
Bacteria	*Proteobacteria*	*Alphaproteobacteria*	*Rhizobiales*	*Xanthobacteraceae*	*Labrys*	0	9,58885 E-05	0,215311

Bacteria	*Firmicutes*	*Clostridia*	*Clostridiales*	*Lachnospiraceae*	*Lachnoanaerobaculum*	9,19418E-05	3,42424 E-05	0,699444
Bacteria	*Firmicutes*	*Clostridia*	*Clostridiales*	*Lachnospiraceae*	*Lachnobacterium*	0	2,25195 E-05	0,016307
Bacteria	*Firmicutes*	*Clostridia*	*Clostridiales*	*Lachnospiraceae*	*Lachnospira*	3,05014E-06	2,94039 E-05	0,0052
Bacteria	*Firmicutes*	*Clostridia*	*Clostridiales*	*Lachnospiraceae*	*Lachnospiracea_incertae_ sedis*	0,00336800 5	0,01086 6986	0,116677
Bacteria	*Firmicutes*	*Bacilli*	*Lactobacillales*	*Lactobacillaceae*	*Lactobacillus*	0,01401026 1	0,04401 9698	0,031803
Bacteria	*Firmicutes*	*Bacilli*	*Lactobacillales*	*Streptococcaceae*	*Lactococcus*	0,00014549 8	0,00002 1169	0,001502
Bacteria	*Firmicutes*	*Clostridia*	*Clostridiales*	*Lachnospiraceae*	*Lactonifactor*	2,89948E-05	4,60225 E-05	0,763965
Bacteria	*Firmicutes*	*Bacilli*	*Lactobacillales*	*Streptococcaceae*	*Lactovum*	2,56347E-06	1,33581 E-05	0,198154
Bacteria	*Latescibacteria*	*Latescibacteria_genera_i ncertae_sedis*	*Latescibacteria_genera _incertae_sedis*	*Latescibacteria_genera_incer tae_sedis*	*Latescibacteria_genera_i ncertae_sedis*	0	1,74051 E-06	0,393769
Bacteria	*Proteobacteria*	*Gammaproteobacteria*	*Enterobacteriales*	*Enterobacteriaceae*	*Leclercia*	2,16741E-05	7,01232 E-07	0,042978
Bacteria	*Proteobacteria*	*Gammaproteobacteria*	*Legionellales*	*Legionellaceae*	*Legionella*	7,5694E-06	0	0,240955
Bacteria	*Proteobacteria*	*Gammaproteobacteria*	*Enterobacteriales*	*Enterobacteriaceae*	*Lelliottia*	2,85225E-06	0	0,240955
Bacteria	*Fusobacteria*	*Fusobacteriia*	*Fusobacteriales*	*Leptotrichiaceae*	*Leptotrichia*	0,00037118 6	0,00006 8242	0,137807
Bacteria	*Actinobacteria*	*Actinobacteria*	*Actinomycetales*	*Microbacteriaceae*	*Leucobacter*	0,00003985 8	1,89614 E-05	0,293984
Bacteria	*Firmicutes*	*Bacilli*	*Lactobacillales*	*Leuconostocaceae*	*Leuconostoc*	0,00019251 8	3,08609 E-05	0,004916
Bacteria	*Proteobacteria*	*Gammaproteobacteria*	*Xanthomonadales*	*Xanthomonadaceae*	*Luteimonas*	5,25468E-05	2,11443 E-07	0,105083
Bacteria	*Actinobacteria*	*Actinobacteria*	*Actinomycetales*	*Propionibacteriaceae*	*Luteococcus*	0,00001466 5	3,9283E -06	0,643325
Bacteria	*Firmicutes*	*Clostridia*	*Clostridiales*	*Gracilibacteraceae*	*Lutispora*	4,84233E-05	5,83238 E-06	0,367728
Bacteria	*Proteobacteria*	*Gammaproteobacteria*	*Xanthomonadales*	*Xanthomonadaceae*	*Lysobacter*	2,85476E-05	4,94735 E-07	0,756733

Bacteria	*Firmicutes*	*Bacilli*	*Bacillales*	*Staphylococcaceae*	*Macrococcus*	0	2,36616 E-05	0,393769
Bacteria	*Proteobacteria*	*Gammaproteobacteria*	*Pasteurellales*	*Pasteurellaceae*	*Mannheimia*	0,00002201 6	3,38309 E-06	0,756733
Bacteria	*Proteobacteria*	*Gammaproteobacteria*	*Oceanospirillales*	*Oceanospirillaceae*	*Marinospirillum*	3,36415E-05	0	0,240955
Bacteria	*Proteobacteria*	*Alphaproteobacteria*	*Rhodospirillales*	*Rhodospirillaceae*	*Marispirillum*	0	0,00000 021	0,393769
Bacteria	*Actinobacteria*	*Actinobacteria*	*Actinomycetales*	*Nocardioidaceae*	*Marmoricola*	1,08134E-06	0	0,240955
Bacteria	*Proteobacteria*	*Alphaproteobacteria*	*Rhizobiales*	*Aurantimonadaceae*	*Martelella*	0,00000129 6	0	0,240955
Bacteria	*Firmicutes*	*Clostridia*	*Clostridiales*	*Lachnospiraceae*	*Marvinbryantia*	0,00059134 7	1,41482 E-06	0,00016
Bacteria	*Proteobacteria*	*Betaproteobacteria*	*Burkholderiales*	*Oxalobacteraceae*	*Massilia*	4,14254E-05	0	0,010871
Bacteria	*Firmicutes*	*Negativicutes*	*Selenomonadales*	*Veillonellaceae*	*Megasphaera*	0,00032724 5	0,04975 0739	0,00128
Bacteria	*Bacteroidetes*	*Cytophagia*	*Cytophagales*	*Cytophagaceae*	*Meniscus*	0	1,96809 E-06	0,11879
Bacteria	*Proteobacteria*	*Alphaproteobacteria*	*Rhizobiales*	*Phyllobacteriaceae*	*Mesorhizobium*	0,00013249 1	0,00084 7639	0,933608
Archaea	*Euryarchaeota*	*Methanobacteria*	*Methanobacteriales*	*Methanobacteriaceae*	*Methanobrevibacter*	0,00036087 3	0,00080 9144	0,186449
Archaea	*Euryarchaeota*	*Methanobacteria*	*Methanobacteriales*	*Methanobacteriaceae*	*Methanosphaera*	0	7,01232 E-07	0,393769
Bacteria	*Proteobacteria*	*Alphaproteobacteria*	*Rhizobiales*	*Methylobacteriaceae*	*Methylobacterium*	9,37907E-05	0,00001 0458	0,027679
Bacteria	*Actinobacteria*	*Actinobacteria*	*Actinomycetales*	*Microbacteriaceae*	*Microbacterium*	0,00023982 2	0,00005 7493	0,002708
Bacteria	*Actinobacteria*	*Actinobacteria*	*Actinomycetales*	*Micrococcaceae*	*Micrococcus*	0,00059333 8	4,19139 E-05	0,000517
Bacteria	*Actinobacteria*	*Actinobacteria*	*Actinomycetales*	*Nocardiaceae*	*Millisia*	1,84328E-06	6,57266 E-07	0,756733
Bacteria	*Firmicutes*	*Negativicutes*	*Selenomonadales*	*Veillonellaceae*	*Mitsuokella*	0,00000324 4	0,00190 1084	0,000376
Bacteria	*Firmicutes*	*Clostridia*	*Clostridiales*	*Clostridiales_Incertae Sedis XIII*	*Mogibacterium*	7,82044E-05	2,66556 E-05	0,475912
Bacteria	*Bacteroidetes*	*Flavobacteriia*	*Flavobacteriales*	*Flavobacteriaceae*	*Moheibacter*	0	1,48631 E-05	0,215311

Bacteria	*Proteobacteria*	*Gammaproteobacteria*	*Pseudomonadales*	*Moraxellaceae*	*Moraxella*	0,34040911	0,031895711	0,000524
Bacteria	*Firmicutes*	*Clostridia*	*Clostridiales*	*Lachnospiraceae*	*Moryella*	4,06014E-06	0,00000021	0,298379
Bacteria	*Deferribacteres*	*Deferribacteres*	*Deferribacterales*	*Deferribacteraceae*	*Mucispirillum*	0,002507097	0,001570697	0,016591
Bacteria	*Actinobacteria*	*Actinobacteria*	*Actinomycetales*	*Mycobacteriaceae*	*Mycobacterium*	1,40237E-05	0	0,088427
Bacteria	*Tenericutes*	*Mollicutes*	*Mycoplasmatales*	*Mycoplasmataceae*	*Mycoplasma*	0,067179197	0,103236434	0,508883
Bacteria	*Bacteroidetes*	*Flavobacteriia*	*Flavobacteriales*	*Flavobacteriaceae*	*Myroides*	5,18897E-05	2,34003E-05	0,483569
Bacteria	*Firmicutes*	*Clostridia*	*Clostridiales*	*Natranaerovirga*	*Natranaerovirga*	0	0,00000042	0,393769
Bacteria	*Proteobacteria*	*Betaproteobacteria*	*Burkholderiales*	*Oxalobacteraceae*	*Naxibacter*	1,46267E-06	0	0,240955
Bacteria	*Proteobacteria*	*Betaproteobacteria*	*Neisseriales*	*Neisseriaceae*	*Neisseria*	0,010135848	0,00018857	0,000278
Bacteria	*Actinobacteria*	*Actinobacteria*	*Actinomycetales*	*Micrococcaceae*	*Nesterenkonia*	4,43296E-05	1,45694E-05	1
Bacteria	*Proteobacteria*	*Gammaproteobacteria*	*Pasteurellales*	*Pasteurellaceae*	*Nicoletella*	2,85225E-06	0	0,240955
Bacteria	*Proteobacteria*	*Alphaproteobacteria*	*Rhizobiales*	*Bradyrhizobiaceae*	*Nitrobacter*	0	4,22454E-06	0,11879
Bacteria	*Actinobacteria*	*Actinobacteria*	*Actinomycetales*	*Nocardioidaceae*	*Nocardioides*	7,30464E-05	1,95496E-05	0,141383
Bacteria	*Actinobacteria*	*Actinobacteria*	*Actinomycetales*	*Nocardiopsaceae*	*Nocardiopsis*	0	5,43338E-05	0,11879
Bacteria	*Firmicutes*	*Bacilli*	*Bacillales*	*Staphylococcaceae*	*Nosocomiicoccus*	0	2,60879E-06	0,215311
Bacteria	*Proteobacteria*	*Alphaproteobacteria*	*Sphingomonadales*	*Sphingomonadaceae*	*Novosphingobium*	2,92535E-05	9,62066E-05	0,876911
Bacteria	*Proteobacteria*	*Alphaproteobacteria*	*Rhizobiales*	*Brucellaceae*	*Ochrobactrum*	0,000416046	1,01915E-05	0,000673
Bacteria	*Bacteroidetes*	*Bacteroidia*	*Bacteroidales*	*Porphyromonadaceae*	*Odoribacter*	0,002297798	2,68787E-05	0,000195
Bacteria	*Proteobacteria*	*Alphaproteobacteria*	*Rhizobiales*	*Bradyrhizobiaceae*	*Oligotropha*	0	4,34457E-06	0,063994
Bacteria	*Actinobacteria*	*Actinobacteria*	*Coriobacteriales*	*Coriobacteriaceae*	*Olsenella*	8,26371E-06	9,33432E-05	0,062473

Bacteria	*Firmicutes*	*Clostridia*	*Clostridiales*	*Lachnospiraceae*	*Oribacterium*	0,000164672	0,000993875	0,016639
Bacteria	*Actinobacteria*	*Actinobacteria*	*Actinomycetales*	*Intrasporangiaceae*	*Ornithinimicrobium*	2,04432E-05	2,19089E-06	0,132342
Bacteria	*Bacteroidetes*	*Flavobacteriia*	*Flavobacteriales*	*Flavobacteriaceae*	*Ornithobacterium*	0,000123184	0,00019818	0,67201
Bacteria	*Firmicutes*	*Clostridia*	*Clostridiales*	*Ruminococcaceae*	*Oscillibacter*	0,015873942	0,014835897	0,679708
Bacteria	*Proteobacteria*	*Gammaproteobacteria*	*Pasteurellales*	*Pasteurellaceae*	*Otariodibacter*	2,86735E-05	9,70645E-06	0,861403
Bacteria	*Firmicutes*	*Clostridia*	*Clostridiales*	*Other*	*Other*	0,0201456	0,016047997	0,186449
Bacteria	*Firmicutes*	*Clostridia*	*Clostridiales*	*Ruminococcaceae*	*Other*	0,014933446	0,024333589	0,363722
Bacteria	*Bacteroidetes*	*Other*	*Other*	*Other*	*Other*	0,001063238	0,010887235	0,002249
Bacteria	*Firmicutes*	*Clostridia*	*Clostridiales*	*Lachnospiraceae*	*Other*	0,033862209	0,009474602	0,000711
Bacteria	*Other*	*Other*	*Other*	*Other*	*Other*	0,024352306	0,007533596	0,001703
Unclassified	*Other*	*Other*	*Other*	*Other*	*Other*	0,009465862	0,001806894	0,001703
Bacteria	*Firmicutes*	*Bacilli*	*Lactobacillales*	*Enterococcaceae*	*Other*	0,007916621	0,001387831	0,001703
Bacteria	*Bacteroidetes*	*Bacteroidia*	*Bacteroidales*	*Other*	*Other*	0,004959652	0,007341611	0,508883
Bacteria	*Firmicutes*	*Other*	*Other*	*Other*	*Other*	0,002124907	0,002376888	0,457391
Bacteria	*Bacteroidetes*	*Sphingobacteriia*	*Sphingobacteriales*	*Chitinophagaceae*	*Other*	0,002003457	0,000706989	0,013243
Bacteria	*Bacteroidetes*	*Bacteroidia*	*Bacteroidales*	*Porphyromonadaceae*	*Other*	0,00124659	0,006836671	0,013243
Bacteria	*Firmicutes*	*Negativicutes*	*Selenomonadales*	*Veillonellaceae*	*Other*	0,000973482	0,003913863	0,057543
Bacteria	*Firmicutes*	*Clostridia*	*Clostridiales*	*Clostridiaceae 1*	*Other*	0,000831757	0,000304854	0,016639

Bacteria	*Firmicutes*	*Clostridia*	*Clostridiales*	*Peptostreptococcaceae*	*Other*	0,000774828	0,000244876	0,003699
Bacteria	*Actinobacteria*	*Actinobacteria*	*Actinomycetales*	*Other*	*Other*	0,000696558	6,99622E-05	0,000495
Bacteria	*Bacteroidetes*	*Flavobacteriia*	*Flavobacteriales*	*Flavobacteriaceae*	*Other*	0,00062675	0,00871136	0,006432
Bacteria	*Proteobacteria*	*Deltaproteobacteria*	*Desulfovibrionales*	*Desulfovibrionaceae*	*Other*	0,000504303	0,000100472	0,000521
Bacteria	*Fusobacteria*	*Fusobacteriia*	*Fusobacteriales*	*Leptotrichiaceae*	*Other*	0,000467015	0,000023359	0,011356
Bacteria	*Firmicutes*	*Erysipelotrichia*	*Erysipelotrichales*	*Erysipelotrichaceae*	*Other*	0,00037528	0,001302102	0,010475
Bacteria	*Proteobacteria*	*Alphaproteobacteria*	*Other*	*Other*	*Other*	0,000361368	0,000168613	0,738964
Bacteria	*Proteobacteria*	*Other*	*Other*	*Other*	*Other*	0,000347396	0,000434698	0,1372
Bacteria	*Proteobacteria*	*Gammaproteobacteria*	*Enterobacteriales*	*Enterobacteriaceae*	*Other*	0,00033715	0,000400217	0,408961
Bacteria	*Proteobacteria*	*Betaproteobacteria*	*Neisseriales*	*Neisseriaceae*	*Other*	0,00031669	0,000133541	0,015006
Unassignable	*Other*	*Other*	*Other*	*Other*	*Other*	0,000164052	0	0,240955
Bacteria	*Proteobacteria*	*Gammaproteobacteria*	*Pasteurellales*	*Pasteurellaceae*	*Other*	0,000157209	0,000108771	0,32175
Bacteria	*Bacteroidetes*	*Bacteroidia*	*Bacteroidales*	*Prevotellaceae*	*Other*	0,00015075	0,003676419	0,000384
Archaea	*Other*	*Other*	*Other*	*Other*	*Other*	0,000139114	6,07902E-05	0,015006
Bacteria	*Proteobacteria*	*Gammaproteobacteria*	*Other*	*Other*	*Other*	0,000132816	7,05383E-05	0,408961
Bacteria	*Planctomycetes*	*Planctomycetia*	*Planctomycetales*	*Planctomycetaceae*	*Other*	0,000131638	0,001346548	0,001695
Bacteria	*Bacteroidetes*	*Bacteroidia*	*Bacteroidales*	*Marinilabiaceae*	*Other*	0,0001249	0,000259682	0,066842
Bacteria	*Proteobacteria*	*Gammaproteobacteria*	*Pseudomonadales*	*Moraxellaceae*	*Other*	0,000116488	1,86123E-05	0,015697

Bacteria	*Actinobacteria*	*Actinobacteria*	*Coriobacteriales*	*Coriobacteriaceae*	*Other*	0,000115446	0,000288724	0,031728
Bacteria	*Firmicutes*	*Clostridia*	*Clostridiales*	*Clostridiales_Incertae Sedis XI*	*Other*	0,000101047	5,80171E-07	0,033134
Bacteria	*Firmicutes*	*Clostridia*	*Other*	*Other*	*Other*	7,97182E-05	0,000124754	0,740743
Bacteria	*Actinobacteria*	*Actinobacteria*	*Actinomycetales*	*Microbacteriaceae*	*Other*	7,47374E-05	3,65776E-06	0,08083
Bacteria	*Synergistetes*	*Synergistia*	*Synergistales*	*Synergistaceae*	*Other*	6,27505E-05	1,61566E-05	0,35801
Bacteria	*Proteobacteria*	*Alphaproteobacteria*	*Rhizobiales*	*Hyphomicrobiaceae*	*Other*	0,000054022	2,90086E-07	0,298379
Bacteria	*Proteobacteria*	*Betaproteobacteria*	*Burkholderiales*	*Other*	*Other*	0,00005146	0,000179094	0,018093
Bacteria	*Proteobacteria*	*Betaproteobacteria*	*Other*	*Other*	*Other*	0,000040429	0,000252542	0,059099
Bacteria	*Proteobacteria*	*Alphaproteobacteria*	*Rhizobiales*	*Other*	*Other*	0,000038879	5,11929E-06	0,229758
Archaea	*Crenarchaeota*	*Thermoprotei*	*Other*	*Other*	*Other*	3,68849E-05	9,37132E-06	0,018426
Bacteria	*Proteobacteria*	*Deltaproteobacteria*	*Other*	*Other*	*Other*	3,49028E-05	0,000608921	0,000637
Bacteria	*Bacteroidetes*	*Sphingobacteriia*	*Sphingobacteriales*	*Sphingobacteriaceae*	*Other*	3,28427E-05	0,000880639	0,095688
Bacteria	*Proteobacteria*	*Alphaproteobacteria*	*Rhizobiales*	*Phyllobacteriaceae*	*Other*	3,13357E-05	2,74876E-06	0,756733
Bacteria	*Firmicutes*	*Bacilli*	*Other*	*Other*	*Other*	3,06185E-05	5,02519E-05	0,183584
Bacteria	*Lentisphaerae*	*Other*	*Other*	*Other*	*Other*	3,03771E-05	5,52568E-07	0,298379
Bacteria	*Firmicutes*	*Bacilli*	*Lactobacillales*	*Other*	*Other*	2,98316E-05	0,000527908	0,00634
Bacteria	*Proteobacteria*	*Betaproteobacteria*	*Burkholderiales*	*Comamonadaceae*	*Other*	2,44359E-05	3,42038E-06	0,915168
Bacteria	*Actinobacteria*	*Actinobacteria*	*Actinomycetales*	*Nocardioidaceae*	*Other*	2,37895E-05	0	0,240955
Bacteria	*Proteobacteria*	*Alphaproteobacteria*	*Sphingomonadales*	*Sphingomonadaceae*	*Other*	1,86026E-05	3,37978E-06	0,915168

Bacteria	*Firmicutes*	*Bacilli*	*Lactobacillales*	*Aerococcaceae*	*Other*	1,69478E-05	6,21013 E-06	1
Bacteria	*Verrucomicrobia*	*Opitutae*	*Puniceicoccales*	*Puniceicoccaceae*	*Other*	1,29761E-05	0,00000 063	0,756733
Bacteria	*Proteobacteria*	*Alphaproteobacteria*	*Rhodospirillales*	*Rhodospirillaceae*	*Other*	1,01664E-05	0,00007 8362	0,90784
Bacteria	*Proteobacteria*	*Alphaproteobacteria*	*Rhodospirillales*	*Other*	*Other*	8,85959E-06	0	0,240955
Bacteria	*Proteobacteria*	*Deltaproteobacteria*	*Desulfovibrionales*	*Other*	*Other*	0,00000877 6	6,02593 E-06	0,229758
Bacteria	*Proteobacteria*	*Gammaproteobacteria*	*Xanthomonadales*	*Xanthomonadaceae*	*Other*	7,5694E-06	1,51061 E-05	0,876911
Bacteria	*Chlamydiae*	*Chlamydiia*	*Chlamydiales*	*Parachlamydiaceae*	*Other*	7,5694E-06	0	0,240955
Bacteria	*Firmicutes*	*Bacilli*	*Lactobacillales*	*Carnobacteriaceae*	*Other*	7,54908E-06	0,00030 6338	0,00462
Bacteria	*Proteobacteria*	*Gammaproteobacteria*	*Pseudomonadales*	*Other*	*Other*	7,09263E-06	7,01232 E-07	0,132342
Bacteria	*Firmicutes*	*Bacilli*	*Lactobacillales*	*Lactobacillaceae*	*Other*	6,44519E-06	0,00017 6725	0,002907
Archaea	*Euryarchaeota*	*Thermoplasmata*	*Thermoplasmatales*	*Other*	*Other*	5,9064E-06	1,48054 E-06	0,562711
Bacteria	*Proteobacteria*	*Gammaproteobacteria*	*Chromatiales*	*Other*	*Other*	5,40671E-06	0	0,240955
Bacteria	*Actinobacteria*	*Actinobacteria*	*Actinomycetales*	*Pseudonocardiaceae*	*Other*	3,68655E-06	0	0,240955
Bacteria	*Proteobacteria*	*Betaproteobacteria*	*Burkholderiales*	*Sutterellaceae*	*Other*	2,85225E-06	0,00000 3669	0,562711
Bacteria	*Actinobacteria*	*Actinobacteria*	*Actinomycetales*	*Propionibacteriaceae*	*Other*	2,59194E-06	0	0,240955
Bacteria	*Proteobacteria*	*Gammaproteobacteria*	*Pseudomonadales*	*Pseudomonadaceae*	*Other*	2,39677E-06	3,25361 E-05	0,435428
Bacteria	*Bacteroidetes*	*Bacteroidia*	*Bacteroidales*	*Rikenellaceae*	*Other*	2,39368E-06	0	0,088427
Bacteria	*Proteobacteria*	*Betaproteobacteria*	*Rhodocyclales*	*Rhodocyclaceae*	*Other*	2,21686E-06	3,38309 E-06	0,876911
Bacteria	*Firmicutes*	*Negativicutes*	*Selenomonadales*	*Other*	*Other*	1,84328E-06	0,00011 4573	0,00844
Bacteria	*Proteobacteria*	*Alphaproteobacteria*	*Rhodobacterales*	*Rhodobacteraceae*	*Other*	1,84328E-06	1,93281 E-05	0,649136
Bacteria	*Chloroflexi*	*Other*	*Other*	*Other*	*Other*	1,84328E-06	0	0,240955
Bacteria	*Proteobacteria*	*Alphaproteobacteria*	*Caulobacterales*	*Caulobacteraceae*	*Other*	1,65883E-06	2,11443 E-07	0,298379

Bacteria	*Tenericutes*	*Mollicutes*	*Other*	*Other*	*Other*	1,1008E-06	0	0,240955
Bacteria	*Actinobacteria*	*Actinobacteria*	*Actinomycetales*	*Micrococcaceae*	*Other*	5,50399E-07	2,83857E-06	0,220572
Bacteria	*Firmicutes*	*Bacilli*	*Bacillales*	*Other*	*Other*	5,50399E-07	2,07377E-06	0,263359
Bacteria	*Fusobacteria*	*Fusobacteriia*	*Fusobacteriales*	*Fusobacteriaceae*	*Other*	5,50399E-07	6,1439E-07	0,845405
Bacteria	*Proteobacteria*	*Betaproteobacteria*	*Burkholderiales*	*Alcaligenaceae*	*Other*	5,50399E-07	0	0,240955
Bacteria	*Bacteroidetes*	*Sphingobacteriia*	*Sphingobacteriales*	*Other*	*Other*	0	0,000384509	0,003268
Bacteria	*Spirochaetes*	*Spirochaetia*	*Spirochaetales*	*Spirochaetaceae*	*Other*	0	0,000164305	0,003268
Bacteria	*Firmicutes*	*Negativicutes*	*Selenomonadales*	*Acidaminococcaceae*	*Other*	0	0,00013859	0,001308
Bacteria	*Firmicutes*	*Clostridia*	*Clostridiales*	*Clostridiales_Incertae Sedis XIII*	*Other*	0	0,000110013	0,00016
Bacteria	*Actinobacteria*	*Actinobacteria*	*Other*	*Other*	*Other*	0	6,64059E-05	0,11879
Bacteria	*Proteobacteria*	*Epsilonproteobacteria*	*Campylobacterales*	*Other*	*Other*	0	4,45188E-05	0,003268
Bacteria	*Spirochaetes*	*Spirochaetia*	*Spirochaetales*	*Other*	*Other*	0	3,62951E-05	0,007557
Bacteria	*Proteobacteria*	*Alphaproteobacteria*	*Rhizobiales*	*Bradyrhizobiaceae*	*Other*	0	2,86715E-05	0,063994
Bacteria	*Firmicutes*	*Bacilli*	*Lactobacillales*	*Streptococcaceae*	*Other*	0	8,81594E-06	0,033134
Bacteria	*Bacteroidetes*	*Flavobacteriia*	*Flavobacteriales*	*Other*	*Other*	0	8,66362E-06	0,007557
Bacteria	*Proteobacteria*	*Epsilonproteobacteria*	*Other*	*Other*	*Other*	0	8,47936E-06	0,033134
Bacteria	*Bacteroidetes*	*Cytophagia*	*Cytophagales*	*Other*	*Other*	0	6,4481E-06	0,033134
Bacteria	*Firmicutes*	*Bacilli*	*Bacillales*	*Planococcaceae*	*Other*	0	4,84568E-06	0,11879
Bacteria	*Cyanobacteria/Chloroplast*	*Chloroplast*	*Chloroplast*	*Chloroplast*	*Other*	0	3,50542E-06	0,393769

Bacteria	*Proteobacteria*	*Gammaproteobacteria*	*Aeromonadales*	*Succinivibrionaceae*	*Other*	0	2,98888 E-06	0,063994
Bacteria	*Proteobacteria*	*Epsilonproteobacteria*	*Campylobacterales*	*Helicobacteraceae*	*Other*	0	2,80493 E-06	0,393769
Archaea	*Euryarchaeota*	*Other*	*Other*	*Other*	*Other*	0	2,78865 E-06	0,215311
Bacteria	*Proteobacteria*	*Betaproteobacteria*	*Burkholderiales*	*Burkholderiales_incertae_sedis*	*Other*	0	0,000002043	0,063994
Bacteria	*Proteobacteria*	*Gammaproteobacteria*	*Aeromonadales*	*Aeromonadaceae*	*Other*	0	1,16034 E-06	0,393769
Bacteria	*Firmicutes*	*Bacilli*	*Bacillales*	*Staphylococcaceae*	*Other*	0	8,41455 E-07	0,215311
Bacteria	*Verrucomicrobia*	*Other*	*Other*	*Other*	*Other*	0	7,10085 E-07	0,215311
Bacteria	*Tenericutes*	*Mollicutes*	*Anaeroplasmatales*	*Anaeroplasmataceae*	*Other*	0	7,01232 E-07	0,393769
Bacteria	*Actinobacteria*	*Actinobacteria*	*Actinomycetales*	*Corynebacteriaceae*	*Other*	0	6,34329 E-07	0,393769
Bacteria	*Bacteroidetes*	*Cytophagia*	*Cytophagales*	*Cytophagaceae*	*Other*	0	0,00000063	0,393769
Archaea	*Euryarchaeota*	*Methanobacteria*	*Methanobacteriales*	*Methanobacteriaceae*	*Other*	0	6,03093 E-07	0,393769
Bacteria	*Firmicutes*	*Clostridia*	*Clostridiales*	*Gracilibacteraceae*	*Other*	0	5,00085 E-07	0,215311
Bacteria	*Proteobacteria*	*Gammaproteobacteria*	*Chromatiales*	*Chromatiaceae*	*Other*	0	4,94735 E-07	0,393769
Bacteria	*Firmicutes*	*Clostridia*	*Clostridiales*	*Incertae Sedis XI*	*Other*	0	4,22886 E-07	0,393769
Bacteria	*Proteobacteria*	*Deltaproteobacteria*	*Desulfovibrionales*	*Desulfohalobiaceae*	*Other*	0	0,00000042	0,393769
Bacteria	*Actinobacteria*	*Actinobacteria*	*Actinomycetales*	*Intrasporangiaceae*	*Other*	0	2,19089 E-07	0,393769
Bacteria	*Proteobacteria*	*Gammaproteobacteria*	*Vibrionales*	*Vibrionaceae*	*Other*	0	2,19089 E-07	0,393769
Bacteria	*Proteobacteria*	*Alphaproteobacteria*	*Caulobacterales*	*Other*	*Other*	0	2,11443 E-07	0,393769

Bacteria	*Proteobacteria*	*Alphaproteobacteria*	*Sphingomonadales*	*Other*	*Other*	0	2,11443 E-07	0,393769
Bacteria	*Firmicutes*	*Clostridia*	*Clostridiales*	*Eubacteriaceae*	*Other*	0	0,00000 021	0,393769
Bacteria	*Proteobacteria*	*Betaproteobacteria*	*Burkholderiales*	*Oxalobacteraceae*	*Oxalobacter*	8,14606E-05	0,00021 6108	0,038646
Bacteria	*Firmicutes*	*Bacilli*	*Bacillales*	*Paenibacillaceae 1*	*Paenibacillus*	1,46267E-06	0	0,240955
Bacteria	*Proteobacteria*	*Alphaproteobacteria*	*Rhizobiales*	*Brucellaceae*	*Paenochrobactrum*	3,13589E-05	0	0,240955
Bacteria	*Bacteroidetes*	*Bacteroidia*	*Bacteroidales*	*Porphyromonadaceae*	*Paludibacter*	0,00105462 5	0,00174 8689	0,283074
Bacteria	*Firmicutes*	*Clostridia*	*Clostridiales*	*Ruminococcaceae*	*Papillibacter*	0,00008537	0,00021 3937	0,508138
Bacteria	*Bacteroidetes*	*Bacteroidia*	*Bacteroidales*	*Porphyromonadaceae*	*Parabacteroides*	0,00215610 3	0,00055 1567	0,000524
Bacteria	*Proteobacteria*	*Alphaproteobacteria*	*Rhodobacterales*	*Rhodobacteraceae*	*Paracoccus*	0,00039197 1	7,38055 E-06	0,088869
Bacteria	*Actinobacteria*	*Actinobacteria*	*Coriobacteriales*	*Coriobacteriaceae*	*Paraeggerthella*	1,84328E-06	1,99887 E-05	0,649136
Bacteria	*Firmicutes*	*Bacilli*	*Lactobacillales*	*Lactobacillaceae*	*Paralactobacillus*	0	0,00014 2141	0,003268
Bacteria	*Bacteroidetes*	*Bacteroidia*	*Bacteroidales*	*Prevotellaceae*	*Paraprevotella*	0,00039670 5	0,01124 9129	0,00028
Bacteria	*Bacteroidetes*	*Sphingobacteriia*	*Sphingobacteriales*	*Chitinophagaceae*	*Parasegetibacter*	0	2,11443 E-07	0,393769
Bacteria	*Firmicutes*	*Clostridia*	*Clostridiales*	*Lachnospiraceae*	*Parasporobacterium*	6,23129E-06	1,90108 E-05	0,245755
Bacteria	*Proteobacteria*	*Betaproteobacteria*	*Burkholderiales*	*Sutterellaceae*	*Parasutterella*	0,00085338 3	8,42983 E-05	0,000273
Bacteria	*Firmicutes*	*Clostridia*	*Clostridiales*	*Clostridiales_Incertae Sedis XI*	*Parvimonas*	0,00012072 8	9,51084 E-06	0,071628
Bacteria	*Proteobacteria*	*Gammaproteobacteria*	*Pasteurellales*	*Pasteurellaceae*	*Pasteurella*	0,00104124 1	4,89688 E-05	0,000273
Bacteria	*Proteobacteria*	*Gammaproteobacteria*	*Enterobacteriales*	*Enterobacteriaceae*	*Pectobacterium*	0	2,19089 E-05	0,393769
Bacteria	*Firmicutes*	*Bacilli*	*Lactobacillales*	*Lactobacillaceae*	*Pediococcus*	0,00011196 2	6,3333E -06	0,000246

Bacteria	*Bacteroidetes*	*Sphingobacteriia*	*Sphingobacteriales*	*Sphingobacteriaceae*	*Pedobacter*	3,79775E-05	1,28933 E-06	0,756733
Bacteria	*Proteobacteria*	*Betaproteobacteria*	*Burkholderiales*	*Comamonadaceae*	*Pelomonas*	0,00016083 4	2,98065 E-05	0,002829
Bacteria	*Firmicutes*	*Clostridia*	*Clostridiales*	*Syntrophomonadaceae*	*Pelospora*	0	0,00000 021	0,393769
Bacteria	*Firmicutes*	*Clostridia*	*Clostridiales*	*Peptococcaceae 1*	*Peptococcus*	0,00011184 7	0,00013 2848	0,677021
Bacteria	*Firmicutes*	*Clostridia*	*Clostridiales*	*Clostridiales_Incertae Sedis XI*	*Peptoniphilus*	0,00039020 9	2,29946 E-05	0,002997
Bacteria	*Firmicutes*	*Clostridia*	*Clostridiales*	*Peptostreptococcaceae*	*Peptostreptococcaceae_i ncertae_sedis*	4,63364E-05	0	0,088427
Bacteria	*Firmicutes*	*Clostridia*	*Clostridiales*	*Peptostreptococcaceae*	*Peptostreptococcus*	0,00020868 9	3,00219 E-05	0,004916
Bacteria	*Proteobacteria*	*Gammaproteobacteria*	*Pseudomonadales*	*Moraxellaceae*	*Perlucidibaca*	1,37816E-05	0	0,240955
Bacteria	*Bacteroidetes*	*Bacteroidia*	*Bacteroidales*	*Porphyromonadaceae*	*Petrimonas*	0	8,13877 E-05	0,007557
Bacteria	*Firmicutes*	*Negativicutes*	*Selenomonadales*	*Acidaminococcaceae*	*Phascolarctobacterium*	0,00104463 5	0,04075 3749	0,00028
Bacteria	*Proteobacteria*	*Alphaproteobacteria*	*Caulobacterales*	*Caulobacteraceae*	*Phenylobacterium*	1,84328E-06	0	0,240955
Bacteria	*Bacteroidetes*	*Bacteroidia*	*Bacteroidales*	*Bacteroidales"_incertae_sedi s*	*Phocaeicola*	0	9,89471 E-07	0,393769
Bacteria	*Proteobacteria*	*Gammaproteobacteria*	*Vibrionales*	*Vibrionaceae*	*Photobacterium*	1,38439E-05	0	0,088427
Bacteria	*Proteobacteria*	*Alphaproteobacteria*	*Rhizobiales*	*Phyllobacteriaceae*	*Phyllobacterium*	0,00020873 2	0,00104 9156	0,408138
Bacteria	*Actinobacteria*	*Actinobacteria*	*Actinomycetales*	*Nocardioidaceae*	*Pimelobacter*	5,50399E-07	0	0,240955
Bacteria	*Planctomycetes*	*Planctomycetia*	*Planctomycetales*	*Planctomycetaceae*	*Pirellula*	0	1,84189 E-07	0,393769
Bacteria	*Bacteroidetes*	*Flavobacteriia*	*Flavobacteriales*	*Flavobacteriaceae*	*Planobacterium*	0,00031138 4	5,15328 E-06	0,000813
Bacteria	*Firmicutes*	*Bacilli*	*Bacillales*	*Planococcaceae*	*Planococcaceae_incertae _sedis*	0,00010821	1,99914 E-05	0,220572
Bacteria	*Firmicutes*	*Bacilli*	*Bacillales*	*Planococcaceae*	*Planomicrobium*	6,89079E-06	0	0,240955
Bacteria	*Bacteroidetes*	*Cytophagia*	*Cytophagales*	*Cytophagaceae*	*Pontibacter*	1,84328E-06	3,98284 E-06	0,876911
Bacteria	*Proteobacteria*	*Alphaproteobacteria*	*Sphingomonadales*	*Erythrobacteraceae*	*Porphyrobacter*	1,84328E-06	0	0,240955

Bacteria	*Bacteroidetes*	*Bacteroidia*	*Bacteroidales*	*Porphyromonadaceae*	*Porphyromonas*	0,000821511	0,000187014	0,026246
Bacteria	*Bacteroidetes*	*Bacteroidia*	*Bacteroidales*	*Prevotellaceae*	*Prevotella*	0,013183826	0,168145744	0,001703
Bacteria	*Bacteroidetes*	*Bacteroidetes"_incertae_sedis*	*Prolixibacter*	*Prolixibacter*	*Prolixibacter*	0	4,41183E-06	0,11879
Bacteria	*Actinobacteria*	*Actinobacteria*	*Actinomycetales*	*Propionibacteriaceae*	*Propionibacterium*	0,002850816	0,000242669	0,000278
Bacteria	*Fusobacteria*	*Fusobacteriia*	*Fusobacteriales*	*Fusobacteriaceae*	*Propionigenium*	0	1,16034E-06	0,393769
Bacteria	*Proteobacteria*	*Betaproteobacteria*	*Rhodocyclales*	*Rhodocyclaceae*	*Propionivibrio*	4,32537E-06	0	0,240955
Bacteria	*Firmicutes*	*Clostridia*	*Clostridiales*	*Clostridiaceae 1*	*Proteiniclasticum*	3,20675E-05	0	0,088427
Bacteria	*Firmicutes*	*Clostridia*	*Clostridiales*	*Peptostreptococcaceae*	*Proteocatella*	0,000050899	4,60086E-06	0,132342
Bacteria	*Proteobacteria*	*Gammaproteobacteria*	*Enterobacteriales*	*Enterobacteriaceae*	*Proteus*	1,18948E-05	0	0,240955
Bacteria	*Proteobacteria*	*Gammaproteobacteria*	*Enterobacteriales*	*Enterobacteriaceae*	*Providencia*	6,47984E-06	0	0,240955
Bacteria	*Firmicutes*	*Clostridia*	*Clostridiales*	*Lachnospiraceae*	*Pseudobutyrivibrio*	5,9064E-06	0,000215932	0,001292
Bacteria	*Firmicutes*	*Clostridia*	*Clostridiales*	*Ruminococcaceae*	*Pseudoflavonifractor*	0,004547896	0,001832117	0,002953
Bacteria	*Proteobacteria*	*Gammaproteobacteria*	*Pseudomonadales*	*Pseudomonadaceae*	*Pseudomonas*	0,002214672	5,44188E-05	0,000195
Bacteria	*Actinobacteria*	*Actinobacteria*	*Actinomycetales*	*Pseudonocardiaceae*	*Pseudonocardia*	3,40265E-06	0	0,088427
Bacteria	*Firmicutes*	*Clostridia*	*Clostridiales*	*Eubacteriaceae*	*Pseudoramibacter*	0	2,90086E-07	0,393769
Bacteria	*Bacteroidetes*	*Sphingobacteriia*	*Sphingobacteriales*	*Sphingobacteriaceae*	*Pseudosphingobacterium*	0	2,85928E-06	0,215311
Bacteria	*Proteobacteria*	*Gammaproteobacteria*	*Xanthomonadales*	*Xanthomonadaceae*	*Pseudoxanthomonas*	4,02014E-05	6,87385E-05	0,782741
Bacteria	*Fusobacteria*	*Fusobacteriia*	*Fusobacteriales*	*Fusobacteriaceae*	*Psychrilyobacter*	1,90147E-05	0	0,240955
Bacteria	*Proteobacteria*	*Gammaproteobacteria*	*Pseudomonadales*	*Moraxellaceae*	*Psychrobacter*	0,000146814	8,87313E-05	0,211448
Bacteria	*Synergistetes*	*Synergistia*	*Synergistales*	*Synergistaceae*	*Pyramidobacter*	2,32771E-05	0,000104708	0,014516
Bacteria	*Proteobacteria*	*Betaproteobacteria*	*Burkholderiales*	*Burkholderiaceae*	*Ralstonia*	8,86745E-06	4,14974E-05	0,311555

Bacteria	*Proteobacteria*	*Gammaproteobacteria*	*Enterobacteriales*	*Enterobacteriaceae*	*Raoultella*	0,000034227	0	0,240955
Bacteria	*Actinobacteria*	*Actinobacteria*	*Actinomycetales*	*Micrococcaceae*	*Renibacterium*	6,52285E-05	7,7467E-07	0,002267
Bacteria	*Proteobacteria*	*Gammaproteobacteria*	*Chromatiales*	*Chromatiaceae*	*Rheinheimera*	0	8,33084E-06	0,11879
Bacteria	*Proteobacteria*	*Alphaproteobacteria*	*Rhizobiales*	*Rhizobiaceae*	*Rhizobium*	0,000532441	0,000168251	0,006409
Bacteria	*Proteobacteria*	*Alphaproteobacteria*	*Rhodobacterales*	*Rhodobacteraceae*	*Rhodobacter*	0	4,27294E-05	0,215311
Bacteria	*Actinobacteria*	*Actinobacteria*	*Actinomycetales*	*Nocardiaceae*	*Rhodococcus*	0,000206968	9,87067E-05	0,127456
Bacteria	*Proteobacteria*	*Alphaproteobacteria*	*Rhodospirillales*	*Acetobacteraceae*	*Rhodovarius*	2,92535E-06	0	0,240955
Bacteria	*Bacteroidetes*	*Bacteroidia*	*Bacteroidales*	*Rikenellaceae*	*Rikenella*	2,56703E-05	0	0,240955
Bacteria	*Firmicutes*	*Clostridia*	*Clostridiales*	*Lachnospiraceae*	*Robinsoniella*	9,50807E-05	2,29884E-05	0,103085
Bacteria	*Firmicutes*	*Clostridia*	*Clostridiales*	*Lachnospiraceae*	*Roseburia*	0,00138735	0,019456389	0,000711
Bacteria	*Proteobacteria*	*Alphaproteobacteria*	*Rhodospirillales*	*Acetobacteraceae*	*Roseomonas*	6,65059E-06	0	0,240955
Bacteria	*Actinobacteria*	*Actinobacteria*	*Actinomycetales*	*Micrococcaceae*	*Rothia*	0,004924722	0,006420408	0,159665
Bacteria	*Proteobacteria*	*Alphaproteobacteria*	*Rhodobacterales*	*Rhodobacteraceae*	*Rubellimicrobium*	2,05899E-05	0	0,031967
Bacteria	*Actinobacteria*	*Actinobacteria*	*Rubrobacterales*	*Rubrobacteraceae*	*Rubrobacter*	0,000011261	0	0,088427
Bacteria	*Proteobacteria*	*Gammaproteobacteria*	*Aeromonadales*	*Succinivibrionaceae*	*Ruminobacter*	3,08223E-05	2,74296E-05	0,044544
Bacteria	*Firmicutes*	*Clostridia*	*Clostridiales*	*Ruminococcaceae*	*Ruminococcus*	0,003641119	0,007809513	0,057543
Bacteria	*Firmicutes*	*Clostridia*	*Clostridiales*	*Lachnospiraceae*	*Ruminococcus2*	0,001274479	0,001398366	0,56326
Bacteria	*Firmicutes*	*Bacilli*	*Bacillales*	*Planococcaceae*	*Rummeliibacillus*	0	2,19089E-07	0,393769
Bacteria	*Candidatus Saccharibacteria*	*Saccharibacteria_genera_incertae_sedis*	*Saccharibacteria_genera_incertae_sedis*	*Saccharibacteria_genera_incertae_sedis*	*Saccharibacteria_genera_incertae_sedis*	0,000687093	0,000219227	0,016449
Bacteria	*Firmicutes*	*Clostridia*	*Clostridiales*	*Ruminococcaceae*	*Saccharofermentans*	2,51707E-05	0,000044163	0,059099
Bacteria	*Bacteroidetes*	*Flavobacteriia*	*Flavobacteriales*	*Flavobacteriaceae*	*Salinimicrobium*	2,16269E-06	0	0,240955

Bacteria	*Firmicutes*	*Clostridia*	*Clostridiales*	*Clostridiaceae 1*	*Sarcina*	1,9688E-06	5,06177E-05	0,014074
Bacteria	*Proteobacteria*	*Betaproteobacteria*	*Burkholderiales*	*Comamonadaceae*	*Schlegelella*	1,39863E-05	0	0,031967
Bacteria	*Firmicutes*	*Negativicutes*	*Selenomonadales*	*Veillonellaceae*	*Schwartzia*	4,95359E-06	0,003425093	0,002997
Bacteria	*Firmicutes*	*Clostridia*	*Halanaerobiales*	*Halobacteroidaceae*	*Selenihalanaerobacter*	0	1,16034E-06	0,393769
Bacteria	*Firmicutes*	*Negativicutes*	*Selenomonadales*	*Veillonellaceae*	*Selenomonas*	1,07998E-05	0,003669862	0,002204
Bacteria	*Firmicutes*	*Erysipelotrichia*	*Erysipelotrichales*	*Erysipelotrichaceae*	*Sharpea*	1,17014E-05	0	0,240955
Bacteria	*Proteobacteria*	*Gammaproteobacteria*	*Alteromonadales*	*Shewanellaceae*	*Shewanella*	2,88192E-05	0	0,240955
Bacteria	*Firmicutes*	*Clostridia*	*Clostridiales*	*Lachnospiraceae*	*Shuttleworthia*	0	2,71755E-06	0,063994
Bacteria	*Proteobacteria*	*Betaproteobacteria*	*Neisseriales*	*Neisseriaceae*	*Simonsiella*	0	3,80341E-06	0,215311
Bacteria	*Proteobacteria*	*Betaproteobacteria*	*Burkholderiales*	*Comamonadaceae*	*Simplicispira*	0	1,41002E-05	0,215311
Bacteria	*Actinobacteria*	*Actinobacteria*	*Actinomycetales*	*Micrococcaceae*	*Sinomonas*	0	2,57865E-06	0,393769
Bacteria	*Actinobacteria*	*Actinobacteria*	*Coriobacteriales*	*Coriobacteriaceae*	*Slackia*	0	2,90086E-07	0,393769
Bacteria	*Firmicutes*	*Bacilli*	*Bacillales*	*Planococcaceae*	*Solibacillus*	0	3,68379E-07	0,393769
Bacteria	*Actinobacteria*	*Actinobacteria*	*Solirubrobacterales*	*Solirubrobacteraceae*	*Solirubrobacter*	2,31679E-05	0	0,088427
Bacteria	*Bacteroidetes*	*Flavobacteriia*	*Flavobacteriales*	*Flavobacteriaceae*	*Soonwooa*	1,29029E-05	2,11443E-07	0,756733
Bacteria	*Chloroflexi*	*Thermomicrobia*	*Sphaerobacterales*	*Sphaerobacteraceae*	*Sphaerobacter*	0	4,26069E-06	0,393769
Bacteria	*Spirochaetes*	*Spirochaetia*	*Spirochaetales*	*Spirochaetaceae*	*Sphaerochaeta*	0,000111198	0,004655045	0,000524
Bacteria	*Bacteroidetes*	*Sphingobacteriia*	*Sphingobacteriales*	*Sphingobacteriaceae*	*Sphingobacterium*	0,000175453	0,000176939	0,264715
Bacteria	*Proteobacteria*	*Alphaproteobacteria*	*Sphingomonadales*	*Sphingomonadaceae*	*Sphingobium*	0,000026102	7,7467E-07	0,033134
Bacteria	*Proteobacteria*	*Alphaproteobacteria*	*Sphingomonadales*	*Sphingomonadaceae*	*Sphingomonas*	0,0005245	0,002956599	0,457391
Bacteria	*Proteobacteria*	*Alphaproteobacteria*	*Sphingomonadales*	*Sphingomonadaceae*	*Sphingopyxis*	2,92535E-06	0	0,240955

Bacteria	*Proteobacteria*	*Alphaproteobacteria*	*Sphingomonadales*	*Sphingomonadaceae*	*Sphingosinicella*	1,55516E-05	0	0,240955
Bacteria	*Firmicutes*	*Clostridia*	*Clostridiales*	*Peptostreptococcaceae*	*Sporacetigenium*	5,15997E-05	7,07145 E-05	0,502247
Bacteria	*Firmicutes*	*Clostridia*	*Clostridiales*	*Ruminococcaceae*	*Sporobacter*	0,00276254 9	0,00169 2381	0,031803
Bacteria	*SR1*	*SR1_genera_incertae_se dis*	*SR1_genera_incertae_s edis*	*SR1_genera_incertae_sedis*	*SR1_genera_incertae_sed is*	3,41247E-05	2,44379 E-05	0,845405
Bacteria	*Firmicutes*	*Bacilli*	*Bacillales*	*Staphylococcaceae*	*Staphylococcus*	0,00486108	0,00262 4935	0,016165
Bacteria	*Proteobacteria*	*Gammaproteobacteria*	*Xanthomonadales*	*Xanthomonadaceae*	*Stenotrophomonas*	0,00051675 5	0,00013 8182	0,007278
Bacteria	*Proteobacteria*	*Gammaproteobacteria*	*Xanthomonadales*	*Sinobacteraceae*	*Steroidobacter*	0	3,18627 E-06	0,393769
Bacteria	*Fusobacteria*	*Fusobacteriia*	*Fusobacteriales*	*Leptotrichiaceae*	*Streptobacillus*	2,91963E-05	1,26866 E-06	0,756733
Bacteria	*Firmicutes*	*Bacilli*	*Lactobacillales*	*Streptococcaceae*	*Streptococcus*	0,01004404 7	0,00981 8849	0,457391
Bacteria	*Actinobacteria*	*Actinobacteria*	*Actinomycetales*	*Streptomycetaceae*	*Streptomyces*	3,25087E-05	7,64099 E-05	0,749296
Bacteria	*Cyanobacteria/Chl oroplast*	*Chloroplast*	*Chloroplast*	*Chloroplast*	*Streptophyta*	0,00140577 4	0,00083 2014	0,002953
Bacteria	*Verrucomicrobia*	*Subdivision5*	*Subdivision5_genera_in certae_sedis*	*Subdivision5_genera_incerta e_sedis*	*Subdivision5_genera_ince rtae_sedis*	0,00038557 9	0,00066 3453	0,741182
Bacteria	*Firmicutes*	*Clostridia*	*Clostridiales*	*Ruminococcaceae*	*Subdoligranulum*	2,85225E-06	9,64788 E-06	0,193236
Bacteria	*Firmicutes*	*Negativicutes*	*Selenomonadales*	*Acidaminococcaceae*	*Succiniclasticum*	0	1,45043 E-06	0,393769
Bacteria	*Proteobacteria*	*Gammaproteobacteria*	*Aeromonadales*	*Succinivibrionaceae*	*Succinivibrio*	0,00083070 4	0,01705 6563	0,1372
Bacteria	*Proteobacteria*	*Betaproteobacteria*	*Burkholderiales*	*Sutterellaceae*	*Sutterella*	0,00012385 2	0,00029 4118	0,080216
Bacteria	*Proteobacteria*	*Gammaproteobacteria*	*Cardiobacteriales*	*Cardiobacteriaceae*	*Suttonella*	2,35137E-05	0	0,088427
Bacteria	*Synergistetes*	*Synergistia*	*Synergistales*	*Synergistaceae*	*Synergistes*	0,00019178 8	0	0,010871
Bacteria	*Firmicutes*	*Clostridia*	*Clostridiales*	*Lachnospiraceae*	*Syntrophococcus*	1,73549E-05	4,20653 E-05	0,688915

Bacteria	*Bacteroidetes*	*Bacteroidia*	*Bacteroidales*	*Porphyromonadaceae*	*Tannerella*	0,000193495	0,00016178	0,457391
Bacteria	*Proteobacteria*	*Betaproteobacteria*	*Burkholderiales*	*Burkholderiales_incertae_sedis*	*Tepidimonas*	1,57225E-05	2,27524E-05	1
Bacteria	*Actinobacteria*	*Actinobacteria*	*Actinomycetales*	*Intrasporangiaceae*	*Terracoccus*	0	1,20619E-06	0,393769
Bacteria	*Bacteroidetes*	*Sphingobacteriia*	*Sphingobacteriales*	*Chitinophagaceae*	*Terrimonas*	0	0,000004216	0,393769
Bacteria	*Actinobacteria*	*Actinobacteria*	*Actinomycetales*	*Propionibacteriaceae*	*Tessaracoccus*	0,000028578	0,000000642	0,071628
Bacteria	*Proteobacteria*	*Betaproteobacteria*	*Rhodocyclales*	*Rhodocyclaceae*	*Thauera*	0	0,000104633	0,215311
Archaea	*Euryarchaeota*	*Thermoplasmata*	*Thermoplasmatales*	*Thermoplasmatales_incertae_sedis*	*Thermogymnomonas*	0	5,24638E-05	0,063994
Bacteria	*Proteobacteria*	*Gammaproteobacteria*	*Xanthomonadales*	*Xanthomonadaceae*	*Thermomonas*	1,99658E-05	0	0,240955
Bacteria	*Proteobacteria*	*Alphaproteobacteria*	*Rhodobacterales*	*Rhodobacteraceae*	*Thioclava*	0	1,01835E-06	0,215311
Bacteria	*Proteobacteria*	*Gammaproteobacteria*	*Thiotrichales*	*Thiotrichaceae*	*Thiothrix*	0	4,05951E-05	0,11879
Bacteria	*Firmicutes*	*Clostridia*	*Clostridiales*	*Clostridiales_Incertae Sedis XI*	*Tissierella*	6,14874E-05	1,84189E-07	0,298379
Bacteria	*Proteobacteria*	*Gammaproteobacteria*	*Aeromonadales*	*Aeromonadaceae*	*Tolumonas*	0	1,27821E-05	0,393769
Bacteria	*Spirochaetes*	*Spirochaetia*	*Spirochaetales*	*Spirochaetaceae*	*Treponema*	0,000834052	0,035544528	0,00028
Bacteria	*Firmicutes*	*Bacilli*	*Lactobacillales*	*Carnobacteriaceae*	*Trichococcus*	5,01846E-05	4,59281E-05	0,76399
Bacteria	*Actinobacteria*	*Actinobacteria*	*Actinomycetales*	*Actinomycetaceae*	*Trueperella*	0	5,85825E-05	0,063994
Bacteria	*Actinobacteria*	*Actinobacteria*	*Actinomycetales*	*Corynebacteriaceae*	*Turicella*	0	5,91332E-05	0,063994
Bacteria	*Firmicutes*	*Erysipelotrichia*	*Erysipelotrichales*	*Erysipelotrichaceae*	*Turicibacter*	0,000982986	0,000645882	0,1372
Bacteria	*Proteobacteria*	*Betaproteobacteria*	*Burkholderiales*	*Oxalobacteraceae*	*Undibacterium*	0,000281504	4,53981E-05	0,00173
Bacteria	*Tenericutes*	*Mollicutes*	*Mycoplasmatales*	*Mycoplasmataceae*	*Ureaplasma*	0,000005504	0	0,240955

Bacteria	*Proteobacteria*	*Betaproteobacteria*	*Neisseriales*	*Neisseriaceae*	*Uruburuella*	1,71135E-05	0	0,240955
Bacteria	*Firmicutes*	*Bacilli*	*Lactobacillales*	*Enterococcaceae*	*Vagococcus*	3,98297E-05	0	0,031967
Bacteria	*Proteobacteria*	*Deltaproteobacteria*	*Bdellovibrionales*	*Bdellovibrionaceae*	*Vampirovibrio*	0,00272525 6	0,00095 8666	0,004993
Bacteria	*Proteobacteria*	*Betaproteobacteria*	*Burkholderiales*	*Comamonadaceae*	*Variovorax*	3,30239E-06	0	0,240955
Bacteria	*Firmicutes*	*Negativicutes*	*Selenomonadales*	*Veillonellaceae*	*Veillonella*	5,85568E-05	0,00015 7945	0,317468
Bacteria	*Proteobacteria*	*Gammaproteobacteria*	*Vibrionales*	*Vibrionaceae*	*Vibrio*	0	3,06724 E-06	0,393769
Bacteria	*Lentisphaerae*	*Lentisphaeria*	*Victivallales*	*Victivallaceae*	*Victivallis*	3,63057E-05	1,06723 E-05	0,702752
Bacteria	*Proteobacteria*	*Betaproteobacteria*	*Neisseriales*	*Neisseriaceae*	*Vitreoscilla*	1,18128E-05	9,68338 E-06	0,876911
Bacteria	*Bacteroidetes*	*Flavobacteriia*	*Flavobacteriales*	*Flavobacteriaceae*	*Wautersiella*	8,36892E-06	4,15122 E-05	0,567043
Bacteria	*Bacteroidetes*	*Flavobacteriia*	*Flavobacteriales*	*Flavobacteriaceae*	*Weeksella*	3,65782E-05	0	0,240955
Bacteria	*Firmicutes*	*Bacilli*	*Lactobacillales*	*Leuconostocaceae*	*Weissella*	0,00034403 7	3,37919 E-05	0,000637
Bacteria	*Actinobacteria*	*Actinobacteria*	*Actinomycetales*	*Nocardiaceae*	*Williamsia*	0	3,68426 E-06	0,215311
Bacteria	*Proteobacteria*	*Gammaproteobacteria*	*Xanthomonadales*	*Xanthomonadaceae*	*Wohlfahrtiimonas*	0	0,00004 3726	0,215311
Bacteria	*Proteobacteria*	*Epsilonproteobacteria*	*Campylobacterales*	*Helicobacteraceae*	*Wolinella*	0	4,92014 E-06	0,11879
Bacteria	*Proteobacteria*	*Gammaproteobacteria*	*Xanthomonadales*	*Xanthomonadaceae*	*Xanthomonas*	0	3,87335 E-06	0,393769
Bacteria	*Proteobacteria*	*Betaproteobacteria*	*Burkholderiales*	*Comamonadaceae*	*Xenophilus*	1,84328E-06	4,16268 E-06	0,876911
Bacteria	*Bacteroidetes*	*Bacteroidia*	*Bacteroidales*	*Prevotellaceae*	*Xylanibacter*	0	3,84431 E-05	0,003268
Bacteria	*Actinobacteria*	*Actinobacteria*	*Actinomycetales*	*Micrococcaceae*	*Yaniella*	4,32537E-06	0	0,240955
Bacteria	*Actinobacteria*	*Actinobacteria*	*Actinomycetales*	*Micrococcaceae*	*Zhihengliuella*	0	4,22886 E-07	0,393769

Additional file 2. Nasal microbiota composition of piglets from farms MT and MC before (1) and after one cycle (2) or 5 cycles (3) without antimicrobial treatment removal. Mean relative abundance of each phylum (**A**) or family (**B**) in percentage for each sampling group.

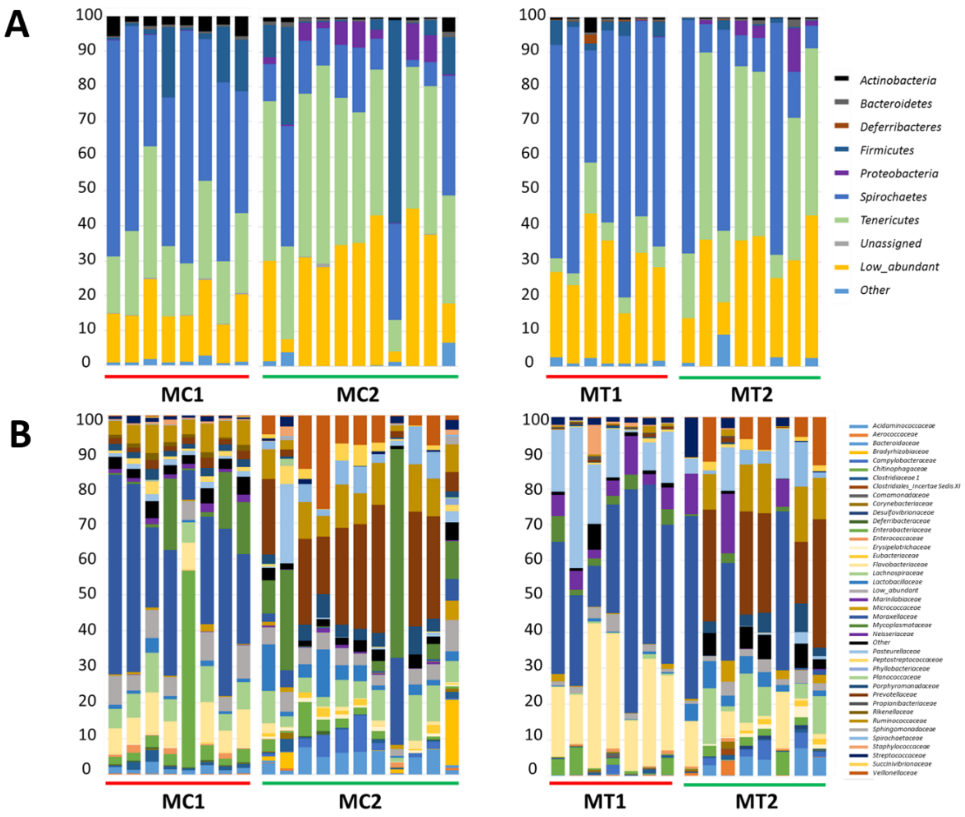

Additional file 3. Frequencies of OTUs assigned at genus level from farm MT at the third sampling (MT3).

Domain	Phylum	Class	Order	Family	Genus	Frequency
						MT3
Bacteria	*Firmicutes*	*Bacilli*	*Lactobacillales*	*Aerococcaceae*	*Abiotrophia*	4,60773E-06
Bacteria	*Actinobacteria*	*Actinobacteria*	*Actinomycetales*	*Micrococcaceae*	*Acaricomes*	3,13176E-07
Bacteria	*Firmicutes*	*Clostridia*	*Clostridiales*	*Ruminococcaceae*	*Acetanaerobacterium*	0,001912339
Bacteria	*Firmicutes*	*Clostridia*	*Clostridiales*	*Lachnospiraceae*	*Acetitomaculum*	2,88336E-05
Bacteria	*Firmicutes*	*Clostridia*	*Clostridiales*	*Ruminococcaceae*	*Acetivibrio*	0,000189811
Bacteria	*Firmicutes*	*Clostridia*	*Clostridiales*	*Peptostreptococcaceae*	*Acetoanaerobium*	1,79506E-06
Bacteria	*Proteobacteria*	*Alphaproteobacteria*	*Rhodospirillales*	*Acetobacteraceae*	*Acetobacter*	1,48E-05
Bacteria	*Tenericutes*	*Mollicutes*	*Acholeplasmatales*	*Acholeplasmataceae*	*Acholeplasma*	1,12397E-05
Bacteria	*Proteobacteria*	*Betaproteobacteria*	*Burkholderiales*	*Alcaligenaceae*	*Achromobacter*	1,79147E-06
Bacteria	*Firmicutes*	*Clostridia*	*Clostridiales*	*Clostridiales_Incertae Sedis XII*	*Acidaminobacter*	2,35088E-07
Bacteria	*Firmicutes*	*Negativicutes*	*Selenomonadales*	*Acidaminococcaceae*	*Acidaminococcus*	0,000414379
Bacteria	*Proteobacteria*	*Alphaproteobacteria*	*Rhodospirillales*	*Acetobacteraceae*	*Acidicaldus*	0
Bacteria	*Proteobacteria*	*Alphaproteobacteria*	*Rhodospirillales*	*Acetobacteraceae*	*Acidisoma*	1,98061E-06
Bacteria	*Proteobacteria*	*Betaproteobacteria*	*Burkholderiales*	*Comamonadaceae*	*Acidovorax*	5,05201E-06
Bacteria	*Proteobacteria*	*Gammaproteobacteria*	*Pseudomonadales*	*Moraxellaceae*	*Acinetobacter*	0,003416526
Bacteria	*Proteobacteria*	*Gammaproteobacteria*	*Pasteurellales*	*Pasteurellaceae*	*Actinobacillus*	0,007520366
Bacteria	*Actinobacteria*	*Actinobacteria*	*Actinomycetales*	*Actinomycetaceae*	*Actinobaculum*	1,61261E-06
Bacteria	*Actinobacteria*	*Actinobacteria*	*Actinomycetales*	*Actinomycetaceae*	*Actinomyces*	8,55758E-05
Bacteria	*Actinobacteria*	*Actinobacteria*	*Coriobacteriales*	*Coriobacteriaceae*	*Adlercreutzia*	1,52868E-05
Bacteria	*Proteobacteria*	*Betaproteobacteria*	*Burkholderiales*	*Alcaligenaceae*	*Advenella*	4,25639E-05
Bacteria	*Firmicutes*	*Bacilli*	*Bacillales*	*Bacillaceae 1*	*Aeribacillus*	0
Bacteria	*Firmicutes*	*Bacilli*	*Lactobacillales*	*Aerococcaceae*	*Aerococcus*	0,000773383
Bacteria	*Proteobacteria*	*Gammaproteobacteria*	*Aeromonadales*	*Aeromonadaceae*	*Aeromonas*	5,67864E-06
Bacteria	*Proteobacteria*	*Alphaproteobacteria*	*Rhizobiales*	*Bradyrhizobiaceae*	*Afipia*	0,000306336
Bacteria	*Proteobacteria*	*Gammaproteobacteria*	*Pasteurellales*	*Pasteurellaceae*	*Aggregatibacter*	7,28457E-06
Bacteria	*Actinobacteria*	*Actinobacteria*	*Actinomycetales*	*Microbacteriaceae*	*Agrococcus*	1,70595E-06
Bacteria	*Verrucomicrobia*	*Verrucomicrobiae*	*Verrucomicrobiales*	*Verrucomicrobiaceae*	*Akkermansia*	0,003390606
Bacteria	*Proteobacteria*	*Betaproteobacteria*	*Burkholderiales*	*Comamonadaceae*	*Albidiferax*	5,93542E-07
Bacteria	*Proteobacteria*	*Betaproteobacteria*	*Burkholderiales*	*Alcaligenaceae*	*Alcaligenes*	2,43695E-06
Bacteria	*Proteobacteria*	*Gammaproteobacteria*	*Alteromonadales*	*Alteromonadaceae*	*Alishewanella*	2,68109E-06

Bacteria	*Bacteroidetes*	*Bacteroidia*	*Bacteroidales*	*Rikenellaceae*	*Alistipes*	0,012255597
Bacteria	*Firmicutes*	*Clostridia*	*Clostridiales*	*Eubacteriaceae*	*Alkalibacter*	2,7233E-06
Bacteria	*Firmicutes*	*Bacilli*	*Lactobacillales*	*Carnobacteriaceae*	*Alkalibacterium*	6,56134E-07
Bacteria	*Bacteroidetes*	*Bacteroidia*	*Bacteroidales*	*Marinilabiaceae*	*Alkaliflexus*	1,67955E-06
Bacteria	*Bacteroidetes*	*Bacteroidia*	*Bacteroidales*	*Marinilabiliaceae*	*Alkalitalea*	1,89479E-07
Bacteria	*Proteobacteria*	*Gammaproteobacteria*	*Pseudomonadales*	*Moraxellaceae*	*Alkanindiges*	1,84975E-05
Bacteria	*Firmicutes*	*Negativicutes*	*Selenomonadales*	*Veillonellaceae*	*Allisonella*	0,000190665
Bacteria	*Firmicutes*	*Erysipelotrichia*	*Erysipelotrichales*	*Erysipelotrichaceae*	*Allobaculum*	1,7687E-07
Bacteria	*Firmicutes*	*Bacilli*	*Lactobacillales*	*Carnobacteriaceae*	*Alloiococcus*	0,000156445
Bacteria	*Bacteroidetes*	*Bacteroidia*	*Bacteroidales*	*Prevotellaceae*	*Alloprevotella*	0,008927882
Bacteria	*Proteobacteria*	*Betaproteobacteria*	*Neisseriales*	*Neisseriaceae*	*Alysiella*	6,56646E-05
Bacteria	*Proteobacteria*	*Alphaproteobacteria*	*Rhodobacterales*	*Rhodobacteraceae*	*Amaricoccus*	0
Bacteria	*Firmicutes*	*Negativicutes*	*Selenomonadales*	*Veillonellaceae*	*Anaeroarcus*	1,323E-07
Bacteria	*Firmicutes*	*Bacilli*	*Bacillales*	*Bacillaceae 1*	*Anaerobacillus*	9,45269E-05
Bacteria	*Firmicutes*	*Clostridia*	*Clostridiales*	*Clostridiaceae 1*	*Anaerobacter*	5,58108E-05
Bacteria	*Proteobacteria*	*Gammaproteobacteria*	*Aeromonadales*	*Succinivibrionaceae*	*Anaerobiospirillum*	4,88727E-05
Bacteria	*Firmicutes*	*Clostridia*	*Clostridiales*	*Clostridiales_Incertae Sedis XI*	*Anaerococcus*	0,000172905
Bacteria	*Firmicutes*	*Clostridia*	*Clostridiales*	*Ruminococcaceae*	*Anaerofilum*	3,71409E-05
Bacteria	*Firmicutes*	*Clostridia*	*Clostridiales*	*Eubacteriaceae*	*Anaerofustis*	1,17408E-05
Bacteria	*Firmicutes*	*Negativicutes*	*Selenomonadales*	*Veillonellaceae*	*Anaeroglobus*	7,90917E-07
Bacteria	*Proteobacteria*	*Deltaproteobacteria*	*Myxococcales*	*Cystobacteraceae*	*Anaeromyxobacter*	7,40719E-07
Bacteria	*Bacteroidetes*	*Bacteroidia*	*Bacteroidales*	*Marinilabiaceae*	*Anaerophaga*	0,0013806
Bacteria	*Tenericutes*	*Mollicutes*	*Anaeroplasmatales*	*Anaeroplasmataceae*	*Anaeroplasma*	0,001630199
Bacteria	*Bacteroidetes*	*Bacteroidia*	*Bacteroidales*	*Bacteroidaceae*	*Anaerorhabdus*	2,7063E-05
Bacteria	*Firmicutes*	*Clostridia*	*Clostridiales*	*Incertae Sedis XI*	*Anaerosphaera*	2,72629E-06
Bacteria	*Firmicutes*	*Clostridia*	*Clostridiales*	*Clostridiaceae 1*	*Anaerosporobacter*	0,000190474
Bacteria	*Firmicutes*	*Clostridia*	*Clostridiales*	*Lachnospiraceae*	*Anaerostipes*	0,000956761
Bacteria	*Firmicutes*	*Clostridia*	*Clostridiales*	*Ruminococcaceae*	*Anaerotruncus*	0,000396041
Bacteria	*Firmicutes*	*Negativicutes*	*Selenomonadales*	*Veillonellaceae*	*Anaerovibrio*	0,004301503
Bacteria	*Firmicutes*	*Clostridia*	*Clostridiales*	*Clostridiales_Incertae Sedis XIII*	*Anaerovorax*	0,000338933
Bacteria	*Firmicutes*	*Bacilli*	*Bacillales*	*Bacillaceae 1*	*Anoxybacillus*	0
Bacteria	*Proteobacteria*	*Betaproteobacteria*	*Burkholderiales*	*Burkholderiales_incertae_sedis*	*Aquabacterium*	2,9986E-06
Bacteria	*Actinobacteria*	*Actinobacteria*	*Actinomycetales*	*Actinomycetaceae*	*Arcanobacterium*	0,000115601
Bacteria	*Proteobacteria*	*Epsilonproteobacteria*	*Campylobacterales*	*Campylobacteraceae*	*Arcobacter*	9,17971E-05
Bacteria	*Armatimonadetes*	*Armatimonadetes_gp2*	*Armatimonadetes_gp2*	*Armatimonadetes_gp2*	*Armatimonadetes_gp 2*	1,26039E-06

Bacteria	*Armatimonadetes*	*Armatimonadetes_gp5*	*Armatimonadetes_gp5*	*Armatimonadetes_gp5*	*Armatimonadetes_gp 5*	0
Bacteria	*Actinobacteria*	*Actinobacteria*	*Actinomycetales*	*Micrococcaceae*	*Arthrobacter*	3,19573E-05
Bacteria	*Tenericutes*	*Mollicutes*	*Anaeroplasmatales*	*Anaeroplasmataceae*	*Asteroleplasma*	0,000520291
Bacteria	*Proteobacteria*	*Alphaproteobacteria*	*Caulobacterales*	*Caulobacteraceae*	*Asticcacaulis*	3,14512E-07
Bacteria	*Firmicutes*	*Bacilli*	*Lactobacillales*	*Carnobacteriaceae*	*Atopobacter*	9,45558E-06
Bacteria	*Actinobacteria*	*Actinobacteria*	*Coriobacteriales*	*Coriobacteriaceae*	*Atopobium*	2,42482E-06
Bacteria	*Firmicutes*	*Bacilli*	*Lactobacillales*	*Carnobacteriaceae*	*Atopostipes*	1,71092E-05
Bacteria	*Proteobacteria*	*Alphaproteobacteria*	*Rhizobiales*	*Aurantimonadaceae*	*Aurantimonas*	0
Bacteria	*Proteobacteria*	*Betaproteobacteria*	*Rhodocyclales*	*Rhodocyclaceae*	*Azoarcus*	1,43961E-06
Bacteria	*Proteobacteria*	*Gammaproteobacteria*	*Pseudomonadales*	*Pseudomonadaceae*	*Azomonas*	4,69764E-07
Bacteria	*Proteobacteria*	*Gammaproteobacteria*	*Pseudomonadales*	*Pseudomonadaceae*	*Azotobacter*	1,32081E-06
Bacteria	*Firmicutes*	*Bacilli*	*Bacillales*	*Bacillaceae 1*	*Bacillus*	7,11495E-05
Bacteria	*Bacteroidetes*	*Bacteroidia*	*Bacteroidales*	*Bacteroidaceae*	*Bacteroides*	0,091638302
Bacteria	*Bacteroidetes*	*Bacteroidia*	*Bacteroidales*	*Porphyromonadaceae*	*Barnesiella*	0,013380466
Bacteria	*Proteobacteria*	*Gammaproteobacteria*	*Pasteurellales*	*Pasteurellaceae*	*Basfia*	1,00793E-06
Bacteria	*Firmicutes*	*Bacilli*	*Lactobacillales*	*Enterococcaceae*	*Bavariicoccus*	1,92163E-06
Bacteria	*Proteobacteria*	*Betaproteobacteria*	*Neisseriales*	*Neisseriaceae*	*Bergeriella*	3,3117E-05
Bacteria	*Bacteroidetes*	*Flavobacteriia*	*Flavobacteriales*	*Flavobacteriaceae*	*Bergeyella*	0,10815999
Bacteria	*Proteobacteria*	*Gammaproteobacteria*	*Pasteurellales*	*Pasteurellaceae*	*Bibersteinia*	1,7234E-05
Bacteria	*Actinobacteria*	*Actinobacteria*	*Bifidobacteriales*	*Bifidobacteriaceae*	*Bifidobacterium*	0,001571031
Bacteria	*Proteobacteria*	*Deltaproteobacteria*	*Desulfovibrionales*	*Desulfovibrionaceae*	*Bilophila*	0,000874161
Bacteria	*Actinobacteria*	*Actinobacteria*	*Actinomycetales*	*Geodermatophilaceae*	*Blastococcus*	6,09754E-07
Bacteria	*Planctomycetes*	*Planctomycetia*	*Planctomycetales*	*Planctomycetaceae*	*Blastopirellula*	4,11959E-07
Bacteria	*Firmicutes*	*Clostridia*	*Clostridiales*	*Lachnospiraceae*	*Blautia*	0,006550138
Bacteria	*Proteobacteria*	*Betaproteobacteria*	*Burkholderiales*	*Alcaligenaceae*	*Bordetella*	0,002945991
Bacteria	*Proteobacteria*	*Alphaproteobacteria*	*Rhizobiales*	*Bradyrhizobiaceae*	*Bosea*	0,000345679
Bacteria	*Actinobacteria*	*Actinobacteria*	*Actinomycetales*	*Dermabacteraceae*	*Brachybacterium*	0,000108336
Bacteria	*Proteobacteria*	*Betaproteobacteria*	*Burkholderiales*	*Comamonadaceae*	*Brachymonas*	1,48864E-05
Bacteria	*Spirochaetes*	*Spirochaetia*	*Spirochaetales*	*Brachyspiraceae*	*Brachyspira*	1,09058E-06
Bacteria	*Proteobacteria*	*Alphaproteobacteria*	*Rhizobiales*	*Bradyrhizobiaceae*	*Bradyrhizobium*	5,91287E-06
Bacteria	*Actinobacteria*	*Actinobacteria*	*Actinomycetales*	*Brevibacteriaceae*	*Brevibacterium*	3,494E-05
Bacteria	*Proteobacteria*	*Alphaproteobacteria*	*Caulobacterales*	*Caulobacteraceae*	*Brevundimonas*	5,3949E-06
Bacteria	*Firmicutes*	*Bacilli*	*Bacillales*	*Listeriaceae*	*Brochothrix*	2,17603E-07
Bacteria	*Actinobacteria*	*Actinobacteria*	*Actinomycetales*	*Propionibacteriaceae*	*Brooklawnia*	1,2883E-06
Bacteria	*Proteobacteria*	*Alphaproteobacteria*	*Rhizobiales*	*Brucellaceae*	*Brucella*	2,14558E-06
Bacteria	*Firmicutes*	*Erysipelotrichia*	*Erysipelotrichales*	*Erysipelotrichaceae*	*Bulleidia*	0,000855421

Bacteria	*Proteobacteria*	*Betaproteobacteria*	*Burkholderiales*	*Burkholderiaceae*	*Burkholderia*	3,84812E-06
Bacteria	*Firmicutes*	*Clostridia*	*Clostridiales*	*Ruminococcaceae*	*Butyricicoccus*	0,00227967
Bacteria	*Bacteroidetes*	*Bacteroidia*	*Bacteroidales*	*Porphyromonadaceae*	*Butyricimonas*	0,002113362
Bacteria	*Firmicutes*	*Clostridia*	*Clostridiales*	*Lachnospiraceae*	*Butyrivibrio*	0,000225843
Bacteria	*Proteobacteria*	*Gammaproteobacteria*	*Thiotrichales*	*Thiotrichales_incertae_sedis*	*Caedibacter*	4,02463E-06
Bacteria	*Proteobacteria*	*Betaproteobacteria*	*Burkholderiales*	*Comamonadaceae*	*Caenimonas*	6,9994E-07
Bacteria	*Proteobacteria*	*Epsilonproteobacteria*	*Campylobacterales*	*Campylobacteraceae*	*Campylobacter*	0,007670846
Bacteria	*Proteobacteria*	*Gammaproteobacteria*	*Candidatus Carsonella*	*Candidatus Carsonella*	*Candidatus Carsonella*	2,93539E-06
Bacteria	*Proteobacteria*	*Betaproteobacteria*	*Burkholderiales*	*Alcaligenaceae*	*Candidimonas*	1,04981E-06
Bacteria	*Bacteroidetes*	*Flavobacteriia*	*Flavobacteriales*	*Flavobacteriaceae*	*Capnocytophaga*	3,10885E-05
Bacteria	*Firmicutes*	*Bacilli*	*Lactobacillales*	*Carnobacteriaceae*	*Carnobacterium*	2,47235E-06
Bacteria	*Firmicutes*	*Bacilli*	*Bacillales*	*Planococcaceae*	*Caryophanon*	3,39E-05
Bacteria	*Proteobacteria*	*Betaproteobacteria*	*Burkholderiales*	*Alcaligenaceae*	*Castellaniella*	2,42521E-06
Bacteria	*Firmicutes*	*Bacilli*	*Lactobacillales*	*Carnobacteriaceae*	*Catellicoccus*	8,24771E-07
Bacteria	*Firmicutes*	*Erysipelotrichia*	*Erysipelotrichales*	*Erysipelotrichaceae*	*Catenibacterium*	0,001230274
Bacteria	*Firmicutes*	*Clostridia*	*Clostridiales*	*Lachnospiraceae*	*Catonella*	1,43478E-05
Bacteria	*Proteobacteria*	*Alphaproteobacteria*	*Caulobacterales*	*Caulobacteraceae*	*Caulobacter*	0,000126173
Bacteria	*Actinobacteria*	*Actinobacteria*	*Actinomycetales*	*Cellulomonadaceae*	*Cellulomonas*	0
Bacteria	*Firmicutes*	*Clostridia*	*Clostridiales*	*Ruminococcaceae*	*Cellulosibacter*	4,39115E-06
Bacteria	*Firmicutes*	*Clostridia*	*Clostridiales*	*Lachnospiraceae*	*Cellulosilyticum*	4,68211E-05
Bacteria	*Proteobacteria*	*Gammaproteobacteria*	*Pseudomonadales*	*Pseudomonadaceae*	*Cellvibrio*	0
Bacteria	*Firmicutes*	*Bacilli*	*Bacillales*	*Bacillaceae 2*	*Cerasibacillus*	0
Bacteria	*Verrucomicrobia*	*Opitutae*	*Puniceicoccales*	*Puniceicoccaceae*	*Cerasicoccus*	6,61427E-07
Bacteria	*Fusobacteria*	*Fusobacteriia*	*Fusobacteriales*	*Fusobacteriaceae*	*Cetobacterium*	2,62431E-05
Bacteria	*Chlamydiae*	*Chlamydiia*	*Chlamydiales*	*Chlamydiaceae*	*Chlamydia*	1,83717E-06
Bacteria	*Bacteroidetes*	*Flavobacteriia*	*Flavobacteriales*	*Flavobacteriaceae*	*Chryseobacterium*	7,02161E-05
Bacteria	*Proteobacteria*	*Alphaproteobacteria*	*Rhodobacterales*	*Rhodobacteraceae*	*Citreicella*	6,28368E-06
Bacteria	*Proteobacteria*	*Gammaproteobacteria*	*Enterobacteriales*	*Enterobacteriaceae*	*Citrobacter*	0,000143868
Bacteria	*Synergistetes*	*Synergistia*	*Synergistales*	*Synergistaceae*	*Cloacibacillus*	0,000125557
Bacteria	*Bacteroidetes*	*Flavobacteriia*	*Flavobacteriales*	*Flavobacteriaceae*	*Cloacibacterium*	5,22489E-06
Bacteria	*Firmicutes*	*Clostridia*	*Clostridiales*	*Ruminococcaceae*	*Clostridium III*	9,21999E-05
Bacteria	*Firmicutes*	*Clostridia*	*Clostridiales*	*Ruminococcaceae*	*Clostridium IV*	0,010333309
Bacteria	*Firmicutes*	*Clostridia*	*Clostridiales*	*Clostridiaceae 1*	*Clostridium sensu stricto*	0,005141397
Bacteria	*Firmicutes*	*Clostridia*	*Clostridiales*	*Peptostreptococcaceae*	*Clostridium XI*	0,00506553
Bacteria	*Fusobacteria*	*Fusobacteriia*	*Fusobacteriales*	*Fusobacteriaceae*	*Clostridium XIX*	5,20751E-06
Bacteria	*Firmicutes*	*Clostridia*	*Clostridiales*	*Lachnospiraceae*	*Clostridium XlVa*	0,015633901

Bacteria	*Firmicutes*	*Clostridia*	*Clostridiales*	*Lachnospiraceae*	*Clostridium XlVb*	0,001181484
Bacteria	*Firmicutes*	*Erysipelotrichia*	*Erysipelotrichales*	*Erysipelotrichaceae*	*Clostridium XVIII*	0,000553577
Bacteria	*Actinobacteria*	*Actinobacteria*	*Coriobacteriales*	*Coriobacteriaceae*	*Collinsella*	0,000312929
Bacteria	*Proteobacteria*	*Betaproteobacteria*	*Burkholderiales*	*Comamonadaceae*	*Comamonas*	4,89991E-05
Bacteria	*Proteobacteria*	*Betaproteobacteria*	*Neisseriales*	*Neisseriaceae*	*Conchiformibius*	0
Bacteria	*Firmicutes*	*Erysipelotrichia*	*Erysipelotrichales*	*Erysipelotrichaceae*	*Coprobacillus*	1,45474E-05
Bacteria	*Firmicutes*	*Clostridia*	*Clostridiales*	*Lachnospiraceae*	*Coprococcus*	0,004920077
Bacteria	*Verrucomicrobia*	*Opitutae*	*Puniceicoccales*	*Puniceicoccaceae*	*Coraliomargarita*	0,000236865
Bacteria	*Actinobacteria*	*Actinobacteria*	*Actinomycetales*	*Corynebacteriaceae*	*Corynebacterium*	0,001642366
Bacteria	*Bacteroidetes*	*Flavobacteriia*	*Flavobacteriales*	*Flavobacteriaceae*	*Cruoricaptor*	4,81073E-07
Bacteria	*Proteobacteria*	*Betaproteobacteria*	*Burkholderiales*	*Burkholderiaceae*	*Cupriavidus*	1,11255E-06
Bacteria	*Actinobacteria*	*Actinobacteria*	*Actinomycetales*	*Microbacteriaceae*	*Curtobacterium*	1,19968E-07
Bacteria	*Proteobacteria*	*Betaproteobacteria*	*Rhodocyclales*	*Rhodocyclaceae*	*Dechloromonas*	1,23617E-06
Bacteria	*Firmicutes*	*Clostridia*	*Clostridiales*	*Defluviitaleaceae*	*Defluviitalea*	8,52982E-05
Bacteria	*Deinococcus-Thermus*	*Deinococci*	*Deinococcales*	*Deinococcaceae*	*Deinococcus*	4,31884E-06
Bacteria	*Proteobacteria*	*Betaproteobacteria*	*Burkholderiales*	*Comamonadaceae*	*Delftia*	0,000286813
Bacteria	*Actinobacteria*	*Actinobacteria*	*Actinomycetales*	*Dermacoccaceae*	*Dermacoccus*	0
Bacteria	*Actinobacteria*	*Actinobacteria*	*Actinomycetales*	*Dermatophilaceae*	*Dermatophilus*	4,83659E-05
Bacteria	*Firmicutes*	*Bacilli*	*Lactobacillales*	*Carnobacteriaceae*	*Desemzia*	3,70359E-07
Bacteria	*Proteobacteria*	*Deltaproteobacteria*	*Desulfobacterales*	*Desulfobulbaceae*	*Desulfobulbus*	8,39774E-07
Bacteria	*Proteobacteria*	*Deltaproteobacteria*	*Desulfovibrionales*	*Desulfomicrobiaceae*	*Desulfomicrobium*	6,72665E-05
Bacteria	*Proteobacteria*	*Deltaproteobacteria*	*Desulfovibrionales*	*Desulfovibrionaceae*	*Desulfovibrio*	0,001785476
Bacteria	*Firmicutes*	*Clostridia*	*Clostridiales*	*Incertae Sedis XI*	*Dethiosulfatibacter*	4,04511E-06
Bacteria	*Proteobacteria*	*Alphaproteobacteria*	*Rhizobiales*	*Hyphomicrobiaceae*	*Devosia*	3,59903E-07
Bacteria	*Firmicutes*	*Negativicutes*	*Selenomonadales*	*Veillonellaceae*	*Dialister*	0,001522556
Bacteria	*Proteobacteria*	*Betaproteobacteria*	*Burkholderiales*	*Comamonadaceae*	*Diaphorobacter*	6,33461E-06
Bacteria	*Actinobacteria*	*Actinobacteria*	*Actinomycetales*	*Dietziaceae*	*Dietzia*	3,00726E-06
Bacteria	*Firmicutes*	*Bacilli*	*Lactobacillales*	*Carnobacteriaceae*	*Dolosigranulum*	5,40318E-06
Bacteria	*Proteobacteria*	*Alphaproteobacteria*	*Rhodospirillales*	*Rhodospirillaceae*	*Dongia*	0
Bacteria	*Firmicutes*	*Clostridia*	*Clostridiales*	*Lachnospiraceae*	*Dorea*	0,002451896
Bacteria	*Bacteroidetes*	*Bacteroidia*	*Bacteroidales*	*Porphyromonadaceae*	*Dysgonomonas*	3,41667E-05
Bacteria	*Actinobacteria*	*Actinobacteria*	*Coriobacteriales*	*Coriobacteriaceae*	*Eggerthella*	3,36848E-05
Bacteria	*Firmicutes*	*Erysipelotrichia*	*Erysipelotrichales*	*Erysipelotrichaceae*	*Eggerthia*	6,55405E-06
Bacteria	*Bacteroidetes*	*Flavobacteriia*	*Flavobacteriales*	*Flavobacteriaceae*	*Elizabethkingia*	0,0005774
Bacteria	*Elusimicrobia*	*Elusimicrobia*	*Elusimicrobiales*	*Elusimicrobiaceae*	*Elusimicrobium*	8,38865E-05
Bacteria	*Bacteroidetes*	*Flavobacteriia*	*Flavobacteriales*	*Flavobacteriaceae*	*Empedobacter*	1,12076E-05

Bacteria	*Proteobacteria*	*Gammaproteobacteria*	*Pseudomonadales*	*Moraxellaceae*	*Enhydrobacter*	4,63058E-05
Bacteria	*Proteobacteria*	*Gammaproteobacteria*	*Enterobacteriales*	*Enterobacteriaceae*	*Enterobacter*	2,06858E-05
Bacteria	*Firmicutes*	*Bacilli*	*Lactobacillales*	*Enterococcaceae*	*Enterococcus*	0,00014701
Bacteria	*Actinobacteria*	*Actinobacteria*	*Coriobacteriales*	*Coriobacteriaceae*	*Enterorhabdus*	1,50689E-05
Bacteria	*Proteobacteria*	*Gammaproteobacteria*	*Enterobacteriales*	*Enterobacteriaceae*	*Erwinia*	1,1238E-07
Bacteria	*Firmicutes*	*Erysipelotrichia*	*Erysipelotrichales*	*Erysipelotrichaceae*	*Erysipelothrix*	9,34488E-05
Bacteria	*Firmicutes*	*Erysipelotrichia*	*Erysipelotrichales*	*Erysipelotrichaceae*	*Erysipelotrichaceae_incertae_sedis*	0,001427226
Bacteria	*Proteobacteria*	*Gammaproteobacteria*	*Enterobacteriales*	*Enterobacteriaceae*	*Escherichia/Shigella*	0,007289037
Bacteria	*Firmicutes*	*Clostridia*	*Clostridiales*	*Ruminococcaceae*	*Ethanoligenens*	4,19046E-05
Bacteria	*Firmicutes*	*Clostridia*	*Clostridiales*	*Eubacteriaceae*	*Eubacterium*	0,003183625
Bacteria	*Firmicutes*	*Bacilli*	*Bacillales*	*Bacillales_Incertae Sedis XII*	*Exiguobacterium*	2,49174E-06
Bacteria	*Firmicutes*	*Bacilli*	*Lactobacillales*	*Aerococcaceae*	*Facklamia*	0,000196841
Bacteria	*Firmicutes*	*Clostridia*	*Clostridiales*	*Ruminococcaceae*	*Faecalibacterium*	0,023073249
Bacteria	*Firmicutes*	*Clostridia*	*Clostridiales*	*Ruminococcaceae*	*Fastidiosipila*	4,81443E-05
Bacteria	*Fibrobacteres*	*Fibrobacteria*	*Fibrobacterales*	*Fibrobacteraceae*	*Fibrobacter*	0,000532675
Bacteria	*Firmicutes*	*Clostridia*	*Clostridiales*	*Peptostreptococcaceae*	*Filifactor*	1,86241E-05
Bacteria	*Bacteroidetes*	*Sphingobacteriia*	*Sphingobacteriales*	*Chitinophagaceae*	*Filimonas*	3,38328E-06
Bacteria	*Firmicutes*	*Clostridia*	*Clostridiales*	*Clostridiales_Incertae Sedis XI*	*Finegoldia*	4,33199E-06
Bacteria	*Bacteroidetes*	*Sphingobacteriia*	*Sphingobacteriales*	*Chitinophagaceae*	*Flavihumibacter*	0
Bacteria	*Bacteroidetes*	*Sphingobacteriia*	*Sphingobacteriales*	*Chitinophagaceae*	*Flavitalea*	3,54901E-07
Bacteria	*Bacteroidetes*	*Flavobacteriia*	*Flavobacteriales*	*Flavobacteriaceae*	*Flavobacterium*	2,88235E-05
Bacteria	*Firmicutes*	*Clostridia*	*Clostridiales*	*Ruminococcaceae*	*Flavonifractor*	0,003223957
Bacteria	*Actinobacteria*	*Actinobacteria*	*Actinomycetales*	*Propionibacteriaceae*	*Friedmanniella*	2,45463E-06
Bacteria	*Fusobacteria*	*Fusobacteriia*	*Fusobacteriales*	*Fusobacteriaceae*	*Fusobacterium*	0,000942762
Bacteria	*Firmicutes*	*Clostridia*	*Clostridiales*	*Clostridiales_Incertae Sedis XI*	*Gallicola*	3,24427E-05
Bacteria	*Actinobacteria*	*Actinobacteria*	*Bifidobacteriales*	*Bifidobacteriaceae*	*Gardnerella*	2,36455E-07
Bacteria	*Bacteroidetes*	*Flavobacteriia*	*Flavobacteriales*	*Flavobacteriaceae*	*Gelidibacter*	5,99838E-07
Bacteria	*Firmicutes*	*Bacilli*	*Bacillales*	*Bacillales_Incertae Sedis XI*	*Gemella*	6,27032E-05
Bacteria	*Planctomycetes*	*Planctomycetia*	*Planctomycetales*	*Planctomycetaceae*	*Gemmata*	0
Bacteria	*Gemmatimonadetes*	*Gemmatimonadetes*	*Gemmatimonadales*	*Gemmatimonadaceae*	*Gemmatimonas*	0
Bacteria	*Firmicutes*	*Clostridia*	*Clostridiales*	*Ruminococcaceae*	*Gemmiger*	0,007849898
Bacteria	*Actinobacteria*	*Actinobacteria*	*Actinomycetales*	*Bogoriellaceae*	*Georgenia*	1,16665E-07
Bacteria	*Bacteroidetes*	*Flavobacteriia*	*Flavobacteriales*	*Flavobacteriaceae*	*Gillisia*	1,89479E-07
Bacteria	*Firmicutes*	*Bacilli*	*Lactobacillales*	*Aerococcaceae*	*Globicatella*	0,000714656
Bacteria	*Actinobacteria*	*Actinobacteria*	*Actinomycetales*	*Glycomycetaceae*	*Glycomyces*	0
Bacteria	*Actinobacteria*	*Actinobacteria*	*Actinomycetales*	*Nocardiaceae*	*Gordonia*	0

Bacteria	*Actinobacteria*	*Actinobacteria*	*Coriobacteriales*	*Coriobacteriaceae*	*Gordonibacter*	9,85107E-06
Bacteria	*Acidobacteria*	*Acidobacteria_Gp16*	*Gp16*	*Gp16*	*Gp16*	0
Bacteria	*Acidobacteria*	*Acidobacteria_Gp6*	*Gp6*	*Gp6*	*Gp6*	0
Bacteria	*Cyanobacteria/Chloroplast*	*Cyanobacteria*	*Family I*	*Family I*	*GpI*	0
Bacteria	*Firmicutes*	*Bacilli*	*Lactobacillales*	*Carnobacteriaceae*	*Granulicatella*	1,88497E-05
Bacteria	*Actinobacteria*	*Actinobacteria*	*Actinomycetales*	*Propionibacteriaceae*	*Granulicoccus*	1,19968E-07
Bacteria	*Firmicutes*	*Clostridia*	*Clostridiales*	*Clostridiales_Incertae Sedis XII*	*Guggenheimella*	1,67241E-05
Bacteria	*Proteobacteria*	*Gammaproteobacteria*	*Pasteurellales*	*Pasteurellaceae*	*Haemophilus*	0,053232799
Bacteria	*Proteobacteria*	*Gammaproteobacteria*	*Enterobacteriales*	*Enterobacteriaceae*	*Hafnia*	1,62125E-06
Bacteria	*Firmicutes*	*Bacilli*	*Bacillales*	*Bacillaceae 2*	*Halalkalibacillus*	1,24711E-07
Bacteria	*Bacteroidetes*	*Bacteroidia*	*Bacteroidales*	*Prevotellaceae*	*Hallella*	0,001057204
Bacteria	*Actinobacteria*	*Actinobacteria*	*Actinomycetales*	*Ruaniaceae*	*Haloactinobacterium*	1,55958E-06
Bacteria	*Firmicutes*	*Bacilli*	*Bacillales*	*Bacillaceae 2*	*Halolactibacillus*	1,2439E-06
Bacteria	*Proteobacteria*	*Gammaproteobacteria*	*Oceanospirillales*	*Halomonadaceae*	*Halomonas*	2,33577E-06
Bacteria	*Proteobacteria*	*Alphaproteobacteria*	*Rhizobiales*	*Methylocystaceae*	*Hansschlegelia*	1,08033E-06
Bacteria	*Actinobacteria*	*Actinobacteria*	*Actinomycetales*	*Dermabacteraceae*	*Helcobacillus*	7,80277E-06
Bacteria	*Firmicutes*	*Clostridia*	*Clostridiales*	*Clostridiales_Incertae Sedis XI*	*Helcococcus*	0,000709202
Bacteria	*Proteobacteria*	*Epsilonproteobacteria*	*Campylobacterales*	*Helicobacteraceae*	*Helicobacter*	7,74393E-05
Bacteria	*Proteobacteria*	*Betaproteobacteria*	*Burkholderiales*	*Oxalobacteraceae*	*Herbaspirillum*	4,11664E-06
Bacteria	*Firmicutes*	*Clostridia*	*Clostridiales*	*Lachnospiraceae*	*Hespellia*	7,68593E-05
Bacteria	*Firmicutes*	*Erysipelotrichia*	*Erysipelotrichales*	*Erysipelotrichaceae*	*Holdemania*	0,001337175
Bacteria	*Firmicutes*	*Clostridia*	*Clostridiales*	*Lachnospiraceae*	*Howardella*	7,35148E-05
Bacteria	*Firmicutes*	*Clostridia*	*Clostridiales*	*Ruminococcaceae*	*Hydrogenoanaerobacterium*	0,000118846
Bacteria	*Proteobacteria*	*Betaproteobacteria*	*Burkholderiales*	*Comamonadaceae*	*Hydrogenophaga*	0
Bacteria	*Proteobacteria*	*Betaproteobacteria*	*Hydrogenophilales*	*Hydrogenophilaceae*	*Hydrogenophilus*	0
Bacteria	*Bacteroidetes*	*Sphingobacteriia*	*Sphingobacteriales*	*Chitinophagaceae*	*Hydrotalea*	0
Bacteria	*Proteobacteria*	*Alphaproteobacteria*	*Rhizobiales*	*Hyphomicrobiaceae*	*Hyphomicrobium*	4,09542E-06
Bacteria	*Actinobacteria*	*Actinobacteria*	*Acidimicrobiales*	*Acidimicrobiaceae*	*Ilumatobacter*	9,35263E-06
Bacteria	*Proteobacteria*	*Alphaproteobacteria*	*Rhodospirillales*	*Rhodospirillaceae*	*Insolitispirillum*	0,00026321
Bacteria	*Firmicutes*	*Bacilli*	*Lactobacillales*	*Carnobacteriaceae*	*Isobaculum*	1,12596E-06
Bacteria	*Actinobacteria*	*Actinobacteria*	*Actinomycetales*	*Promicromonosporaceae*	*Isoptericola*	8,97283E-07
Bacteria	*Actinobacteria*	*Actinobacteria*	*Actinomycetales*	*Intrasporangiaceae*	*Janibacter*	4,97233E-06
Bacteria	*Proteobacteria*	*Betaproteobacteria*	*Burkholderiales*	*Oxalobacteraceae*	*Janthinobacterium*	3,71708E-06
Bacteria	*Firmicutes*	*Bacilli*	*Bacillales*	*Staphylococcaceae*	*Jeotgalicoccus*	0,000156282
Bacteria	*Actinobacteria*	*Actinobacteria*	*Actinomycetales*	*Jiangellaceae*	*Jiangella*	5,91138E-07

Bacteria	*Actinobacteria*	*Actinobacteria*	*Actinomycetales*	*Jonesiaceae*	*Jonesia*	0
Bacteria	*Firmicutes*	*Erysipelotrichia*	*Erysipelotrichales*	*Erysipelotrichaceae*	*Kandleria*	2,3531E-06
Bacteria	*Proteobacteria*	*Betaproteobacteria*	*Burkholderiales*	*Alcaligenaceae*	*Kerstersia*	1,9308E-05
Bacteria	*Proteobacteria*	*Alphaproteobacteria*	*Rhodobacterales*	*Rhodobacteraceae*	*Ketogulonicigenium*	2,646E-07
Bacteria	*Actinobacteria*	*Actinobacteria*	*Actinomycetales*	*Kineosporiaceae*	*Kineococcus*	1,87411E-06
Bacteria	*Proteobacteria*	*Betaproteobacteria*	*Neisseriales*	*Neisseriaceae*	*Kingella*	0,002972686
Bacteria	*Proteobacteria*	*Gammaproteobacteria*	*Enterobacteriales*	*Enterobacteriaceae*	*Klebsiella*	0,00010271
Bacteria	*Actinobacteria*	*Actinobacteria*	*Actinomycetales*	*Micrococcaceae*	*Kocuria*	1,53988E-05
Bacteria	*Firmicutes*	*Bacilli*	*Bacillales*	*Planococcaceae*	*Kurthia*	0,000144274
Bacteria	*Actinobacteria*	*Actinobacteria*	*Actinomycetales*	*Dermacoccaceae*	*Kytococcus*	2,646E-07
Bacteria	*Proteobacteria*	*Alphaproteobacteria*	*Rhizobiales*	*Xanthobacteraceae*	*Labrys*	9,89237E-07
Bacteria	*Firmicutes*	*Bacilli*	*Bacillales*	*Thermoactinomycetaceae 1*	*Laceyella*	2,49423E-07
Bacteria	*Firmicutes*	*Clostridia*	*Clostridiales*	*Lachnospiraceae*	*Lachnoanaerobaculu m*	2,85902E-05
Bacteria	*Firmicutes*	*Clostridia*	*Clostridiales*	*Lachnospiraceae*	*Lachnobacterium*	5,00404E-06
Bacteria	*Firmicutes*	*Clostridia*	*Clostridiales*	*Lachnospiraceae*	*Lachnospira*	0,000756793
Bacteria	*Firmicutes*	*Clostridia*	*Clostridiales*	*Lachnospiraceae*	*Lachnospiracea_incer tae_sedis*	0,01503688
Bacteria	*Firmicutes*	*Bacilli*	*Lactobacillales*	*Carnobacteriaceae*	*Lacticigenium*	0
Bacteria	*Firmicutes*	*Bacilli*	*Lactobacillales*	*Lactobacillaceae*	*Lactobacillus*	0,024329282
Bacteria	*Firmicutes*	*Bacilli*	*Lactobacillales*	*Streptococcaceae*	*Lactococcus*	0,004350049
Bacteria	*Firmicutes*	*Clostridia*	*Clostridiales*	*Lachnospiraceae*	*Lactonifactor*	2,49859E-05
Bacteria	*Firmicutes*	*Bacilli*	*Lactobacillales*	*Streptococcaceae*	*Lactovum*	1,7824E-05
Bacteria	*Proteobacteria*	*Betaproteobacteria*	*Burkholderiales*	*Comamonadaceae*	*Lampropedia*	1,7687E-07
Bacteria	*Proteobacteria*	*Deltaproteobacteria*	*Desulfovibrionales*	*Desulfovibrionaceae*	*Lawsonia*	7,24624E-07
Bacteria	*Proteobacteria*	*Gammaproteobacteria*	*Enterobacteriales*	*Enterobacteriaceae*	*Leclercia*	4,39324E-07
Bacteria	*Fusobacteria*	*Fusobacteriia*	*Fusobacteriales*	*Leptotrichiaceae*	*Leptotrichia*	3,65818E-05
Bacteria	*Actinobacteria*	*Actinobacteria*	*Actinomycetales*	*Microbacteriaceae*	*Leucobacter*	5,50676E-06
Bacteria	*Firmicutes*	*Bacilli*	*Lactobacillales*	*Leuconostocaceae*	*Leuconostoc*	0,000449669
Bacteria	*Proteobacteria*	*Betaproteobacteria*	*Burkholderiales*	*Comamonadaceae*	*Limnohabitans*	5,58802E-06
Bacteria	*Proteobacteria*	*Gammaproteobacteria*	*Pasteurellales*	*Pasteurellaceae*	*Lonepinella*	1,7687E-07
Bacteria	*Chloroflexi*	*Anaerolineae*	*Anaerolineales*	*Anaerolineaceae*	*Longilinea*	3,06095E-06
Bacteria	*Proteobacteria*	*Gammaproteobacteria*	*Xanthomonadales*	*Xanthomonadaceae*	*Luteimonas*	1,76683E-06
Bacteria	*Actinobacteria*	*Actinobacteria*	*Actinomycetales*	*Propionibacteriaceae*	*Luteococcus*	2,85361E-05
Bacteria	*Verrucomicrobia*	*Verrucomicrobiae*	*Verrucomicrobiales*	*Verrucomicrobiaceae*	*Luteolibacter*	2,35088E-07
Bacteria	*Firmicutes*	*Clostridia*	*Clostridiales*	*Gracilibacteraceae*	*Lutispora*	4,28421E-06
Bacteria	*Firmicutes*	*Bacilli*	*Bacillales*	*Planococcaceae*	*Lysinibacillus*	1,00623E-05

Bacteria	*Proteobacteria*	*Gammaproteobacteria*	*Xanthomonadales*	*Xanthomonadaceae*	*Lysobacter*	0
Bacteria	*Firmicutes*	*Bacilli*	*Bacillales*	*Staphylococcaceae*	*Macrococcus*	0
Bacteria	*Bacteroidetes*	*Bacteroidia*	*Bacteroidales*	*Marinilabiliaceae*	*Mangroviflexus*	1,12455E-06
Bacteria	*Proteobacteria*	*Gammaproteobacteria*	*Pasteurellales*	*Pasteurellaceae*	*Mannheimia*	0,002377512
Bacteria	*Firmicutes*	*Bacilli*	*Bacillales*	*Bacillaceae 2*	*Marinococcus*	4,06803E-06
Bacteria	*Proteobacteria*	*Gammaproteobacteria*	*Oceanospirillales*	*Oceanospirillaceae*	*Marinospirillum*	3,75147E-06
Bacteria	*Actinobacteria*	*Actinobacteria*	*Actinomycetales*	*Nocardioidaceae*	*Marmoricola*	1,87906E-06
Bacteria	*Firmicutes*	*Clostridia*	*Clostridiales*	*Lachnospiraceae*	*Marvinbryantia*	5,87408E-05
Bacteria	*Proteobacteria*	*Betaproteobacteria*	*Burkholderiales*	*Oxalobacteraceae*	*Massilia*	1,68336E-06
Bacteria	*Firmicutes*	*Negativicutes*	*Selenomonadales*	*Veillonellaceae*	*Megamonas*	4,75534E-06
Bacteria	*Firmicutes*	*Negativicutes*	*Selenomonadales*	*Veillonellaceae*	*Megasphaera*	0,009434278
Bacteria	*Firmicutes*	*Bacilli*	*Lactobacillales*	*Enterococcaceae*	*Melissococcus*	5,42692E-07
Bacteria	*Bacteroidetes*	*Cytophagia*	*Cytophagales*	*Cytophagaceae*	*Meniscus*	1,89479E-07
Bacteria	*Proteobacteria*	*Alphaproteobacteria*	*Rhizobiales*	*Phyllobacteriaceae*	*Mesorhizobium*	4,1436E-05
Bacteria	*Actinobacteria*	*Actinobacteria*	*Bifidobacteriales*	*Bifidobacteriaceae*	*Metascardovia*	1,94292E-06
Archaea	*Euryarchaeota*	*Methanobacteria*	*Methanobacteriales*	*Methanobacteriaceae*	*Methanobrevibacter*	0,000444732
Archaea	*Euryarchaeota*	*Methanomicrobia*	*Methanosarcinales*	*Methanosaetaceae*	*Methanosaeta*	6,62075E-06
Archaea	*Euryarchaeota*	*Methanobacteria*	*Methanobacteriales*	*Methanobacteriaceae*	*Methanosphaera*	1,97847E-07
Bacteria	*Proteobacteria*	*Betaproteobacteria*	*Methylophilales*	*Methylophilaceae*	*Methylobacillus*	0
Bacteria	*Proteobacteria*	*Alphaproteobacteria*	*Rhizobiales*	*Methylobacteriaceae*	*Methylobacterium*	4,52735E-06
Bacteria	*Proteobacteria*	*Betaproteobacteria*	*Methylophilales*	*Methylophilaceae*	*Methylophilus*	1,79807E-06
Bacteria	*Proteobacteria*	*Alphaproteobacteria*	*Rhizobiales*	*Hyphomicrobiaceae*	*Methylorhabdus*	2,24759E-07
Bacteria	*Actinobacteria*	*Actinobacteria*	*Actinomycetales*	*Microbacteriaceae*	*Microbacterium*	2,72856E-05
Bacteria	*Actinobacteria*	*Actinobacteria*	*Actinomycetales*	*Micrococcaceae*	*Micrococcus*	4,22189E-05
Bacteria	*Actinobacteria*	*Actinobacteria*	*Actinomycetales*	*Nocardiaceae*	*Millisia*	2,23517E-06
Bacteria	*Firmicutes*	*Negativicutes*	*Selenomonadales*	*Veillonellaceae*	*Mitsuokella*	0,000951785
Bacteria	*Firmicutes*	*Clostridia*	*Clostridiales*	*Clostridiales_Incertae Sedis XIII*	*Mogibacterium*	2,56272E-05
Bacteria	*Bacteroidetes*	*Flavobacteriia*	*Flavobacteriales*	*Flavobacteriaceae*	*Moheibacter*	1,19338E-06
Bacteria	*Proteobacteria*	*Gammaproteobacteria*	*Pseudomonadales*	*Moraxellaceae*	*Moraxella*	0,110592342
Bacteria	*Firmicutes*	*Clostridia*	*Clostridiales*	*Lachnospiraceae*	*Moryella*	5,63116E-06
Bacteria	*Bacteroidetes*	*Sphingobacteriia*	*Sphingobacteriales*	*Sphingobacteriaceae*	*Mucilaginibacter*	1,31085E-05
Bacteria	*Deferribacteres*	*Deferribacteres*	*Deferribacterales*	*Deferribacteraceae*	*Mucispirillum*	0,000461103
Bacteria	*Firmicutes*	*Clostridia*	*Clostridiales*	*Incertae Sedis XI*	*Murdochiella*	3,55098E-05
Bacteria	*Actinobacteria*	*Actinobacteria*	*Actinomycetales*	*Mycobacteriaceae*	*Mycobacterium*	3,96592E-06
Bacteria	*Tenericutes*	*Mollicutes*	*Mycoplasmatales*	*Mycoplasmataceae*	*Mycoplasma*	0,002820879
Bacteria	*Bacteroidetes*	*Flavobacteriia*	*Flavobacteriales*	*Flavobacteriaceae*	*Myroides*	1,02751E-05

Bacteria	*Actinobacteria*	*Actinobacteria*	*Actinomycetales*	*Nakamurellaceae*	*Nakamurella*	3,60111E-07
Bacteria	*Firmicutes*	*Clostridia*	*Clostridiales*	*Natranaerovirga*	*Natranaerovirga*	3,53741E-07
Bacteria	*Proteobacteria*	*Betaproteobacteria*	*Burkholderiales*	*Oxalobacteraceae*	*Naxibacter*	9,80466E-06
Bacteria	*Firmicutes*	*Negativicutes*	*Selenomonadales*	*Veillonellaceae*	*Negativicoccus*	1,97847E-07
Bacteria	*Proteobacteria*	*Betaproteobacteria*	*Neisseriales*	*Neisseriaceae*	*Neisseria*	0,019075648
Bacteria	*Actinobacteria*	*Actinobacteria*	*Actinomycetales*	*Micrococcaceae*	*Nesterenkonia*	2,26228E-06
Bacteria	*Proteobacteria*	*Gammaproteobacteria*	*Pasteurellales*	*Pasteurellaceae*	*Nicoletella*	1,21261E-07
Bacteria	*Proteobacteria*	*Alphaproteobacteria*	*Rhizobiales*	*Bradyrhizobiaceae*	*Nitrobacter*	0
Bacteria	*Proteobacteria*	*Betaproteobacteria*	*Nitrosomonadales*	*Nitrosomonadaceae*	*Nitrosospira*	6,73329E-06
Bacteria	*Nitrospirae*	*Nitrospira*	*Nitrospirales*	*Nitrospiraceae*	*Nitrospira*	1,32192E-05
Bacteria	*Actinobacteria*	*Actinobacteria*	*Actinomycetales*	*Nocardioidaceae*	*Nocardioides*	7,57051E-07
Bacteria	*Firmicutes*	*Bacilli*	*Bacillales*	*Staphylococcaceae*	*Nosocomiicoccus*	3,96584E-05
Bacteria	*Proteobacteria*	*Alphaproteobacteria*	*Sphingomonadales*	*Sphingomonadaceae*	*Novosphingobium*	2,6411E-06
Bacteria	*Proteobacteria*	*Gammaproteobacteria*	*Enterobacteriales*	*Enterobacteriaceae*	*Obesumbacterium*	1,24711E-07
Bacteria	*Proteobacteria*	*Gammaproteobacteria*	*Aeromonadales*	*Aeromonadaceae*	*Oceanimonas*	5,99838E-07
Bacteria	*Proteobacteria*	*Alphaproteobacteria*	*Rhizobiales*	*Brucellaceae*	*Ochrobactrum*	0,000312879
Bacteria	*Bacteroidetes*	*Bacteroidia*	*Bacteroidales*	*Porphyromonadaceae*	*Odoribacter*	0,001958987
Bacteria	*Bacteroidetes*	*Bacteroidetes"_incertae_sedis*	*Ohtaekwangia*	*Ohtaekwangia*	*Ohtaekwangia*	8,79912E-06
Bacteria	*Actinobacteria*	*Actinobacteria*	*Actinomycetales*	*Microbacteriaceae*	*Okibacterium*	2,39488E-07
Bacteria	*Proteobacteria*	*Betaproteobacteria*	*Burkholderiales*	*Alcaligenaceae*	*Oligella*	1,04981E-06
Bacteria	*Proteobacteria*	*Alphaproteobacteria*	*Rhizobiales*	*Bradyrhizobiaceae*	*Oligotropha*	3,566E-07
Bacteria	*Actinobacteria*	*Actinobacteria*	*Coriobacteriales*	*Coriobacteriaceae*	*Olsenella*	8,82376E-05
Bacteria	*Firmicutes*	*Clostridia*	*Clostridiales*	*Lachnospiraceae*	*Oribacterium*	0,000597924
Bacteria	*Actinobacteria*	*Actinobacteria*	*Actinomycetales*	*Intrasporangiaceae*	*Ornithinimicrobium*	1,19338E-06
Bacteria	*Bacteroidetes*	*Flavobacteriia*	*Flavobacteriales*	*Flavobacteriaceae*	*Ornithobacterium*	4,01987E-05
Bacteria	*Firmicutes*	*Clostridia*	*Clostridiales*	*Ruminococcaceae*	*Oscillibacter*	0,012080478
Bacteria	*Proteobacteria*	*Gammaproteobacteria*	*Pasteurellales*	*Pasteurellaceae*	*Otariodibacter*	6,36614E-05
Bacteria	*Firmicutes*	*Clostridia*	*Clostridiales*	*Ruminococcaceae*	*Other*	0,023462414
Bacteria	*Firmicutes*	*Clostridia*	*Clostridiales*	*Other*	*Other*	0,018992915
Bacteria	*Firmicutes*	*Clostridia*	*Clostridiales*	*Lachnospiraceae*	*Other*	0,017179887
Bacteria	*Bacteroidetes*	*Sphingobacteriia*	*Sphingobacteriales*	*Chitinophagaceae*	*Other*	0,003471067
Bacteria	*Other*	*Other*	*Other*	*Other*	*Other*	0,002708857
Bacteria	*Firmicutes*	*Other*	*Other*	*Other*	*Other*	0,00464866
Bacteria	*Firmicutes*	*Bacilli*	*Lactobacillales*	*Enterococcaceae*	*Other*	0,004106198
Bacteria	*Firmicutes*	*Negativicutes*	*Selenomonadales*	*Veillonellaceae*	*Other*	0,003875496
Bacteria	*Bacteroidetes*	*Bacteroidia*	*Bacteroidales*	*Porphyromonadaceae*	*Other*	0,003699053

Bacteria	*Bacteroidetes*	*Bacteroidia*	*Bacteroidales*	*Prevotellaceae*	*Other*	0,003111369
Bacteria	*Bacteroidetes*	*Bacteroidia*	*Bacteroidales*	*Other*	*Other*	0,002745992
Bacteria	*Bacteroidetes*	*Other*	*Other*	*Other*	*Other*	0,002741505
Bacteria	*Bacteroidetes*	*Flavobacteriia*	*Flavobacteriales*	*Flavobacteriaceae*	*Other*	0,002209802
Bacteria	*Proteobacteria*	*Alphaproteobacteria*	*Rhodospirillales*	*Rhodospirillaceae*	*Other*	0,002071601
Bacteria	*Proteobacteria*	*Alphaproteobacteria*	*Rhodospirillales*	*Other*	*Other*	0,001568236
Bacteria	*Proteobacteria*	*Gammaproteobacteria*	*Pasteurellales*	*Pasteurellaceae*	*Other*	0,001494329
Bacteria	*Firmicutes*	*Bacilli*	*Lactobacillales*	*Aerococcaceae*	*Other*	0,001462921
Bacteria	*Proteobacteria*	*Betaproteobacteria*	*Burkholderiales*	*Other*	*Other*	0,000892288
Bacteria	*Firmicutes*	*Clostridia*	*Clostridiales*	*Clostridiales_Incertae Sedis XIII*	*Other*	0,000815668
Bacteria	*Firmicutes*	*Erysipelotrichia*	*Erysipelotrichales*	*Erysipelotrichaceae*	*Other*	0,00067816
Bacteria	*Proteobacteria*	*Deltaproteobacteria*	*Desulfovibrionales*	*Desulfovibrionaceae*	*Other*	0,000595466
Unclassified	*Other*	*Other*	*Other*	*Other*	*Other*	0,000592333
Bacteria	*Proteobacteria*	*Deltaproteobacteria*	*Other*	*Other*	*Other*	0,000555976
Bacteria	*Planctomycetes*	*Planctomycetia*	*Planctomycetales*	*Planctomycetaceae*	*Other*	0,000512148
Bacteria	*Bacteroidetes*	*Flavobacteriia*	*Flavobacteriales*	*Other*	*Other*	0,000434328
Bacteria	*Firmicutes*	*Bacilli*	*Bacillales*	*Bacillaceae 1*	*Other*	0,000323123
Bacteria	*Proteobacteria*	*Betaproteobacteria*	*Neisseriales*	*Neisseriaceae*	*Other*	0,000299643
Bacteria	*Actinobacteria*	*Actinobacteria*	*Coriobacteriales*	*Coriobacteriaceae*	*Other*	0,000294396
Bacteria	*Bacteroidetes*	*Sphingobacteriia*	*Sphingobacteriales*	*Sphingobacteriaceae*	*Other*	0,000255323
Bacteria	*Proteobacteria*	*Betaproteobacteria*	*Burkholderiales*	*Sutterellaceae*	*Other*	0,000251773
Bacteria	*Firmicutes*	*Bacilli*	*Lactobacillales*	*Other*	*Other*	0,000232647
Bacteria	*Proteobacteria*	*Alphaproteobacteria*	*Other*	*Other*	*Other*	0,000204901
Bacteria	*Proteobacteria*	*Gammaproteobacteria*	*Pseudomonadales*	*Moraxellaceae*	*Other*	0,000203811
Bacteria	*Proteobacteria*	*Gammaproteobacteria*	*Enterobacteriales*	*Enterobacteriaceae*	*Other*	0,000198581
Bacteria	*Proteobacteria*	*Gammaproteobacteria*	*Other*	*Other*	*Other*	0,000188608
Bacteria	*Actinobacteria*	*Actinobacteria*	*Actinomycetales*	*Other*	*Other*	0,000171436
Bacteria	*Firmicutes*	*Bacilli*	*Lactobacillales*	*Carnobacteriaceae*	*Other*	0,000140604
Bacteria	*Proteobacteria*	*Other*	*Other*	*Other*	*Other*	0,000109903
Bacteria	*Firmicutes*	*Clostridia*	*Clostridiales*	*Clostridiales_Incertae Sedis XI*	*Other*	0,000106459
Bacteria	*Verrucomicrobia*	*Opitutae*	*Puniceicoccales*	*Puniceicoccaceae*	*Other*	8,14409E-05
Bacteria	*Proteobacteria*	*Betaproteobacteria*	*Other*	*Other*	*Other*	7,96636E-05
Bacteria	*Firmicutes*	*Negativicutes*	*Selenomonadales*	*Acidaminococcaceae*	*Other*	7,61761E-05
Bacteria	*Bacteroidetes*	*Bacteroidia*	*Bacteroidales*	*Marinilabiaceae*	*Other*	6,92969E-05
Bacteria	*Fusobacteria*	*Fusobacteriia*	*Fusobacteriales*	*Leptotrichiaceae*	*Other*	6,53654E-05
Bacteria	*Firmicutes*	*Clostridia*	*Other*	*Other*	*Other*	6,3211E-05

Bacteria	*Actinobacteria*	*Actinobacteria*	*Bifidobacteriales*	*Bifidobacteriaceae*	*Other*	5,91148E-05
Bacteria	*Firmicutes*	*Clostridia*	*Clostridiales*	*Peptococcaceae 1*	*Other*	5,81455E-05
Bacteria	*Firmicutes*	*Negativicutes*	*Selenomonadales*	*Other*	*Other*	5,69042E-05
Bacteria	*Firmicutes*	*Clostridia*	*Clostridiales*	*Clostridiaceae 1*	*Other*	5,40275E-05
Bacteria	*Lentisphaerae*	*Other*	*Other*	*Other*	*Other*	5,1341E-05
Bacteria	*Proteobacteria*	*Alphaproteobacteria*	*Rhizobiales*	*Other*	*Other*	4,98868E-05
Bacteria	*Spirochaetes*	*Spirochaetia*	*Spirochaetales*	*Spirochaetaceae*	*Other*	4,76015E-05
Bacteria	*Firmicutes*	*Bacilli*	*Lactobacillales*	*Lactobacillaceae*	*Other*	4,24476E-05
Bacteria	*Firmicutes*	*Clostridia*	*Clostridiales*	*Incertae Sedis XI*	*Other*	4,13367E-05
Bacteria	*Firmicutes*	*Clostridia*	*Clostridiales*	*Gracilibacteraceae*	*Other*	3,7104E-05
Bacteria	*Bacteroidetes*	*Bacteroidia*	*Bacteroidales*	*Rikenellaceae*	*Other*	3,312E-05
Bacteria	*Firmicutes*	*Bacilli*	*Other*	*Other*	*Other*	3,25271E-05
Bacteria	*Proteobacteria*	*Betaproteobacteria*	*Burkholderiales*	*Comamonadaceae*	*Other*	2,51143E-05
Bacteria	*Proteobacteria*	*Betaproteobacteria*	*Burkholderiales*	*Alcaligenaceae*	*Other*	2,33602E-05
Bacteria	*Proteobacteria*	*Epsilonproteobacteria*	*Campylobacterales*	*Other*	*Other*	2,08777E-05
Bacteria	*Firmicutes*	*Bacilli*	*Bacillales*	*Other*	*Other*	1,7717E-05
Bacteria	*Firmicutes*	*Clostridia*	*Clostridiales*	*Peptostreptococcaceae*	*Other*	1,63231E-05
Archaea	*Other*	*Other*	*Other*	*Other*	*Other*	1,34014E-05
Bacteria	*Proteobacteria*	*Deltaproteobacteria*	*Desulfovibrionales*	*Other*	*Other*	1,20657E-05
Bacteria	*Actinobacteria*	*Actinobacteria*	*Actinomycetales*	*Microbacteriaceae*	*Other*	1,14066E-05
Bacteria	*Actinobacteria*	*Actinobacteria*	*Actinomycetales*	*Propionibacteriaceae*	*Other*	1,12707E-05
Bacteria	*Actinobacteria*	*Actinobacteria*	*Actinomycetales*	*Micrococcaceae*	*Other*	1,02283E-05
Bacteria	*Firmicutes*	*Bacilli*	*Lactobacillales*	*Streptococcaceae*	*Other*	1,00232E-05
Bacteria	*Fusobacteria*	*Fusobacteriia*	*Fusobacteriales*	*Fusobacteriaceae*	*Other*	9,25737E-06
Bacteria	*Spirochaetes*	*Spirochaetia*	*Spirochaetales*	*Other*	*Other*	9,22536E-06
Bacteria	*Proteobacteria*	*Alphaproteobacteria*	*Rhizobiales*	*Brucellaceae*	*Other*	7,81617E-06
Bacteria	*Proteobacteria*	*Gammaproteobacteria*	*Pseudomonadales*	*Other*	*Other*	5,2227E-06
Bacteria	*Proteobacteria*	*Betaproteobacteria*	*Nitrosomonadales*	*Nitrosomonadaceae*	*Other*	5,11784E-06
Bacteria	*Actinobacteria*	*Actinobacteria*	*Actinomycetales*	*Corynebacteriaceae*	*Other*	4,30003E-06
Bacteria	*Proteobacteria*	*Alphaproteobacteria*	*Sphingomonadales*	*Other*	*Other*	3,719E-06
Bacteria	*Proteobacteria*	*Alphaproteobacteria*	*Rhizobiales*	*Phyllobacteriaceae*	*Other*	3,61352E-06
Bacteria	*Actinobacteria*	*Actinobacteria*	*Other*	*Other*	*Other*	3,52248E-06
Bacteria	*Proteobacteria*	*Epsilonproteobacteria*	*Other*	*Other*	*Other*	3,38783E-06
Archaea	*Euryarchaeota*	*Other*	*Other*	*Other*	*Other*	3,35107E-06
Archaea	*Crenarchaeota*	*Thermoprotei*	*Other*	*Other*	*Other*	2,30538E-06
Bacteria	*Firmicutes*	*Clostridia*	*Clostridiales*	*Eubacteriaceae*	*Other*	2,27745E-06

Bacteria	*Proteobacteria*	*Alphaproteobacteria*	*Rhizobiales*	*Bradyrhizobiaceae*	*Other*	1,95552E-06
Bacteria	*Actinobacteria*	*Actinobacteria*	*Actinomycetales*	*Intrasporangiaceae*	*Other*	1,90042E-06
Bacteria	*Proteobacteria*	*Betaproteobacteria*	*Burkholderiales*	*Burkholderiales_incertae_sedis*	*Other*	1,89958E-06
Bacteria	*Bacteroidetes*	*Sphingobacteriia*	*Sphingobacteriales*	*Other*	*Other*	1,77002E-06
Bacteria	*Firmicutes*	*Bacilli*	*Bacillales*	*Bacillaceae 2*	*Other*	1,69856E-06
Bacteria	*Proteobacteria*	*Betaproteobacteria*	*Burkholderiales*	*Oxalobacteraceae*	*Other*	1,60405E-06
Bacteria	*Synergistetes*	*Synergistia*	*Synergistales*	*Synergistaceae*	*Other*	1,42899E-06
Bacteria	*Firmicutes*	*Bacilli*	*Bacillales*	*Staphylococcaceae*	*Other*	1,279E-06
Bacteria	*Proteobacteria*	*Gammaproteobacteria*	*Aeromonadales*	*Succinivibrionaceae*	*Other*	1,27563E-06
Bacteria	*Cyanobacteria/Chloroplast*	*Chloroplast*	*Chloroplast*	*Chloroplast*	*Other*	1,18357E-06
Bacteria	*Tenericutes*	*Mollicutes*	*Other*	*Other*	*Other*	1,17544E-06
Bacteria	*Proteobacteria*	*Alphaproteobacteria*	*Sphingomonadales*	*Sphingomonadaceae*	*Other*	1,1747E-06
Bacteria	*Actinobacteria*	*Actinobacteria*	*Actinomycetales*	*Nocardioidaceae*	*Other*	1,16906E-06
Bacteria	*Bacteroidetes*	*Cytophagia*	*Cytophagales*	*Other*	*Other*	1,05901E-06
Bacteria	*Proteobacteria*	*Betaproteobacteria*	*Methylophilales*	*Methylophilaceae*	*Other*	9,261E-07
Bacteria	*Actinobacteria*	*Actinobacteria*	*Actinomycetales*	*Dermacoccaceae*	*Other*	9,15442E-07
Bacteria	*Firmicutes*	*Bacilli*	*Bacillales*	*Planococcaceae*	*Other*	7,65635E-07
Bacteria	*Proteobacteria*	*Betaproteobacteria*	*Rhodocyclales*	*Rhodocyclaceae*	*Other*	7,18066E-07
Bacteria	*Verrucomicrobia*	*Verrucomicrobiae*	*Verrucomicrobiales*	*Verrucomicrobiaceae*	*Other*	7,07852E-07
Bacteria	*Proteobacteria*	*Deltaproteobacteria*	*Desulfovibrionales*	*Desulfohalobiaceae*	*Other*	7,07481E-07
Bacteria	*Proteobacteria*	*Alphaproteobacteria*	*Rhodobacterales*	*Rhodobacteraceae*	*Other*	7,03883E-07
Bacteria	*Actinobacteria*	*Actinobacteria*	*Actinomycetales*	*Dermatophilaceae*	*Other*	5,99838E-07
Bacteria	*Proteobacteria*	*Alphaproteobacteria*	*Rhizobiales*	*Hyphomicrobiaceae*	*Other*	5,91786E-07
Bacteria	*Actinobacteria*	*Actinobacteria*	*Acidimicrobiales*	*Other*	*Other*	4,79871E-07
Bacteria	*Actinobacteria*	*Actinobacteria*	*Actinomycetales*	*Dermabacteraceae*	*Other*	4,25115E-07
Bacteria	*Verrucomicrobia*	*Other*	*Other*	*Other*	*Other*	4,24568E-07
Bacteria	*Proteobacteria*	*Gammaproteobacteria*	*Pseudomonadales*	*Pseudomonadaceae*	*Other*	3,8616E-07
Bacteria	*Proteobacteria*	*Gammaproteobacteria*	*Xanthomonadales*	*Xanthomonadaceae*	*Other*	2,39935E-07
Bacteria	*Bacteroidetes*	*Cytophagia*	*Cytophagales*	*Cytophagaceae*	*Other*	2,35088E-07
Bacteria	*Actinobacteria*	*Actinobacteria*	*Actinomycetales*	*Actinomycetaceae*	*Other*	2,24759E-07
Bacteria	*Chloroflexi*	*Other*	*Other*	*Other*	*Other*	2,23591E-07
Bacteria	*Actinobacteria*	*Actinobacteria*	*Actinomycetales*	*Nocardiaceae*	*Other*	2,23591E-07
Bacteria	*Proteobacteria*	*Epsilonproteobacteria*	*Campylobacterales*	*Helicobacteraceae*	*Other*	1,7687E-07
Bacteria	*Proteobacteria*	*Gammaproteobacteria*	*Aeromonadales*	*Aeromonadaceae*	*Other*	1,7687E-07
Bacteria	*Proteobacteria*	*Gammaproteobacteria*	*Aeromonadales*	*Other*	*Other*	1,7687E-07
Bacteria	*Actinobacteria*	*Actinobacteria*	*Actinomycetales*	*Promicromonosporaceae*	*Other*	1,323E-07

Bacteria	*Proteobacteria*	*Alphaproteobacteria*	*Caulobacterales*	*Other*	*Other*	1,21261E-07
Bacteria	*Proteobacteria*	*Gammaproteobacteria*	*Chromatiales*	*Other*	*Other*	1,21261E-07
Bacteria	*Proteobacteria*	*Alphaproteobacteria*	*Rhodospirillales*	*Acetobacteraceae*	*Other*	1,19968E-07
Bacteria	*Firmicutes*	*Bacilli*	*Lactobacillales*	*Leuconostocaceae*	*Other*	1,16665E-07
Bacteria	*Proteobacteria*	*Alphaproteobacteria*	*Sphingomonadales*	*Erythrobacteraceae*	*Other*	0
Bacteria	*Actinobacteria*	*Actinobacteria*	*Actinomycetales*	*Kineosporiaceae*	*Other*	0
Bacteria	*Actinobacteria*	*Actinobacteria*	*Actinomycetales*	*Geodermatophilaceae*	*Other*	0
Bacteria	*Proteobacteria*	*Deltaproteobacteria*	*Myxococcales*	*Polyangiaceae*	*Other*	0
Bacteria	*Proteobacteria*	*Alphaproteobacteria*	*Caulobacterales*	*Caulobacteraceae*	*Other*	0
Bacteria	*Proteobacteria*	*Gammaproteobacteria*	*Vibrionales*	*Vibrionaceae*	*Other*	0
Bacteria	*Proteobacteria*	*Betaproteobacteria*	*Burkholderiales*	*Oxalobacteraceae*	*Oxalicibacterium*	3,35386E-06
Bacteria	*Proteobacteria*	*Betaproteobacteria*	*Burkholderiales*	*Oxalobacteraceae*	*Oxalobacter*	0,000100905
Bacteria	*Proteobacteria*	*Betaproteobacteria*	*Burkholderiales*	*Alcaligenaceae*	*Paenalcaligenes*	7,35898E-06
Bacteria	*Proteobacteria*	*Alphaproteobacteria*	*Rhizobiales*	*Brucellaceae*	*Paenochrobactrum*	7,9139E-07
Bacteria	*Bacteroidetes*	*Bacteroidia*	*Bacteroidales*	*Porphyromonadaceae*	*Paludibacter*	0,000503084
Bacteria	*Proteobacteria*	*Gammaproteobacteria*	*Enterobacteriales*	*Enterobacteriaceae*	*Pantoea*	2,94886E-06
Bacteria	*Firmicutes*	*Clostridia*	*Clostridiales*	*Ruminococcaceae*	*Papillibacter*	0,000167083
Bacteria	*Bacteroidetes*	*Bacteroidia*	*Bacteroidales*	*Porphyromonadaceae*	*Parabacteroides*	0,008610952
Bacteria	*Chlamydiae*	*Chlamydiia*	*Chlamydiales*	*Parachlamydiaceae*	*Parachlamydia*	1,91045E-06
Bacteria	*Proteobacteria*	*Alphaproteobacteria*	*Rhodobacterales*	*Rhodobacteraceae*	*Paracoccus*	3,98496E-05
Bacteria	*Actinobacteria*	*Actinobacteria*	*Coriobacteriales*	*Coriobacteriaceae*	*Paraeggerthella*	3,42997E-05
Bacteria	*Firmicutes*	*Bacilli*	*Lactobacillales*	*Lactobacillaceae*	*Paralactobacillus*	2,53891E-05
Bacteria	*Proteobacteria*	*Gammaproteobacteria*	*Pseudomonadales*	*Moraxellaceae*	*Paraperlucidibaca*	1,80056E-07
Bacteria	*Bacteroidetes*	*Bacteroidia*	*Bacteroidales*	*Prevotellaceae*	*Paraprevotella*	0,006097732
Bacteria	*Proteobacteria*	*Betaproteobacteria*	*Burkholderiales*	*Alcaligenaceae*	*Parapusillimonas*	2,93513E-07
Bacteria	*Bacteroidetes*	*Sphingobacteriia*	*Sphingobacteriales*	*Chitinophagaceae*	*Parasegetibacter*	1,16665E-07
Bacteria	*Firmicutes*	*Clostridia*	*Clostridiales*	*Lachnospiraceae*	*Parasporobacterium*	1,96708E-05
Bacteria	*Proteobacteria*	*Betaproteobacteria*	*Burkholderiales*	*Sutterellaceae*	*Parasutterella*	0,002389247
Bacteria	*Parcubacteria*	*Parcubacteria_genera_incertae_sedis*	*Parcubacteria_genera_incertae_sedis*	*Parcubacteria_genera_incertae_sedis*	*Parcubacteria_genera_incertae_sedis*	2,95893E-07
Bacteria	*Firmicutes*	*Clostridia*	*Clostridiales*	*Clostridiales_Incertae Sedis XI*	*Parvimonas*	4,37167E-05
Bacteria	*Proteobacteria*	*Gammaproteobacteria*	*Pasteurellales*	*Pasteurellaceae*	*Pasteurella*	8,22216E-05
Bacteria	*Firmicutes*	*Bacilli*	*Lactobacillales*	*Lactobacillaceae*	*Pediococcus*	3,05774E-06
Bacteria	*Bacteroidetes*	*Sphingobacteriia*	*Sphingobacteriales*	*Sphingobacteriaceae*	*Pedobacter*	1,8518E-07
Bacteria	*Proteobacteria*	*Alphaproteobacteria*	*Rhizobiales*	*Hyphomicrobiaceae*	*Pedomicrobium*	0
Bacteria	*Proteobacteria*	*Alphaproteobacteria*	*Rhizobiales*	*Hyphomicrobiaceae*	*Pelagibacterium*	1,31964E-06
Bacteria	*Proteobacteria*	*Betaproteobacteria*	*Burkholderiales*	*Comamonadaceae*	*Pelomonas*	4,32704E-06

Bacteria	*Firmicutes*	*Clostridia*	*Clostridiales*	*Syntrophomonadaceae*	*Pelospora*	1,00079E-06
Bacteria	*Firmicutes*	*Clostridia*	*Clostridiales*	*Peptococcaceae 1*	*Peptococcus*	0,000245346
Bacteria	*Firmicutes*	*Clostridia*	*Clostridiales*	*Clostridiales_Incertae Sedis XI*	*Peptoniphilus*	6,47157E-05
Bacteria	*Firmicutes*	*Clostridia*	*Clostridiales*	*Peptostreptococcaceae*	*Peptostreptococcaceae_incertae_sedis*	7,30179E-06
Bacteria	*Firmicutes*	*Clostridia*	*Clostridiales*	*Peptostreptococcaceae*	*Peptostreptococcus*	9,75244E-05
Bacteria	*Proteobacteria*	*Deltaproteobacteria*	*Bdellovibrionales*	*Bacteriovoracaceae*	*Peredibacter*	4,71531E-06
Bacteria	*Proteobacteria*	*Gammaproteobacteria*	*Pseudomonadales*	*Moraxellaceae*	*Perlucidibaca*	2,75926E-06
Bacteria	*Bacteroidetes*	*Bacteroidia*	*Bacteroidales*	*Porphyromonadaceae*	*Petrimonas*	2,86517E-05
Bacteria	*Firmicutes*	*Negativicutes*	*Selenomonadales*	*Acidaminococcaceae*	*Phascolarctobacterium*	0,016452076
Bacteria	*Proteobacteria*	*Deltaproteobacteria*	*Myxococcales*	*Phaselicystidaceae*	*Phaselicystis*	0
Bacteria	*Proteobacteria*	*Alphaproteobacteria*	*Caulobacterales*	*Caulobacteraceae*	*Phenylobacterium*	4,36629E-06
Bacteria	*Actinobacteria*	*Actinobacteria*	*Actinomycetales*	*Intrasporangiaceae*	*Phycicoccus*	3,54683E-06
Bacteria	*Proteobacteria*	*Alphaproteobacteria*	*Rhizobiales*	*Phyllobacteriaceae*	*Phyllobacterium*	2,38124E-05
Bacteria	*Firmicutes*	*Bacilli*	*Lactobacillales*	*Enterococcaceae*	*Pilibacter*	4,13481E-07
Bacteria	*Planctomycetes*	*Planctomycetia*	*Planctomycetales*	*Planctomycetaceae*	*Pirellula*	8,72979E-07
Bacteria	*Planctomycetes*	*Planctomycetia*	*Planctomycetales*	*Planctomycetaceae*	*Planctomyces*	2,5137E-06
Bacteria	*Bacteroidetes*	*Flavobacteriia*	*Flavobacteriales*	*Flavobacteriaceae*	*Planobacterium*	0,000741298
Bacteria	*Firmicutes*	*Bacilli*	*Bacillales*	*Planococcaceae*	*Planococcaceae_incertae_sedis*	3,28929E-05
Bacteria	*Firmicutes*	*Bacilli*	*Bacillales*	*Planococcaceae*	*Planococcus*	2,03565E-06
Bacteria	*Firmicutes*	*Bacilli*	*Bacillales*	*Planococcaceae*	*Planomicrobium*	3,34716E-06
Bacteria	*Proteobacteria*	*Betaproteobacteria*	*Burkholderiales*	*Burkholderiaceae*	*Polynucleobacter*	9,6826E-07
Bacteria	*Bacteroidetes*	*Cytophagia*	*Cytophagales*	*Cytophagaceae*	*Pontibacter*	9,33319E-07
Bacteria	*Proteobacteria*	*Alphaproteobacteria*	*Sphingomonadales*	*Erythrobacteraceae*	*Porphyrobacter*	0
Bacteria	*Bacteroidetes*	*Bacteroidia*	*Bacteroidales*	*Porphyromonadaceae*	*Porphyromonas*	0,00022231
Bacteria	*Bacteroidetes*	*Bacteroidia*	*Bacteroidales*	*Prevotellaceae*	*Prevotella*	0,082812046
Bacteria	*Bacteroidetes*	*Bacteroidetes"_incertae_sedis*	*Prolixibacter*	*Prolixibacter*	*Prolixibacter*	1,7687E-07
Bacteria	*Actinobacteria*	*Actinobacteria*	*Actinomycetales*	*Propionibacteriaceae*	*Propionibacterium*	0,000361595
Bacteria	*Actinobacteria*	*Actinobacteria*	*Actinomycetales*	*Propionibacteriaceae*	*Propionicicella*	2,32347E-07
Bacteria	*Actinobacteria*	*Actinobacteria*	*Actinomycetales*	*Propionibacteriaceae*	*Propionicimonas*	1,75743E-06
Bacteria	*Fusobacteria*	*Fusobacteriia*	*Fusobacteriales*	*Fusobacteriaceae*	*Propionigenium*	0
Bacteria	*Actinobacteria*	*Actinobacteria*	*Actinomycetales*	*Propionibacteriaceae*	*Propionimicrobium*	3,95695E-07
Bacteria	*Verrucomicrobia*	*Verrucomicrobiae*	*Verrucomicrobiales*	*Verrucomicrobiaceae*	*Prosthecobacter*	6,40563E-06
Bacteria	*Firmicutes*	*Clostridia*	*Clostridiales*	*Clostridiaceae 1*	*Proteiniclasticum*	1,30981E-05
Bacteria	*Bacteroidetes*	*Bacteroidia*	*Bacteroidales*	*Porphyromonadaceae*	*Proteiniphilum*	2,39935E-06

Bacteria	*Firmicutes*	*Clostridia*	*Clostridiales*	*Peptostreptococcaceae*	*Proteocatella*	2,45218E-05
Bacteria	*Proteobacteria*	*Gammaproteobacteria*	*Enterobacteriales*	*Enterobacteriaceae*	*Proteus*	8,2689E-06
Bacteria	*Firmicutes*	*Clostridia*	*Clostridiales*	*Lachnospiraceae*	*Pseudobutyrivibrio*	2,7175E-05
Bacteria	*Proteobacteria*	*Alphaproteobacteria*	*Rhizobiales*	*Brucellaceae*	*Pseudochrobactrum*	2,28769E-07
Bacteria	*Actinobacteria*	*Actinobacteria*	*Actinomycetales*	*Microbacteriaceae*	*Pseudoclavibacter*	5,68438E-07
Bacteria	*Firmicutes*	*Clostridia*	*Clostridiales*	*Ruminococcaceae*	*Pseudoflavonifractor*	0,001604096
Bacteria	*Proteobacteria*	*Gammaproteobacteria*	*Pseudomonadales*	*Pseudomonadaceae*	*Pseudomonas*	8,79561E-05
Bacteria	*Firmicutes*	*Clostridia*	*Clostridiales*	*Eubacteriaceae*	*Pseudoramibacter*	1,03132E-06
Bacteria	*Bacteroidetes*	*Sphingobacteriia*	*Sphingobacteriales*	*Sphingobacteriaceae*	*Pseudosphingobacterium*	2,18362E-06
Bacteria	*Proteobacteria*	*Alphaproteobacteria*	*Rhodobacterales*	*Rhodobacteraceae*	*Pseudovibrio*	0
Bacteria	*Proteobacteria*	*Gammaproteobacteria*	*Xanthomonadales*	*Xanthomonadaceae*	*Pseudoxanthomonas*	0
Bacteria	*Fusobacteria*	*Fusobacteriia*	*Fusobacteriales*	*Fusobacteriaceae*	*Psychrilyobacter*	1,46132E-07
Bacteria	*Firmicutes*	*Bacilli*	*Bacillales*	*Planococcaceae*	*Psychrobacillus*	2,21346E-06
Bacteria	*Proteobacteria*	*Gammaproteobacteria*	*Pseudomonadales*	*Moraxellaceae*	*Psychrobacter*	0,022248244
Bacteria	*Firmicutes*	*Negativicutes*	*Selenomonadales*	*Veillonellaceae*	*Psychrosinus*	1,89479E-07
Bacteria	*Proteobacteria*	*Betaproteobacteria*	*Burkholderiales*	*Alcaligenaceae*	*Pusillimonas*	0
Bacteria	*Synergistetes*	*Synergistia*	*Synergistales*	*Synergistaceae*	*Pyramidobacter*	4,68017E-05
Bacteria	*Proteobacteria*	*Betaproteobacteria*	*Burkholderiales*	*Burkholderiaceae*	*Ralstonia*	8,18424E-05
Bacteria	*Proteobacteria*	*Gammaproteobacteria*	*Enterobacteriales*	*Enterobacteriaceae*	*Raoultella*	0,000120494
Bacteria	*Actinobacteria*	*Actinobacteria*	*Actinomycetales*	*Micrococcaceae*	*Renibacterium*	0
Bacteria	*Proteobacteria*	*Gammaproteobacteria*	*Chromatiales*	*Chromatiaceae*	*Rheinheimera*	5,6733E-06
Bacteria	*Proteobacteria*	*Gammaproteobacteria*	*Pseudomonadales*	*Pseudomonadaceae*	*Rhizobacter*	0
Bacteria	*Proteobacteria*	*Alphaproteobacteria*	*Rhizobiales*	*Rhizobiaceae*	*Rhizobium*	0,000234806
Bacteria	*Proteobacteria*	*Alphaproteobacteria*	*Rhodobacterales*	*Rhodobacteraceae*	*Rhodobacter*	4,91867E-06
Bacteria	*Actinobacteria*	*Actinobacteria*	*Actinomycetales*	*Nocardiaceae*	*Rhodococcus*	8,48941E-06
Bacteria	*Proteobacteria*	*Alphaproteobacteria*	*Rhodospirillales*	*Acetobacteraceae*	*Rhodopila*	2,34882E-06
Bacteria	*Bacteroidetes*	*Flavobacteriia*	*Flavobacteriales*	*Flavobacteriaceae*	*Riemerella*	6,03136E-06
Bacteria	*Bacteroidetes*	*Bacteroidia*	*Bacteroidales*	*Rikenellaceae*	*Rikenella*	5,28292E-06
Bacteria	*Firmicutes*	*Clostridia*	*Clostridiales*	*Lachnospiraceae*	*Robinsoniella*	0,00024207
Bacteria	*Firmicutes*	*Clostridia*	*Clostridiales*	*Lachnospiraceae*	*Roseburia*	0,016973011
Bacteria	*Proteobacteria*	*Alphaproteobacteria*	*Rhodospirillales*	*Acetobacteraceae*	*Roseomonas*	4,54106E-06
Bacteria	*Proteobacteria*	*Alphaproteobacteria*	*Rhodobacterales*	*Rhodobacteraceae*	*Roseovarius*	0
Bacteria	*Actinobacteria*	*Actinobacteria*	*Actinomycetales*	*Micrococcaceae*	*Rothia*	0,01665775
Bacteria	*Proteobacteria*	*Alphaproteobacteria*	*Rhodobacterales*	*Rhodobacteraceae*	*Rubellimicrobium*	3,29631E-06
Bacteria	*Proteobacteria*	*Betaproteobacteria*	*Burkholderiales*	*Burkholderiales_incertae_sedis*	*Rubrivivax*	1,81787E-05
Bacteria	*Actinobacteria*	*Actinobacteria*	*Rubrobacterales*	*Rubrobacteraceae*	*Rubrobacter*	2,13866E-06

Bacteria	*Proteobacteria*	*Gammaproteobacteria*	*Aeromonadales*	*Succinivibrionaceae*	*Ruminobacter*	2,61988E-05
Bacteria	*Firmicutes*	*Clostridia*	*Clostridiales*	*Ruminococcaceae*	*Ruminococcus*	0,00584075
Bacteria	*Firmicutes*	*Clostridia*	*Clostridiales*	*Lachnospiraceae*	*Ruminococcus2*	0,004145569
Bacteria	*Candidatus Saccharibacteria*	*Saccharibacteria_genera_incertae_sedis*	*Saccharibacteria_genera_incertae_sedis*	*Saccharibacteria_genera_incertae_sedis*	*Saccharibacteria_genera_incertae_sedis*	0,000338023
Bacteria	*Firmicutes*	*Clostridia*	*Clostridiales*	*Ruminococcaceae*	*Saccharofermentans*	2,87402E-05
Bacteria	*Actinobacteria*	*Actinobacteria*	*Actinomycetales*	*Pseudonocardiaceae*	*Saccharomonospora*	3,59903E-07
Bacteria	*Firmicutes*	*Bacilli*	*Bacillales*	*Staphylococcaceae*	*Salinicoccus*	6,29717E-06
Bacteria	*Bacteroidetes*	*Flavobacteriia*	*Flavobacteriales*	*Flavobacteriaceae*	*Salinimicrobium*	0
Bacteria	*Proteobacteria*	*Gammaproteobacteria*	*Enterobacteriales*	*Enterobacteriaceae*	*Salmonella*	6,56298E-06
Bacteria	*Firmicutes*	*Clostridia*	*Clostridiales*	*Clostridiaceae 1*	*Sarcina*	5,35664E-05
Bacteria	*Proteobacteria*	*Betaproteobacteria*	*Burkholderiales*	*Comamonadaceae*	*Schlegelella*	1,80056E-07
Bacteria	*Firmicutes*	*Negativicutes*	*Selenomonadales*	*Veillonellaceae*	*Schwartzia*	0,001440899
Bacteria	*Firmicutes*	*Clostridia*	*Clostridiales*	*Clostridiales_Incertae Sedis XI*	*Sedimentibacter*	9,5542E-07
Bacteria	*Bacteroidetes*	*Sphingobacteriia*	*Sphingobacteriales*	*Chitinophagaceae*	*Sediminibacterium*	3,61663E-06
Bacteria	*Firmicutes*	*Negativicutes*	*Selenomonadales*	*Veillonellaceae*	*Selenomonas*	0,004450666
Bacteria	*Actinobacteria*	*Actinobacteria*	*Actinomycetales*	*Beutenbergiaceae*	*Serinibacter*	5,1946E-05
Bacteria	*Proteobacteria*	*Gammaproteobacteria*	*Pseudomonadales*	*Pseudomonadaceae*	*Serpens*	2,51194E-07
Bacteria	*Proteobacteria*	*Gammaproteobacteria*	*Enterobacteriales*	*Enterobacteriaceae*	*Serratia*	1,95515E-06
Bacteria	*Firmicutes*	*Erysipelotrichia*	*Erysipelotrichales*	*Erysipelotrichaceae*	*Sharpea*	1,18228E-07
Bacteria	*Proteobacteria*	*Gammaproteobacteria*	*Alteromonadales*	*Shewanellaceae*	*Shewanella*	2,75926E-06
Bacteria	*Proteobacteria*	*Betaproteobacteria*	*Rhodocyclales*	*Rhodocyclaceae*	*Shinella*	1,19968E-07
Bacteria	*Firmicutes*	*Clostridia*	*Clostridiales*	*Lachnospiraceae*	*Shuttleworthia*	2,03197E-06
Bacteria	*Proteobacteria*	*Betaproteobacteria*	*Neisseriales*	*Neisseriaceae*	*Simonsiella*	7,86657E-07
Bacteria	*Proteobacteria*	*Betaproteobacteria*	*Burkholderiales*	*Comamonadaceae*	*Simplicispira*	2,39935E-06
Bacteria	*Firmicutes*	*Bacilli*	*Bacillales*	*Bacillaceae 2*	*Sinobaca*	1,04981E-06
Bacteria	*Proteobacteria*	*Alphaproteobacteria*	*Rhodospirillales*	*Rhodospirillaceae*	*Skermanella*	0
Bacteria	*Actinobacteria*	*Actinobacteria*	*Coriobacteriales*	*Coriobacteriaceae*	*Slackia*	2,46535E-05
Bacteria	*Fusobacteria*	*Fusobacteriia*	*Fusobacteriales*	*Leptotrichiaceae*	*Sneathia*	1,16754E-06
Bacteria	*Firmicutes*	*Bacilli*	*Bacillales*	*Planococcaceae*	*Solibacillus*	2,42521E-07
Bacteria	*Actinobacteria*	*Actinobacteria*	*Solirubrobacterales*	*Solirubrobacteraceae*	*Solirubrobacter*	0
Bacteria	*Bacteroidetes*	*Flavobacteriia*	*Flavobacteriales*	*Flavobacteriaceae*	*Soonwooa*	1,55905E-05
Bacteria	*Chloroflexi*	*Thermomicrobia*	*Sphaerobacterales*	*Sphaerobacteraceae*	*Sphaerobacter*	4,84267E-06
Bacteria	*Spirochaetes*	*Spirochaetia*	*Spirochaetales*	*Spirochaetaceae*	*Sphaerochaeta*	0,000675312
Bacteria	*Bacteroidetes*	*Sphingobacteriia*	*Sphingobacteriales*	*Sphingobacteriaceae*	*Sphingobacterium*	1,6181E-05
Bacteria	*Proteobacteria*	*Alphaproteobacteria*	*Sphingomonadales*	*Sphingomonadaceae*	*Sphingobium*	6,77944E-06
Bacteria	*Proteobacteria*	*Alphaproteobacteria*	*Sphingomonadales*	*Sphingomonadaceae*	*Sphingomonas*	0,000160447

Bacteria	*Proteobacteria*	*Alphaproteobacteria*	*Sphingomonadales*	*Sphingomonadaceae*	*Sphingosinicella*	7,66291E-07
Bacteria	*Spirochaetes*	*Spirochaetia*	*Spirochaetales*	*Spirochaetaceae*	*Spirochaeta*	1,7687E-07
Bacteria	*Firmicutes*	*Clostridia*	*Clostridiales*	*Peptostreptococcaceae*	*Sporacetigenium*	1,85711E-05
Bacteria	*Firmicutes*	*Clostridia*	*Clostridiales*	*Ruminococcaceae*	*Sporobacter*	0,001074167
Bacteria	*Firmicutes*	*Bacilli*	*Bacillales*	*Sporolactobacillaceae*	*Sporolactobacillaceae _incertae_sedis*	1,8518E-07
Bacteria	*SR1*	*SR1_genera_incertae_sedis*	*SR1_genera_incertae_sedis*	*SR1_genera_incertae_sedis*	*SR1_genera_incertae _sedis*	1,88632E-05
Bacteria	*Proteobacteria*	*Alphaproteobacteria*	*Sphingomonadales*	*Sphingomonadaceae*	*Stakelama*	0
Bacteria	*Firmicutes*	*Bacilli*	*Bacillales*	*Staphylococcaceae*	*Staphylococcus*	0,000338795
Bacteria	*Proteobacteria*	*Gammaproteobacteria*	*Xanthomonadales*	*Xanthomonadaceae*	*Stenotrophomonas*	2,56271E-05
Bacteria	*Fusobacteria*	*Fusobacteriia*	*Fusobacteriales*	*Leptotrichiaceae*	*Streptobacillus*	4,24695E-05
Bacteria	*Firmicutes*	*Bacilli*	*Lactobacillales*	*Streptococcaceae*	*Streptococcus*	0,010249989
Bacteria	*Actinobacteria*	*Actinobacteria*	*Actinomycetales*	*Streptomycetaceae*	*Streptomyces*	2,2448E-06
Bacteria	*Cyanobacteria/Chloroplast*	*Chloroplast*	*Chloroplast*	*Chloroplast*	*Streptophyta*	0,000413259
Bacteria	*Verrucomicrobia*	*Subdivision3*	*Subdivision3_genera_incertae_sedis*	*Subdivision3_genera_incertae _sedis*	*Subdivision3_genera_ incertae_sedis*	1,76108E-06
Bacteria	*Verrucomicrobia*	*Subdivision5*	*Subdivision5_genera_incertae_sedis*	*Subdivision5_genera_incertae _sedis*	*Subdivision5_genera_ incertae_sedis*	0,000168423
Bacteria	*Firmicutes*	*Clostridia*	*Clostridiales*	*Ruminococcaceae*	*Subdoligranulum*	3,11616E-05
Bacteria	*Firmicutes*	*Negativicutes*	*Selenomonadales*	*Acidaminococcaceae*	*Succiniclasticum*	3,56926E-07
Bacteria	*Firmicutes*	*Negativicutes*	*Selenomonadales*	*Acidaminococcaceae*	*Succinispira*	1,7687E-07
Bacteria	*Proteobacteria*	*Gammaproteobacteria*	*Aeromonadales*	*Succinivibrionaceae*	*Succinivibrio*	0,004408282
Bacteria	*Proteobacteria*	*Betaproteobacteria*	*Burkholderiales*	*Sutterellaceae*	*Sutterella*	0,003204398
Bacteria	*Proteobacteria*	*Gammaproteobacteria*	*Cardiobacteriales*	*Cardiobacteriaceae*	*Suttonella*	9,36591E-06
Bacteria	*Synergistetes*	*Synergistia*	*Synergistales*	*Synergistaceae*	*Synergistes*	1,24233E-05
Bacteria	*Firmicutes*	*Clostridia*	*Clostridiales*	*Lachnospiraceae*	*Syntrophococcus*	8,40861E-06
Bacteria	*Bacteroidetes*	*Bacteroidia*	*Bacteroidales*	*Porphyromonadaceae*	*Tannerella*	0,000153079
Bacteria	*Proteobacteria*	*Betaproteobacteria*	*Burkholderiales*	*Burkholderiales_incertae_sedis*	*Tepidimonas*	0
Bacteria	*Actinobacteria*	*Actinobacteria*	*Actinomycetales*	*Propionibacteriaceae*	*Tessaracoccus*	9,71553E-05
Bacteria	*Firmicutes*	*Bacilli*	*Lactobacillales*	*Enterococcaceae*	*Tetragenococcus*	1,21228E-06
Bacteria	*Proteobacteria*	*Betaproteobacteria*	*Rhodocyclales*	*Rhodocyclaceae*	*Thauera*	2,39935E-07
Bacteria	*Firmicutes*	*Bacilli*	*Bacillales*	*Bacillales_Incertae Sedis X*	*Thermicanus*	0
Archaea	*Euryarchaeota*	*Thermoplasmata*	*Thermoplasmatales*	*Thermoplasmatales_incertae_ sedis*	*Thermogymnomonas*	1,30418E-05
Bacteria	*Actinobacteria*	*Thermoleophilia*	*Thermoleophilales*	*Thermoleophilaceae*	*Thermoleophilum*	0

Bacteria	*Proteobacteria*	*Gammaproteobacteria*	*Xanthomonadales*	*Xanthomonadaceae*	*Thermomonas*	0
Bacteria	*Proteobacteria*	*Gammaproteobacteria*	*Thiotrichales*	*Piscirickettsiaceae*	*Thioalkalimicrobium*	7,82941E-07
Bacteria	*Proteobacteria*	*Gammaproteobacteria*	*Thiotrichales*	*Thiotrichaceae*	*Thiothrix*	3,11916E-06
Bacteria	*Firmicutes*	*Clostridia*	*Clostridiales*	*Clostridiales_Incertae Sedis XI*	*Tissierella*	3,72507E-06
Bacteria	*Proteobacteria*	*Gammaproteobacteria*	*Aeromonadales*	*Aeromonadaceae*	*Tolumonas*	5,59839E-07
Bacteria	*Actinobacteria*	*Actinobacteria*	*Actinomycetales*	*Corynebacterineae_incertae_sedis*	*Tomitella*	1,56588E-07
Bacteria	*Spirochaetes*	*Spirochaetia*	*Spirochaetales*	*Spirochaetaceae*	*Treponema*	0,011113654
Bacteria	*Firmicutes*	*Bacilli*	*Lactobacillales*	*Carnobacteriaceae*	*Trichococcus*	2,86886E-05
Bacteria	*Deinococcus-Thermus*	*Deinococci*	*Deinococcales*	*Trueperaceae*	*Truepera*	0
Bacteria	*Actinobacteria*	*Actinobacteria*	*Actinomycetales*	*Actinomycetaceae*	*Trueperella*	3,51495E-05
Bacteria	*Actinobacteria*	*Actinobacteria*	*Actinomycetales*	*Corynebacteriaceae*	*Turicella*	5,19489E-06
Bacteria	*Firmicutes*	*Erysipelotrichia*	*Erysipelotrichales*	*Erysipelotrichaceae*	*Turicibacter*	0,000898162
Bacteria	*Proteobacteria*	*Betaproteobacteria*	*Burkholderiales*	*Oxalobacteraceae*	*Undibacterium*	4,98164E-06
Bacteria	*Firmicutes*	*Bacilli*	*Bacillales*	*Planococcaceae*	*Ureibacillus*	3,53741E-07
Bacteria	*Proteobacteria*	*Betaproteobacteria*	*Neisseriales*	*Neisseriaceae*	*Uruburuella*	1,35808E-05
Bacteria	*Firmicutes*	*Bacilli*	*Lactobacillales*	*Enterococcaceae*	*Vagococcus*	3,50502E-05
Bacteria	*Proteobacteria*	*Deltaproteobacteria*	*Bdellovibrionales*	*Bdellovibrionaceae*	*Vampirovibrio*	0,003219813
Bacteria	*Proteobacteria*	*Betaproteobacteria*	*Burkholderiales*	*Comamonadaceae*	*Variovorax*	1,09447E-05
Bacteria	*Firmicutes*	*Negativicutes*	*Selenomonadales*	*Veillonellaceae*	*Veillonella*	0,0002448
Bacteria	*Proteobacteria*	*Gammaproteobacteria*	*Vibrionales*	*Vibrionaceae*	*Vibrio*	5,20885E-06
Bacteria	*Lentisphaerae*	*Lentisphaeria*	*Victivallales*	*Victivallaceae*	*Victivallis*	0,000360707
Bacteria	*Actinobacteria*	*Actinobacteria*	*Actinomycetales*	*Micromonosporaceae*	*Virgisporangium*	2,23591E-07
Bacteria	*Proteobacteria*	*Betaproteobacteria*	*Neisseriales*	*Neisseriaceae*	*Vitreoscilla*	2,0353E-06
Bacteria	*Bacteroidetes*	*Flavobacteriia*	*Flavobacteriales*	*Flavobacteriaceae*	*Wautersiella*	4,93524E-05
Bacteria	*Bacteroidetes*	*Flavobacteriia*	*Flavobacteriales*	*Flavobacteriaceae*	*Weeksella*	0
Bacteria	*Firmicutes*	*Bacilli*	*Lactobacillales*	*Leuconostocaceae*	*Weissella*	0,000169603
Bacteria	*Actinobacteria*	*Actinobacteria*	*Actinomycetales*	*Nocardiaceae*	*Williamsia*	0
Bacteria	*Proteobacteria*	*Gammaproteobacteria*	*Xanthomonadales*	*Xanthomonadaceae*	*Wohlfahrtiimonas*	3,42297E-06
Bacteria	*Proteobacteria*	*Epsilonproteobacteria*	*Campylobacterales*	*Helicobacteraceae*	*Wolinella*	1,19753E-06
Bacteria	*Proteobacteria*	*Gammaproteobacteria*	*Xanthomonadales*	*Xanthomonadaceae*	*Xanthomonas*	0
Bacteria	*Bacteroidetes*	*Bacteroidia*	*Bacteroidales*	*Prevotellaceae*	*Xylanibacter*	0,0001259
Bacteria	*Actinobacteria*	*Actinobacteria*	*Actinomycetales*	*Micrococcaceae*	*Yaniella*	1,01713E-05
Bacteria	*Proteobacteria*	*Gammaproteobacteria*	*Enterobacteriales*	*Enterobacteriaceae*	*Yokenella*	1,25471E-06
Bacteria	*Actinobacteria*	*Actinobacteria*	*Actinomycetales*	*Micrococcaceae*	*Zhihengliuella*	1,21261E-07
Bacteria	*Proteobacteria*	*Betaproteobacteria*	*Rhodocyclales*	*Rhodocyclaceae*	*Zoogloea*	1,43961E-06
